# Home Visits and the Use of Routine and Emergency Postpartum Care Among Low-Income People: A Secondary Analysis of a Randomized Clinical Trial

**DOI:** 10.1001/jamanetworkopen.2024.51605

**Published:** 2024-12-23

**Authors:** Slawa Rokicki, Dea Oviedo, Nicolas Perreault, Chloe Zera, Alecia J. McGregor, Mary Ann Bates, R. Annetta Zhou, Katherine Baicker, Margaret A. McConnell

**Affiliations:** 1 Department of Health Behavior, Society, and Policy, Rutgers School of Public Health, Piscataway, New Jersey; 2 Department of Global Health and Population, Harvard T.H. Chan School of Public Health, Boston, Massachusetts; 3 Division of Maternal Fetal Medicine, Department of Obstetrics and Gynecology, Beth Israel Deaconess Medical Center, Boston, Massachusetts; 4 Department of Obstetrics, Gynecology and Reproductive Biology, Harvard Medical School, Boston, Massachusetts; 5 Abdul Latif Jameel Poverty Action Lab, Massachusetts Institute of Technology, Cambridge; 6 Cradle-to-Career Data System, State of California, Sacramento; 7 RAND Corporation, Boston, Massachusetts; 8 National Bureau of Economic Research, Cambridge, Massachusetts; 9 University of Chicago, Chicago, Illinois; 10 Department of Health Policy and Management, Harvard T.H. Chan School of Public Health, Boston, Massachusetts

## Abstract

**Importance:**

Access to appropriate postpartum care is essential for improving maternal health outcomes and promoting maternal health equity.

**Objective:**

To analyze the impact of the Nurse-Family Partnership (NFP) home visiting program on use of routine and emergency postpartum care.

**Design, Setting, and Participants:**

This study was a secondary analysis of a randomized clinical trial that enrolled eligible participants between 2016 and 2020 to receive NFP or usual care from a South Carolina Medicaid program. Participants were nulliparous pregnant individuals at less than 28 weeks’ gestation at enrollment who were enrolled in Medicaid during pregnancy or delivery. Analysis was performed February 2, 2023, to July 16, 2024.

**Intervention:**

Participants were enrolled in a 2:1 ratio to receive NFP, an established model of nurse home visiting, or usual care. NFP visits are conducted prenatally and for up to 2 years post partum.

**Main Outcomes and Measures:**

This study assessed one of the trial’s preregistered secondary outcomes: attendance at a routine postpartum health care visit before 12 weeks post partum. Other exploratory outcomes included outpatient care use, hospitalization, and emergent, nonemergent, and any use of emergency department (ED) health services at 12 weeks and 1 year post partum. Subgroup analyses examined individuals who underwent cesarean delivery, had chronic disease, experienced pregnancy complications, reported non-Hispanic Black race and ethnicity, were socially vulnerable, or were unaffected by COVID-19.

**Results:**

Among 5670 participants enrolled, 4877 were analyzed (median [IQR] age, 21 [19-25] years), including 3261 who received NFP and 1616 who received usual care. By race and ethnicity, the sample included 259 Hispanic individuals (5.7%); 56 non-Hispanic Asian, Indigenous, or Native Hawaiian and Pacific Islander individuals (1.2%); 2535 non-Hispanic Black individuals (55.4%); 1587 non-Hispanic White individuals (34.7%); and 141 individuals (3.1%) with more than 1 race reported (and non-Hispanic ethnicity). There was no significant difference in attendance of routine postpartum visit at 12 weeks between NFP and usual care groups (63.8% vs 64.2%; adjusted difference [AD] −0.3 [95% CI, −3.2 to 2.5] percentage points). In exploratory analyses, the NFP group was less likely to have a visit to the ED without admission (AD, −2.5 [95% CI, −4.8 to −0.1] percentage points) in the first 12 weeks post partum. Other outcomes, including hospitalizations, outpatient visits, and emergent and nonemergent ED visits, were not different between intervention and control groups. There were also no statistically significant differences within subgroups.

**Conclusions and Relevance:**

In this secondary analysis of a randomized clinical trial of nulliparous pregnant individuals with low income, nurse home visiting did not increase use of routine postpartum care in the immediate or extended postpartum periods, even among those with greater health challenges. Nurse home visiting was associated with reduced ED use. More research is needed on how to improve continuity of perinatal care for low-income populations.

**Trial Registration:**

ClinicalTrials.gov Identifier: NCT03360539

## Key Points

**Question:**

Does prenatal and postpartum visitation by nurses change utilization of postpartum care among low-income people?

**Findings:**

In this secondary analysis of a randomized clinical trial among 4877 Medicaid-eligible nulliparous pregnant individuals, rates of postpartum visit attendance were not statistically different between the nurse-visited intervention group and the usual care group. The intervention reduced use of the emergency department in the first 12 weeks post partum.

**Meaning:**

These findings suggest that while home visiting was not effective at increasing engagement in routine postpartum care, it may be effective at reducing postpartum visits to the emergency department.

## Teaser

This secondary analysis of a randomized clinical trial assesses the effect of the Nurse-Family Partnership (NFP) home visiting program on use of routine and emergency postpartum care among Medicaid-enrolled individuals in North Carolina.

## Introduction

Rates of maternal morbidity and mortality in the US are markedly higher than other comparable countries, with stark and persistent racial and socioeconomic disparities.^1,2^ The postpartum period is increasingly recognized as a time of elevated maternal risk; more than half of maternal deaths occur after the day of birth, and one-third occur after 7 days post partum.^3^ The American College of Obstetricians and Gynecologists (ACOG) recommends at least 1 routine postpartum visit within 12 weeks of delivery to address maternal physical and mental well-being.^4^ More recently, ACOG guidelines have emphasized the value of earlier and more frequent contacts with the goal of transitioning pregnant people from obstetrical to primary care, in particular for those experiencing chronic conditions or challenges with mental health.^5^

Many lower-income pregnant people do not receive adequate and timely routine medical care in the postpartum period, even in the first 60 days of delivery historically covered by pregnancy Medicaid.^6,7,8,9^ A 2022 systematic review found that less than 65% of Medicaid-insured individuals receive a routine postpartum visit.^10^ Even among individuals with chronic disease or complications during pregnancy, postpartum visit attendance remains low.^10^ Compared with their commercially insured counterparts, Medicaid-insured postpartum people are more likely to use the emergency department (ED) for care that could be managed in a nonemergency setting, which is disruptive to families and costly to the health care system.^9,11,12^ Identifying effective interventions that target lower-income individuals to increase use of outpatient preventive care and reduce nonemergent ED use in the postpartum period are important public health priorities.^13,14^

Nurse home visiting programs aim to improve maternal and child health among socially and economically disadvantaged families through health screening, education, and referrals to health and social services during regular home visits throughout pregnancy and post partum. These programs may increase engagement in preventive and follow-up postpartum care, for example, through home visitors informing individuals on where and when to seek care, reducing an individual’s barriers to accessing care, and assessing an individual’s health to identify follow-up care that would be beneficial.^7^ Prenatal educational and social support interventions, such as group-based prenatal care and lay health worker interventions, have shown some promise in supporting postpartum attendance rates.^15,16^ While home visiting has been proposed as a way to strengthen continuity of care during the postpartum period^13^ and evidence suggests home visiting services can contribute to reduced hospital care–seeking during the postpartum period for infants,^17,18^ more evidence is needed on the impact of nurse home visiting on maternal postpartum care use. In this secondary analysis of a randomized clinical trial, we evaluated the impact of the Nurse Family Partnership (NFP), an established model of home visiting, on routine and emergency care use in the immediate and extended postpartum period.

## Methods

This randomized clinical trial was approved by the Harvard T.H. Chan School of Public Health Institutional Review Board. Permissions were also obtained from cooperating institutions.^19^ All participants provided written informed consent. The trial protocol and statistical analysis plan are provided in Supplement 1. This study was registered on ClinicalTrials.gov (identifier: NCT03360539) and in the American Economic Association’s randomized clinical trial registry (AEARCTR-0001039). This study is reported following the Consolidated Standards of Reporting Trials (CONSORT) reporting guideline.

### Trial Design

We conducted an individually randomized clinical trial in which study participants were randomly assigned either to a treatment group that was offered access to NFP or to a control group that had access to usual care but not NFP. Individuals were eligible if they were pregnant with less than 28 weeks’ gestation, nulliparous, income-eligible for Medicaid during pregnancy or delivery, and resided in an NFP-served catchment area. Individuals who were 14 years and younger, incarcerated, or in a lockdown facility were excluded.

### Recruitment and Randomization

Pregnant individuals were referred to NFP through their local health care practitioners, schools, or Medicaid, or they self-referred, via 1 of 9 NFP implementing sites embedded in government agencies and hospital systems throughout South Carolina.^20^ Trained NFP staff assessed potential participants’ eligibility. Eligible, consenting participants completed a short survey. Participants were then randomly assigned on-the-spot in a 2:1 treatment-to-control ratio using computer-assisted randomization software (SurveyCTO; Dobility). Investigators included questions about participants’ race and ethnicity to assess the program’s potential influence on racial disparities in maternal and child outcomes; participants were asked to self-identify their race and ethnicity from a list of prespecified options (Hispanic or Latina or not Hispanic or Latina for ethnicity and American Indian or Alaska Native, Asian, Black or African American, Native Hawaiian or Other Pacific Islander, or White for race). Owing to small sample sizes, American Indian or Alaska Native, Asian, and Native Hawaiian or Other Pacific Islander were combined into a single group.

### Intervention and Control

Participants randomized to the intervention group were offered NFP services throughout pregnancy and up to 24 months after delivery, or until they discontinued participation. NFP is a prenatal and early childhood home visiting program for lower-income first-time birthing parents and their families. During visits, nurses conduct a variety of activities, including education sessions, health assessments, and referrals to health care and social programs.^20^ Nurses also encourage health care use when needed,^21,22^ and in some agencies, nurses have access to participants’ electronic medical records. Services were provided in English and Spanish, and translation services were available for participants speaking other languages. Control group members received usual care in South Carolina, which included all community and medical services to which they would have otherwise been entitled, including up to 2 postpartum home visits paid for by Medicaid. All study participants were provided with a list of available community resources.^19^

### Data Sources and Sample

To assess outcomes, we matched study participants to vital records, Medicaid administrative claims data, and hospital discharge records via a probabilistic match based on name, race and ethnicity, social security number, birth date, and Medicaid identifier. To assess receipt of NFP home visiting services, we matched participants to NFP programmatic data. We assessed uptake of Medicaid-funded home visiting using Medicaid claims. The analytical sample was restricted to participants with an index live birth in matched vital records within 120 days of the expected delivery date reported on the baseline survey and who were enrolled in Medicaid either during pregnancy or at delivery.

### Outcomes

The trial protocol specified 3 primary and 54 secondary outcomes.^20^ The effects of the intervention on the primary outcome of adverse birth outcomes and prenatal care were published previously.^19,23^ In this study, we focused on the preregistered secondary outcome of attendance of a routine postpartum care visit before 12 weeks post partum. Additionally, to investigate the full context of postpartum care use, we explored a series of nonpreregistered outcomes related to use of care in the postpartum period within 12 weeks and 1 year post partum. These included use of outpatient care (excluding the routine postpartum care visit), use of the ED without admission, and hospitalization within the first 12 weeks and within the first 1 year post partum. We categorized ED visits (with or without admission) as emergent or nonemergent using the New York University ED Algorithm and updated algorithm patch.^24,25^ The algorithm uses the primary diagnosis code at discharge to assign to each ED visit probabilities that the visit was *emergent, not preventable*; *emergent, preventable*; *emergent, primary care treatable*; and *nonemergent*. Separate categories classify visits for injury-, mental health–, alcohol-, and drug-related diagnoses and unclassifiable. Following previous literature,^24^ we classified a visit as emergent if the sum of the probabilities of *emergent, not preventable* and *emergent, preventable* was greater than 50% and we classified the visit as nonemergent if the sum of the probabilities of *nonemergent* and *emergent, primary care treatable* was greater than 50%. eTable 1 in Supplement 2 provides details of the algorithm.

### Sample Size

The trial was powered to detect differences in adverse birth outcomes and was designed to enroll 6000 participants (2000 in the control group and 4000 in the treatment group).^20^ Because of the COVID-19 pandemic, we stopped enrollment on March 17, 2020, with 5670 participants enrolled. Among randomized participants, 82% completed their 12-week postpartum period before the pandemic began.

### Statistical Analysis

Analysis was performed between February 2, 2023, and July 16, 2024. We used an intent-to-treat approach and ordinary least squares linear regression models to compare outcomes between the intervention group and control group. We estimated unadjusted models and models adjusted for baseline covariates, as described in our preanalysis plan^20^ and trial protocol in Supplement 1. All tests were 2-sided, and statistical significance was defined as *P* ≤ .05. We used dummy variables to account for missing baseline covariates in regression models and conducted sensitivity analyses using multiple imputation.^19,26^ Outcomes had no missing values, as they were defined using claims data. We also conducted sensitivity analyses using generalized linear models with binomial distribution and logit link function.^27^ Analyses were conducted using Stata software version 14.2 (StataCorp).

We explored heterogeneity of treatment effects in 3 different groups of participants who may have experienced more health challenges in the postpartum period: those who gave birth via cesarean delivery, those with chronic disease during pregnancy (including asthma, hypertension, mood or anxiety disorders, or diabetes), and those who experienced pregnancy or delivery complications (a complete list of included conditions is provided in eTable 2 in Supplement 2). We also examined the subgroup of participants whose 12-week postpartum period was not affected by the COVID-19 pandemic. For comparability with other analyses, we report on treatment effects in the prespecified subgroups of participants facing social vulnerabilities and those who self-reported their race and ethnicity as non-Hispanic Black (eTable 2 in Supplement 2).

## Results

A total of 5670 study participants were enrolled between April 1, 2016, and March 17, 2020, and 4877 participants (median [IQR] age, 21 [19-25] years) were included in the analytical sample of participants that were matched to a vital record indicating a live birth and to Medicaid eligibility files, with 3261 randomized to the intervention group and 1616 randomized to usual care (eFigure in Supplement 2). Participants included 259 Hispanic individuals (5.7%); 56 non-Hispanic Asian, Indigenous, or Native Hawaiian and Pacific Islander individuals (1.2%); 2535 non-Hispanic Black individuals (55.4%); 1587 non-Hispanic White individuals (34.7%); and 141 individuals (3.1%) with more than 1 race reported (and non-Hispanic ethnicity). Outcomes were assessed through March 2022. Match rates for the treatment and control groups were not significantly different (eTable 3 in Supplement 2). We note that outcomes using Medicaid data (eg, outpatient visits) were available only for those who maintained Medicaid coverage throughout the entire postpartum period; 47.7% of the treatment group and 48.4% of control had continuous Medicaid coverage through 365 days post partum (*P* = .62). Among participants whose postpartum period overlapped with the COVID-19 pandemic (19.0% in intervention group and 18.3% in usual care; *P* = .56), Medicaid coverage was retained beyond the usual 60 days due to the COVID-19 continuous enrollment policy (eTable 3 in Supplement 2).

### Trial Population

Characteristics of participants in the analytical sample were balanced at baseline (Table 1). In the treatment group, compared with the control group, 2739 participants (85.0%) vs 1340 participants (84.0%) received at least 1 antenatal care visit before enrollment, and 1791 participants (54.9%) vs 892 participants (55.2%) were aged 19 to 24 years (Table 1). At enrollment, 2130 participants (66.2%) in the intervention group and 1051 participants (65.9%) in the control group reported high stress; 625 intervention group participants (19.3%) and 303 control group participants (18.8%) reported depressive symptoms. In the 6 months prior to enrollment, 1675 intervention group participants (51.4%) and 843 control group participants (52.3%) used the ED. In terms of health behaviors in the 3 months before pregnancy, 1647 participants (50.8%) in the intervention group and 822 participants (51.2%) in the control group reported drinking alcohol, while 809 intervention group participants (25.2%) and 433 control group participants (27.1%) reported smoking cigarettes. The extent of missing information in baseline characteristics is shown in eTable 4 in Supplement 2.

**Table 1.** Characteristics of the Analytical Sample at Baseline

Characteristic	Participants, No. (%)^a^		
	Total (N = 4877)	Group	
		Nurse home visiting (n = 3261)	Usual care (n = 1616)
Gestational age at enrollment, median (IQR), wk	14.7 (9.0-20.0)	14.0 (10.0-20.0)	13.0 (9.0-19.0)
Received ≥1 antenatal care visit before time of survey	4079/4820 (84.6)	2739/3224 (85.0)	1340/1596 (84.0)
Age, y			
15-18	881/4877 (18.1)	594/3261 (18.2)	287/1616 (17.8)
19-24	2683/4877 (55.0)	1791/3261 (54.9)	892/1616 (55.2)
25-34	1191/4877 (24.4)	784/3261 (24.0)	407/1616 (25.2)
≥35	122/4877 (2.5)	92/3261 (2.8)	30/1616 (1.9)
Race and ethnicity			
Asian, American Indian or Alaska Native, Native Hawaiian or Other Pacific Islander, non-Hispanic	56/4578 (1.2)	44/3074 (1.4)	12/1504 (0.8)
Hispanic	259/4578 (5.7)	166/3074 (5.4)	93/1504 (6.2)
Non-Hispanic Black	2535/4578 (55.4)	1697/3074 (55.2)	838/1504 (55.7)
Non-Hispanic White	1587/4578 (34.7)	1071/3074 (34.8)	516/1504 (34.3)
>1 Race reported, non-Hispanic	141/4578 (3.1)	96/3074 (3.1)	45/1504 (3.0)
Highest education level			
<High school diploma	1087/4857 (22.4)	734/3247 (22.6)	353/1610 (21.9)
High school diploma or equivalent	1730/4857 (35.6)	1174/3247 (36.2)	556/1610 (34.5)
Some college, <bachelor’s degree	1679/4857 (34.6)	1101/3247 (33.9)	578/1610 (35.9)
≥Bachelor’s degree	360/4857 (7.4)	237/3247 (7.3)	123/1610 (7.6)
Economic conditions			
Working for pay	2575/4875 (52.8)	1728/3260 (53.0)	847/1615 (52.4)
Receiving ≥1 social service programs^b^	3164/4776 (66.2)	2105/3197 (65.8)	1059/1579 (67.1)
Housing insecurity^c^	825/4868 (16.9)	542/3256 (16.6)	283/1612 (17.6)
Currently lives with parents	2139/4873 (43.9)	1418/3259 (43.5)	721/1614 (44.7)
Mental health			
Depressive symptoms ^d^	928/4846 (19.1)	625/3236 (19.3)	303/1610 (18.8)
High stress^e^	3181/4811 (66.1)	2130/3217 (66.2)	1051/1594 (65.9)
Received mental health treatment in last year	667/4869 (13.7)	450/3258 (13.8)	217/1611 (13.5)
BMI			
<18.5	267/4718 (5.7)	183/3164 (5.8)	84/1554 (5.4)
18.5-24.9	1788/4718 (37.9)	1173/3164 (37.1)	615/1554 (39.6)
25-29.9	1033/4718 (21.9)	698/3164 (22.1)	335/1554 (21.6)
≥30.0	1630/4718 (34.5)	1110/3164 (35.1)	520/1554 (33.5)
Health behaviors and care seeking			
Reported drinking alcohol in 3 mo before pregnancy	2469/4849 (50.9)	1647/3245 (50.8)	822/1604 (51.2)
Reported smoking cigarettes in 3 mo before pregnancy	1242/4808 (25.8)	809/3212 (25.2)	433/1596 (27.1)
Health self-reported as fair or poor	597/4854 (12.3)	418/3244 (12.9)	179/1610 (11.1)
Used hospital emergency department in past 6 mo	1675/3261 (51.7)	1675/3261 (51.4)	843/1613 (52.3)
Family planning			
Has access to a place for family planning or birth control	2722/4867 (55.9)	1826/3255 (56.1)	896/1612 (55.6)
Reported a desire for more children in the future	3293/4876 (67.5)	2198/3261 (67.4)	1095/1615 (67.8)
Reported interacting with the father of the child daily	3881/4856 (79.9)	2590/3247 (79.8)	1291/1609 (80.2)

Abbreviation: BMI, body mass index (calculated as weight in kilograms divided by height in meters squared).

^a^ Percentage of missing values is shown in eTable 4 in Supplement 2.

^b^ Social services included Temporary Assistance for Needy Families, Supplemental Nutrition Assistance Program, Supplemental Security Income, unemployment benefits, and Special Supplemental Nutrition Program for Women Infants and Children.

^c^ Housing insecurity was defined by moving 2 or more times in the previous 12 months.

^d^ Defined as Patient Health Questionnaire–2 score of 3 or higher.

^e^ Defined as Perceived Stress Scale–4 score of 4 or higher.

### Program Participation

Among intervention participants, 2480 (76.1%) had at least 1 home visit in the first 12 weeks after delivery (Table 2). The mean (SD) number of visits was 4.6 (3.5) within the first 12 weeks post partum and 12.9 (11.0) within 1 year post partum. Most intervention participants had at least 1 in-person visit (2187 participants [67.1%]), while 906 participants (27.8%) had at least 1 telehealth (or virtual) visit in the first 12 weeks. Among 2664 participants unaffected by the COVID-19 pandemic, 2022 participants (75.9%) completed an in-person encounter and 471 participants (17.7%) completed a telehealth encounter in the first 12 weeks post partum, while those affected by the pandemic (597 participants) were more likely to have completed a telehealth encounter (435 participants [72.9%]) and less likely to have an in-person encounter (165 participants [27.6%]) (eTable 5 in Supplement 2). Most intervention participants (2222 participants [68.1%) were still receiving home visits at 12 weeks post partum, and more than half were still receiving visits at 1 year post partum (1712 participants [52.5%]). The rate of participation in Medicaid home visits was low: 202 participants (6.2%) in the intervention group and 182 participants (11.3%) in the control group received such a visit in the first 12 weeks post partum (eTable 6 in Supplement 2).

**Table 2.** Participation in Home Visiting Intervention: Nurse Home Visiting Intervention Group Only

Outcome	Postpartum period	
	12 wk	1 y
≥1 Completed home visit, No./total No. (%)	2480/3261 (76.1)	2497/3261 (76.6)
≥1 Completed in-person home visit, No./total No. (%)	2187/3261 (67.1)	2242/3261 (68.8)
≥1 Completed telehealth home visit, No./total No. (%)	906/3261 (27.8)	1442/3261 (44.2)
Home visits continued to within 12 wk post partum, No./total No. (%)^a^	2222/3261 (68.1)	NA
Home visits continued to within 1 y post partum, No. (%)^a^	NA	1712/3261 (52.5)
Completed home visits		
Mean (SD), No.	4.6 (3.5)	12.9 (11.0)
Participants with data, No.	3261	3261
Completed in-person home visits		
Mean (SD), No.	3.6 (3.2)	9.0 (9.0)
Participants with data, No.	3261	3261
Completed telehealth home visits		
Mean (SD), No.	1 (2.3)	4 (7.2)
Participants with data, No.	3261	3261
Home visit duration		
Mean (SD), min^b^	62 (30.4)	58 (26.3)
Participants with data, No.	2480	2497
In-person home visit duration		
Mean (SD), min^b^	69.2 (32.1)	66.7 (25.8)
Participants with data, No.	2187	2241
Telehealth home visit duration		
Mean (SD), min^b^	28.9 (15.9)	29.6 (14.9)
Participants with data, No.	906	1442

^a^ Defined as having home visit within 14 days of indicated postpartum period.

^b^ Program metrics on duration of visit are reported for participants with at least 1 home visit in the specified postpartum period.

### Program Impact

Table 3 shows the effect of the intervention on use of care in the first 12 weeks post partum. There was no significant difference in attendance of a routine postpartum visit at 12 weeks between intervention and control groups (2079 participants [63.8%] vs 1038 participants [64.2%]; adjusted difference [AD] −0.3 [95% CI, −3.2 to 2.5] percentage points). There was also no difference in use of outpatient care or inpatient hospitalization in the first 12 weeks post partum (Table 3). Intervention group participants had a significantly lower likelihood of visiting the ED without admission compared with control group participants in the first 12 weeks post partum (601 participants [18.4%] vs 333 participants [20.6%]; AD, −2.5 [95% CI, −4.8 to −0.1] percentage points). Results for nonemergent ED use were not statistically significant (317 participants [9.7%] vs 186 participants [11.5%]; AD, −1.8 [95% CI, −3.6 to 0.01] percentage points). There was no difference in likelihood of emergent ED use. These results were consistent in the group of participants unaffected by COVID-19 (eTable 7 in Supplement 2).

**Table 3.** Effects of Nurse Home Visiting Intervention on Use of Routine and Emergency Postpartum Care in the First 12 Weeks Post Partum

Care use	Group, No./total No. (%)		Between-group difference, percentage points (95% CI)	
	Nurse home visiting	Usual care	Unadjusted	Adjusted
Routine postpartum visit	2079/3261 (63.8)	1038/1616 (64.2)	−0.5 (−3.3 to 2.4)	−0.3 (−3.2 to 2.5)
Outpatient visit^a^	94/3261 (2.9)	42/1616 (2.6)	0.3 (−0.7 to 1.3)	0.3 (−0.6 to 1.3)
Hospitalization	120/3261 (3.7)	50/1616 (3.1)	0.6 (−0.5 to 1.7)	0.5 (−0.6 to 1.6)
ED visit without admission	601/3261 (18.4)	333/1616 (20.6)	−2.2 (−4.5 to 0.2)^b^	−2.5 (−4.8 to −0.1)^c^
Emergent ED visit^d^	92/3261 (2.8)	44/1616 (2.7)	0.1 (−0.9 to 1.1)	−0.02 (−1.0 to 1.0)
Nonemergent ED visit^d^	317/3261 (9.7)	186/1616 (11.5)	−1.8 (−3.6 to 0.02)^b^	−1.8 (−3.6 to 0.01)^b^

Abbreviation: ED, emergency department.

^a^ Exclusive of routine postpartum visits.

^b^ *P* < .10.

^c^ *P* < .05

^d^ Emergent and nonemergent defined via New York University ED Algorithm (eTable 1 in Supplement 2).

In the extended period of 1 year post partum, we found no difference between intervention and control groups in use of inpatient or outpatient care, nor in emergent or nonemergent ED use (Table 4). The Figure shows treatment effects by subgroup. In the control group, attendance rates for the 12-week routine postpartum visit were similar across all subgroups. Rates of outpatient care use in the first year were also similar across subgroups, including participants who may have experienced more health challenges in the postpartum period. Among participants with cesarean delivery, the nurse home visiting group had a higher likelihood of outpatient visits in the first year post partum (122 participants [12.1%] vs 47 participants [9.0%]; AD, 3.4 [95% CI, 0.1 to 6.7]). There was no other evidence of treatment effect heterogeneity across any of the subgroups who may have experienced more health challenges in the postpartum period (Figure). Treatment effects for the subgroup of non-Hispanic Black individuals and those facing social vulnerabilities were also similar to the full sample, with more limited precision (eTable 7 in Supplement 2). Sensitivity analyses showed similar results for analytical models using multiple imputation to address missingness in baseline covariates and analyses applying generalized linear models (eTables 8-10 in Supplement 2).

**Table 4.** Effects of Nurse Home Visiting Intervention on Use of Routine and Emergency Postpartum Care in the First 1 Year Post Partum

Care use	Group, No. (%)		Between-group difference, percentage points (95% CI)	
	Nurse home visiting	Usual care	Unadjusted	Adjusted
Outpatient visit^a^	353/3261 (10.8)	164/1616 (10.1)	0.7 (−1.2 to 2.5)	1 (−0.8 to 2.8)
Hospitalization	243/3261 (7.5)	107/1616 (6.6)	0.8 (−0.7 to 2.4)	0.8 (−0.7 to 2.4)
ED visit without admission	1578/3261 (48.4)	799/1616 (49.4)	−1.1 (−4 to 1.9)	−1.3 (−4.1 to 1.5)
Emergent ED visit^b^	289/3261 (8.9)	143/1616 (8.8)	0.0 (−1.7 to 1.7)	−0.1 (−1.8 to 1.6)
Nonemergent ED visit^b^	1140/3261 (35)	577/1616 (35.7)	−0.7 (−3.6 to 2.1)	−0.9 (−3.6 to 1.9)

Abbreviation: ED, emergency department.

^a^ Outpatient visit is exclusive of routine postpartum visit.

^b^ Emergent and nonemergent defined via New York University ED Algorithm (eTable 1 in Supplement 2).

**Figure.**
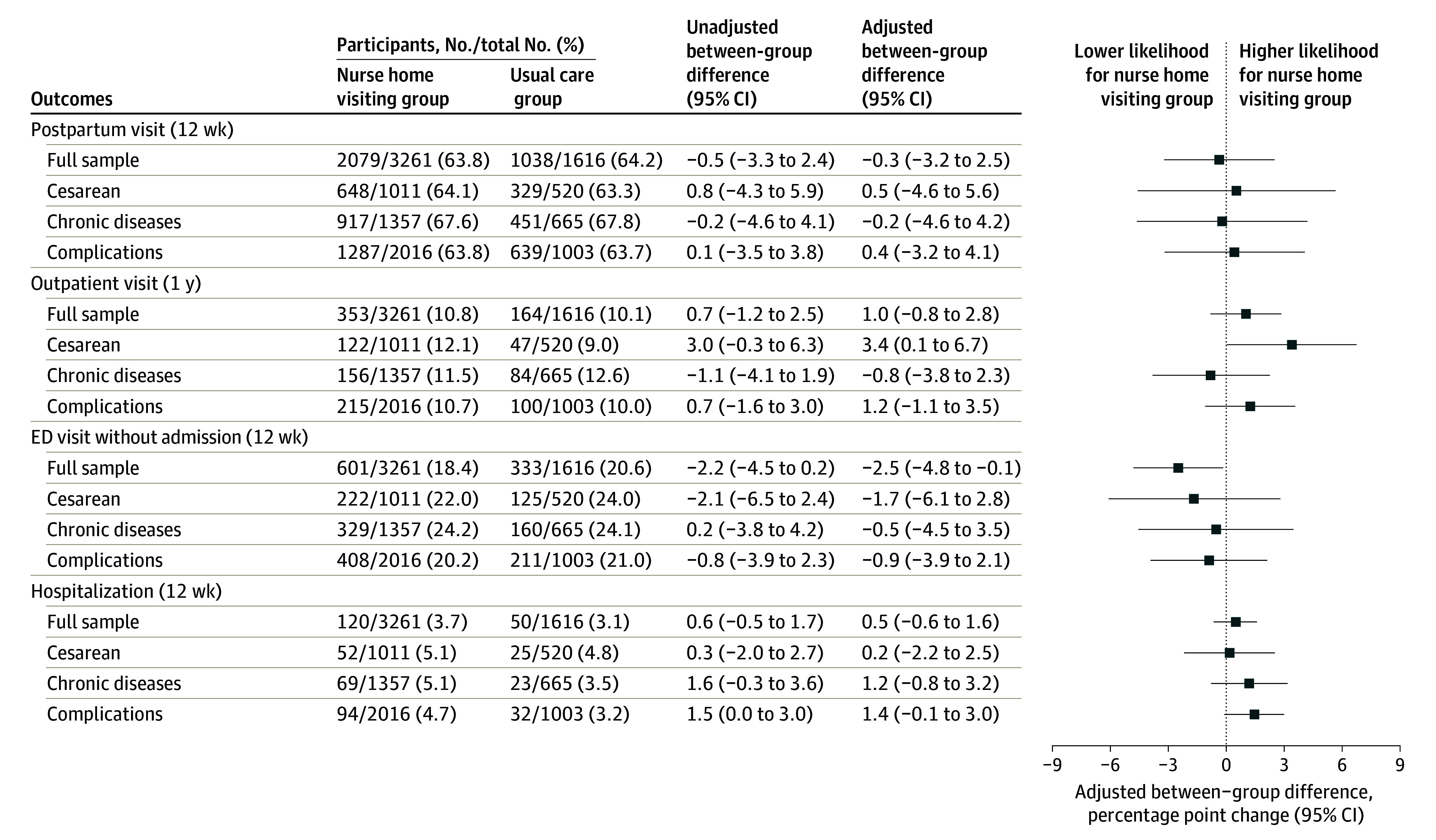
Effects of Nurse Home Visiting Intervention for Full Sample and by Subgroup ED indicates emergency department. Outpatient visits are exclusive of routine postpartum visits.

## Discussion

In this secondary analysis of a randomized clinical trial in a population of lower-income pregnant people, we did not find evidence of an impact of a nurse home visiting program on use of routine postpartum care in either the immediate (12-week) or extended (1-year) postpartum period, overall or in any high-risk subgroup. Only approximately 3 in 5 participants attended a routine postpartum visit by 12 weeks, and attendance rates were similar for those with more significant medical complexity, such as those experiencing cesarean delivery, chronic disease, and delivery complications. These results are in line with a 2022 systematic review that found that a mean of 64% of Medicaid recipients across studies attended their postpartum visit.^10^ Moreover, effects for the subgroup of non-Hispanic Black individuals were similar to those for the full sample, with more limited precision. Therefore, our trial does not provide evidence for the hypothesis that access to home visits can address racial disparities in postpartum outcomes.

Postpartum care can be an important way to improve the physical and emotional health and well-being of pregnant people.^13,28^ Many people struggle with numerous new health problems in the early postpartum period, including hormonal and physical changes, recovery from delivery, sleep deprivation, and stress.^29^ High-quality postpartum care can aid in detection of cardiac or hypertensive conditions or mental health challenges that can have implications for a person’s later-life health trajectory.^30,31,32^ Coordinated care to transition pregnant people from obstetrical to primary care is needed to address immediate postpartum health care needs, develop disease management plans, and promote a health care foundation for future care needs. Because nurses have regular encounters with their clients in the postpartum period, home visiting could potentially strengthen continuity of care for lower-income individuals. However, while nurses can improve postpartum education and assist with social determinants that impede access to care, structural challenges that nurses are not able to address, such as Medicaid eligibility policies, shortage of primary care practitioners, and lack of collaboration between obstetric and primary care practitioners, which may be significant barriers to improving transition of care.^5,33^

In exploratory analysis, we found that the intervention led to a small but significant reduction in ED visits without admission in the first 12 weeks post partum, while there were no differences in other outcomes, such as hospitalizations and outpatient visits. Consistent with previous literature,^34,35,36^ use of the ED in the first 12 weeks post partum was common in the study population. Reducing ED visits has positive implications for lowering health care costs^37,38^ and improving the appropriateness of care delivery during the postpartum period. Home visiting may have reduced unnecessary ED use through nurses improving clients’ health literacy, providing education about what to expect in the postpartum period, and triaging health concerns during home visits. More research to further explore these pathways is needed.

Our study has implications for ongoing discussions about how to improve perinatal care for low-income populations. Use of outpatient care in the 12-month postpartum period was low (approximately 10%); one reason for this may have been that less than half of participants retained Medicaid coverage beyond 60 days post partum. Just under 20% of participants retained Medicaid coverage beyond the usual 60 days due to the COVID-19 pandemic’s continuous enrollment condition. Since the pandemic, 47 states have implemented extension of postpartum Medicaid coverage from 60 days to 1 year.^39^ Evaluating the impact of this extension on use of postpartum health services and outcomes is a research priority.^40^ Finally, our results showing lack of impact on routine postpartum care use are consistent with the results we previously published that found no impact of nurse home visiting on the intensity or quality of prenatal care services.^23^ Together, results suggest that nurse home visiting may not be an efficient strategy to increase use of perinatal health care services among low-income populations. Overall, more than 20 states have implemented Medicaid reimbursement for home visiting services,^41^ and some have expanded coverage for doulas and community health workers for postpartum people and their families.^42^ Our results suggest that more research is needed to better understand effective strategies using the growing perinatal health workforce in supporting engagement with perinatal care.

### Limitations

This analysis has limitations. First, as not all outcomes were prespecified end points in the trial protocol, our analysis is exploratory. Furthermore, as we consider multiple outcomes in multiple subgroups, it would be valuable to confirm these exploratory results in future research. In this report, we focused exclusively on postpartum care use. Other postpartum outcomes, such as uptake of family planning, mental health, and birth spacing, are outside the scope of this report and will be explored in future analyses. Second, some control group participants received a Medicaid home visit, which they were entitled to under South Carolina’s Medicaid program. However, this rate was relatively low (11%) compared with the number and intensity of home visits in the NFP group. It is possible that control group participants also participated in other community programs, which may have dampened potential treatment effects. However, these programs operated at a substantially smaller scale compared with NFP during the trial. Third, we used administrative data, including discharge records and Medicaid claims, to identify postpartum care use, which may not capture all sources of postpartum care. In particular, participants who lose Medicaid eligibility or who switch to another form of insurance in the postpartum period would not appear in Medicaid claims. However, we found that the rate of continuous Medicaid coverage in the first year post partum was not different across treatment and control groups. Moreover, our estimate of postpartum visit attendance was very similar to a 2022 systematic review.^10^ Fourth, administrative data were missing for participants who were not successfully matched to a live birth at delivery, although the rate of matching to the analytical sample was similar in intervention and control groups. Fifth, our study enrollment and intervention were affected by the COVID-19 pandemic. Nurse encounters were largely converted to telehealth without a change in the overall number of encounters. Among the subgroup of individuals whose postpartum periods were not affected by the pandemic (82%), treatment effects were consistent with the main results.

## Conclusions

This secondary analysis of a randomized clinical trial did not find that nurse home visiting increased use of routine postpartum or outpatient care in the immediate or extended postpartum periods, even among those with greater health challenges, which may reflect a missed opportunity. However, nurse home visiting reduced ED use, which has positive implications for reducing health care costs and promoting care access in appropriate settings. Improving continuity of care for patients with low income before, during, and after pregnancy is an important policy priority for addressing maternal health inequities.^5^ The postpartum period (sometimes known as the *fourth trimester*) is an important opportunity to address immediate health risks, as well as establish care plans for longer-term management of chronic disease.^30^ This is particularly important for the Medicaid population, who have high rates of chronic health conditions and mental health challenges.^43^ More research is needed on how to improve continuity of perinatal care for low-income populations.

## Notes

Supplement 1.
Trial Protocol and Statistical Analysis Plan

Supplement 2. **eFigure 1.** Participant Recruitment Flowchart **eTable 1.** Sample and Variable Algorithms **eTable 2.** Subgroup Definitions **eTable 3.** Match Rates Between Treatment And Control **eTable 4.** Percentage of Missing Values on Self-Reported Baseline Characteristics **eTable 5.** Program Participation Stratified by Whether Postpartum Period Was Affected by COVID-19 **eTable 6.** Participation in Medicaid Home Visits at 12 Weeks Post Partum **eTable 7.** Subgroup Treatment Effect Estimates **eTable 8.** Multiple Imputation Results **eTable 9.** Logistic Regression Results: Full Sample **eTable 10.** Logistic Regression Results: Subgroups

Supplement 3.
Data Sharing Statement
