# Supplementary material for: Home Visits and the Use of Routine and Emergency Postpartum Care Among Low-Income People: A Secondary Analysis of a Randomized Clinical Trial
Source: JAMA Netw Open. 2024 Dec 23;7(12):e2451605. doi: 10.1001/jamanetworkopen.2024.51605 (PMC11667346; doi:10.1001/jamanetworkopen.2024.51605)
Supplement: Supplement 1. — Trial Protocol and Statistical Analysis Plan [file jamanetwopen-e2451605-s001.pdf]

RESEARCH PROTOCOL AND STATISTICAL ANALYSIS PLAN

Original IRB protocol with all amendments ……………………………………………….……. begins on page 2
Statistical analysis plan (latest version) …………………………………………………………. begins on page 33

Instructions: The purpose of this research protocol is to provide IRB members and reviewers with sufficient information to conduct a substantive review. If a separate sponsor's protocol exists, please submit it in addition to this document.

Complete all of the sections below. For more detailed instructions, please consult the Investigator's Manual or IRB website (links provided below).

GENERAL INFORMATION	
**Protocol #** (if assigned): IRB15-2939	
**Version Date:** 07/21/2021 (originally approved 12/9/2015)	**Version Number:** 20
**Principal Investigator:** Margaret McConnell, PhD	
**Faculty Advisor (**if PI is a student**):**	
**Protocol Title:** Randomized Evaluation of Nurse Family Partnership in South Carolina	

1. Specific Aims

Objective

To evaluate the Nurse Family Partnership (NFP), an existing home-visiting program in South Carolina, using a scientifically rigorous individual-level randomized controlled trial. The evaluation will be conducted in partnership with the NFP program and the South Carolina Department of Health and Human Services. Additional statewide partners include: The Duke Endowment, South Carolina First Steps to School Readiness, BlueCross BlueShield of South Carolina Foundation, Children's Trust of South Carolina and the South Carolina Department of Health and Environmental Control (DHEC).

Research questions

What is the average impact of NFP on 1) pre-term birth, 2) birth spacing, 3) child injury, and 4) long-term health, education, and economic self sufficiency of the family? What proportion of study participants in the treatment group reside in low-income zip codes (LIZCs) at the time of study enrollment?

2. Background

2.1 Provide the scientific background and rationale for the study

Prior Evidence

Nurse Family Partnership (NFP) is a long-standing prenatal and infancy home visiting program for low-income, first-time mothers and their families. Registered nurses begin visiting their clients as early in the pregnancy as possible, helping the mother-to-be make informed choices for herself and her baby. The nurses continue visiting the mothers after their baby is born; meeting weekly or bi-weekly, until the child reaches two years of age.

From 1978 to 1994, three modest-scale randomized evaluations were conducted in Elmira, NY, Memphis, TN, and Denver, CO to estimate the impact of NFP on first-time, low-income mothers and their children. Follow-up research on these three study samples continues today, to examine the impact of NFP on long-term outcomes for mothers and children. Across the three trials, evidence suggests that NFP improves pre-natal health, reduces childhood injuries, increases the spacing between subsequent pregnancies, increases maternal employment, and improves school readiness for the children of mothers who participated in the program.

However, there are a number of reasons that the impact of NFP in South Carolina today may differ from the impacts detected in previous trials. First, the socio-economic composition of the mothers and children may differ from those in the previous study samples. Second, the standard of care that all Medicaid-eligible mothers and children receive now may be better than that in the previous trials. Third, many of the long-term outcomes in previous trials were based on self-reported survey data, which may be less accurate than the administrative data on which we will rely for both short- and long-term outcomes.

Unmet Need
South Carolina ranks 45th in the country for child well-being, according to the Annie E. Casey Foundation's analysis of data on health, education, economic well-being, and family and community.[i] In particular, the need for home-visiting services in South Carolina currently exceeds the capacity of local programs. For example, NFP currently serves only about 800 of 11,500 eligible high-risk mothers each year.

To improve maternal and child health outcomes, the South Carolina Department of Health and Human Services (SCDHHS) is utilizing a Medicaid waiver to help cover the costs of NFP home visits and establishing a Pay-for-Success (PFS) contract to expand the Nurse Family Partnership home-visiting program statewide, with a focus on underserved rural areas. This expansion will not be able to cover all eligible women, however. Given, that the program has limited capacity, a lottery was seen as a fair way to allocate slots in the program. This provides a valuable opportunity to learn about the effects of the NFP program on mothers and children through a randomized controlled trial, and indeed the expansion is predicated on just such an evaluation being conducted.

2.2 Describe the significance of the research, and how it will add to existing knowledge
President Obama's Budget requests $500 million for fiscal year 2016 and $15 billion over the next 10 years to expand Home Visiting for families.[ii] This research is important because it will strengthen the evidence base for home-visiting programs, help government officials and philanthropists assess the value of programs like NFP, and inform future decisions about funding and expanding such programs. Part of the state and federal government's willingness to fund the expansion of the NFP program is contingent on an evaluation of its effects. This research will thus not only add to our understanding of how the NFP program impacts mothers and children when NFP is brought to scale in the current economic and health care environment, but can also help inform the allocation of billions of dollars meant to improve health and well-being in fiscally sustainable programs.

3. Study Setting

3.1. Identify the sites or locations where the research will be conducted.
NFP is currently implemented through nine health care centers in South Carolina, and all nine will participate in the study:

- Department of Health and Environmental Control (four sites: Upstate, Pee Dee, Lowcountry and Midlands)
- McLeod Health System

[i] KIDS COUNT Databook, 2013.
[ii] http://www.hhs.gov/news/press/2015pres/02/20150219a.html

- Greenville Health System
- Spartanburg Regional Health System
- Carolina Health Centers
- Family Solutions of the Lowcountry

NFP program staff, trained in human subjects' protections and the study protocol, will conduct study enrollment (including informed consent, baseline survey administration, and randomization). They will enroll mothers in the study in-person, in a private setting that is convenient and comfortable for the mother. These locations may include a private room at a clinic, the study participant's home, or another neutral, private location of the mother's choice. Due to COVID-19, nurses will enroll mothers in the study virtually. NFP is set up to provide home visiting services via Telehealth and has provided additional guidance to nurses about how to ensure similar service delivery with Telehealth which includes: planning logistics and expectations of a telehealth encounter, discussing how silence may be difficult to interpret and encourage both parties to discuss any concerns, and keeping in mind and discussing safety concerns and plan signals that can be made during a telehealth encounter if the client needs to end the call with non-verbal communication.

3.2. Describe the Principal Investigator's experience conducting research at study site(s) and familiarity with local culture

Dr. Margaret McConnell's research combines behavioral economics with field and laboratory experiments to understand and evaluate policies designed to change health behaviors, with a specific focus on maternal and child health. Her work among low-income populations in Boston examines the impact of cash assistance on the amount of time that families spend engaging in Kangaroo Mother Care with babies born prematurely. Her ongoing research in Kenya examines the effect of cash transfers incorporating pre-commitment on the choice of a high quality maternal delivery facility and the impact of vouchers with and without deadlines on the uptake of postpartum family planning. Her work focuses largely on urban areas with poor populations.

3.3. Is the research conducted outside the United States?

☒**No** ☐**Yes: If yes; describe site-specific regulations or customs affecting the research, local scientific and ethical review structure**

3.4 Are there any permissions that have been or will be obtained from cooperating institutions, community leaders, or individuals, including approval of an IRB or research ethics committee? ☐**No** ☒**Yes: If yes; provide a list of the permissions (also include copies with the application, if available)**

This project is being conducted in cooperation with the state of South Carolina's Department of Health and Human Services as well as the NFP program. Statewide partners include: The Duke Endowment, South Carolina First Steps to School Readiness, BlueCross BlueShield of South Carolina Foundation, Children's Trust of South Carolina and the South Carolina Department of Health and Environmental Control (DHEC). Expansion of the NFP program is predicated on an evaluation of its effects, which we will be conducting in cooperation with these public and private partners.

The following IRBs have been invited to rely on Harvard's IRB for review, approval, and continuing oversight of this research study:

<u>**Research team:**</u>

- MIT (Co-Investigator's affiliation)

Health Care Centers:

- South Carolina Department of Health and Environmental Control (includes sites at Upstate, Pee Dee, Lowcountry and Midlands)
- McLeod Health System
- Greenville Health System
- Spartanburg Regional Health System

Two health care centers, Carolina Health Centers & Family Solutions of the Lowcounty, do not have institutional review boards. Each staff person at these agencies who will conduct study enrollment will first complete the CITI Training and sign an Individual Investigator's Agreement before the research starts.

If the institutions listed above do not agree to rely on Harvard's IRB, we will cooperate with their review boards to obtain approval. We will submit any external permissions to the Harvard IRB.

We have also requested approval for this research project from NFP's National Service Office. A copy of their approval is attached.

4. Study Design

4.1. Describe the study design type

This study is an individual-level randomized controlled trial where all eligible applicants who provide written consent will be randomly assigned either to a treatment group that is offered access to NFP or to a control group that is not offered the opportunity to enroll in NFP. Two-thirds of those recruited will be randomized to the treatment group and one-third to the control group. Control group members will have access to the standard of care and whatever other programs and services are available in the community.

Prior to randomization, eligible applicants who provide written consent will be asked to complete a brief 30 minute baseline interview. Using encrypted tablets and Computer Assisted Personal Interview (CAPI) software, the NFP program staff will ask the participant questions about her health, feelings, use of social services, and what she hopes to get from the home visiting program. For completing the baseline interview, each participant will receive a $25 gift card as compensation for her time. Data collected at baseline will be used to describe the characteristics of the study sample and to assess the baseline equivalence of the treatment and control groups post-randomization.

Receiving the data file for all program participants from our partner is necessary to correctly identify study participants and match them to program data. We use this data to identify and correct data entry errors in either our survey data or our partner's program data. Disagreements between our study data and the NSO program data can take time to reconcile. This necessitates keeping the full set of NSO program for 1 year in order to ensure that all study participants have been accurately identified. For example, an individual in the NSO program data may initially appear to not be a study participant. However, due to data entry errors, it may be found that this individual is indeed a study participant after multiple iterations of data cleaning. Without keeping the full set of NSO program data for one year, we

would not be able to reconcile and ensure high quality matched data across these two sources.

After the 1 year study period, data on any individuals in the NSO program data who have not been identified as study participants will be deleted. All NSO program data is kept on a Harvard Level Four server. Access to the Server is restricted, two-factor authentication is required for all users, and compliance with Harvard's level four protocol is review quarterly.

Receiving this data is part of the contract we have signed as evaluators of this project. Specifically, the contract requires that NSO provides data on all potential clients (not just those consented to the study).

To assess the impact of offering NFP, we will collect administrative data for both treatment and control group members from various state agencies. Data for pre-term birth and birth spacing will be obtained from Vital Records, collected and maintained by the South Carolina Department of Health and Environmental Control. Data on child injuries will be obtained from an all-payer encounter database overseen by the South Carolina Data Oversight Council. These three outcomes are of primary interest to the public and private partners in formally evaluating the effects of NFP (as part of the conditions for paying for the expansion), and the relevant state offices are committed to facilitating this data collection.

We will also collect administrative data on additional outcomes such as health care utilization, mortality, education attainment, abuse and neglect, employment/earnings, use of government services, credit, and crime. This broader evaluation will provide a more comprehensive assessment of the impact of the NFP program, both on additional outcomes beyond the outcomes used for program payments and over a longer time period. This will yield useful specific evidence for the State of South Carolina, and for policy makers nationally, who are interested in comprehensive analyses on the social and economic impact of the NFP program. We will use an instrumental variable approach to measure program impacts.

4.1.1 Nurse survey

To support our understanding of the findings from this RCT, we will also administer a survey to all nursing staff who have delivered services throughout the time period of study enrollment. The purpose of this survey is to understand the implementation of home visiting services during the study period and to explore heterogeneity in impact based on nurses' and implementing agencies' characteristics.

Prior to completing the survey, nurses will be asked to provide consent. After providing consent, nurses will be asked to complete a 30 minute survey. Nurses will be asked a series of questions about their demographics, prior employment and educational history, as well as about their current experiences working at the Nurse Family Partnership in South Carolina. Nurses who complete the survey will receive a $25 gift card. Data collected will be used to describe the characteristics of nurses and agencies that are implementing the home visiting services that are being studied. This information will also be matched to the NSO programmatic records to understand heterogeneity in program effects.

4.2. Indicate the study's duration - and the estimated date of study completion

Study enrollment will take place over a four year period between January, 2016 and July, 2020. Each mother and child will be enrolled in the NFP program until the child is 2 years old, at which point their interaction with the program and the study will end. The initial impact will be estimated using administrative data around January, 2023. The long-term outcomes will be gathered from administrative data for up to thirty years, but will not involve direct contact with the mothers or children.

4.2.1 Duration of the nurse survey

The survey will be administered over a 8 month period between March 2020 and October 2020. This information will be stored and matched with administrative data for up to thirty years, but will not involve any additional follow-up with the nurses.

4.3. Indicate the total number of participants (if applicable, distinguish between the number of participants who are expected to be screened and enrolled, and the number of enrolled participants needed)

Over the four year period, NFP expects to screen 26,000 applicants for eligibility and enroll 6,000 of those into the study; approximately 4,000 will be randomly assigned to the treatment group and 2,000 to the control group.

4.3.1. Total number of participants in the nurse survey.

Over the 6 month period we plan to survey up to 200 nurses.

4.4. List inclusion criteria

**Eligibility Criteria**	**Process for assessing criteria**
Female	Applicant's self-report
No previous live births	Applicant's self-report
Currently pregnant	Applicant's self-report of having received a positive pregnancy test
Gestation period less than 28 weeks (i.e. less than or equal to 27 weeks, 6 days)	Estimated due date; best guess of practitioner and applicant
Ages 15-55	Applicant's self-reported date of birth
Income level meets Medicaid eligibility criteria	Applicant's self-report, verify enrollment and/or eligibility in QuickChecks (State eligibility determination program)
Live within an area serviced by a NFP Implementing Agency	Applicant's self-report of current mailing address
Not currently enrolled in the study	Search for matches by last name & DOB in the study database

Not incarcerated or living in lock down facilities	Applicant's self-report or as reported by the relevant agency or facility

4.4.1 List inclusion criteria for the nurse survey

- Any nurse home visitor or nurse supervisor who delivered NFP services during the study enrollment period 2016 – 2020.

4.5. List exclusion criteria

- Men
- Women who have had a previous live birth
- Women who are not currently pregnant
- Women who are past their 28th week of gestation (i.e. greater than or equal to 28 weeks, 0 days)
- Women who are younger than 15 or older than 55 years of age
- Women whose income level does not meet Medicaid eligibility criteria
- Women who live outside of an area serviced by a NFP Implementing Agency
- Women who are currently enrolled in the study
- Women who are currently incarcerated or living in a lock down facility

4.5.1 List exclusion criteria for the nurse survey

- Any nurse home visitor or nurse supervisor who has not provided any home visiting services during the study enrollment period.

4.6. Describe study procedures

NFP program staff will determine if program applicants are eligible for the study using the eligibly criteria and processes specified above. If NFP program staff determine that the applicant is *not* eligible for the study, they will provide the applicant a list of other available resources in the community, and their interaction will end there. If the applicant is eligible, the program staff will review the consent form with her and will explain verbally to her:

- what it means to be in the study
- the associated risks and benefits of participating in the study
- that being in the study is voluntary
- that she may or may not be randomly selected to participate in NFP
- that being in the study will not affect any benefits that she and her children are otherwise entitled
- that she is free to stop taking part in the study at any time
- that she can refuse to answer any questions in the interview
- that any information that can be used to identify her will be kept private, unless there is concern that she or someone else may be harmed
- that she will be asked to provide her contact information
- that the research team may obtain information from the state or federal government, from home visiting programs, and from her family's health care providers.
- that she is free to ask questions about the research now or at any time in the future

The program staff will check the applicant's understanding of what it means to be in the study, and will ask the applicant if she has any questions. Once the applicant is fully informed, the

program staff will ask the applicant to electronically sign and date the informed consent form if she agrees to participate in the study. The program staff conducting study enrollment also will sign and date the consent form electronically. If she does not agree to participate in the study, the program staff will document her refusal on a referral tracking form. In response to COVID-19, written consent will be waived. Verbal consent will be obtained until in person study visits are possible.

Each applicant who agrees to participate in the study will then be asked to complete a short, 30 minute interview about her background, relationships, health and use of social services. The applicant will be reminded that she may refuse to answer questions at any time. The program staff will verbally ask the applicant questions, and will enter the applicants' responses into an encrypted survey form. The applicant will be informed during the consent process that the interview may also be recorded for quality assurance purposes.

All eligible program applicants who provide their written consent and complete the baseline interview will be randomly assigned "on-the-spot" to a treatment group that is offered access to the NFP program or to a control group that is not offered the opportunity to enroll in NFP. Program staff will use a pre-programed randomization function on their tablets to conduct the random assignment. Program staff will explain to the participant that the computer chooses which study group she will be assigned to, and that her assignment is not dependent on any personal traits or characteristics.

If the applicant is assigned to the control group, the program staff will explain that she was not chosen by the computer to receive NFP services, but that she may continue to seek care at the health care center as she normally would. The program staff will also provide a list of other resources and programs that are available in the community.

If the applicant is assigned to the treatment group, the program staff will proceed to schedule her first home-visiting appointment. The program staff will also provide treatment group members with a list of other resources and programs that are available in the community. In response to COVID-19, the materials the applicant would have otherwise received in person (copy of the consent form, $25 gift card, and list of community resources) will be mailed to the address the applicant provides.

4.6.1. Describe study procedures for the nurse survey

Nurses will receive an email with a link to complete an online survey at a time that is convenient for them. Nurses will read the consent form and have an opportunity to email or call research staff regarding any questions before consenting to the survey. Everyone who consents to the survey will then be asked to complete the online survey.

Former NFP nurses who delivered NFP services at some point during the 2016-2020 period will receive a subset of questions to answer from the full survey.

4.7. Does the study involve the collection of data/specimens (including the use of existing data/specimens)?

☐ No ☒ Yes: If yes; indicate how, when, where and from whom specimens or data will be obtained

NFP program staff will collect identifying information (including name, date of birth, social security number, address, and Medicare and Medicaid IDs (if applicable)) and baseline data (see draft baseline survey instrument attached) from study participants at intake, prior to randomization. The identifying information will be kept separate from the baseline survey responses. A unique code, not containing any identifying information, will be used to analyze participants' baseline responses.

As explained to subjects in the Informed Consent documents, identifying information will be used to access historical and future administrative records that relate to the health and wellbeing of the mother and her children. This could include data on the birth of the mother's children, health care use (such as how many times she or her children visit a health facility), government records, interaction with government services (such as cash assistance, food stamps, or child welfare), or from her children's school after they start going to school. All of the information listed above is already collected about the mother and her children, even if they are not in the study. Taking part in the study means that the mother will allow our research team to use this existing secondary information for research purposes.

If a consented study participant is later discovered to be ineligible for the study/program (e.g. because she is over-income, not pregnant, or farther along in her pregnancy than she initially reported at study intake), the study team will continue to track and analyze administrative data for the participant and her children.

4.7.1 Data collection for nurse survey

For the nurse survey, the research team will collect nurse name. Nurse name will be collected and nurse responses will be merged into the administrative datasets. The identifying information will be kept separate from the survey responses. A unique code, not containing any identifying information, will be used to analyze nurses' responses.

The Evaluation team has executed data use agreements with the following agencies:

- **BAA with Nurse Family Partnership**, **for implementation data**. These data will be used to describe the implementation of Nurse Family Partnership in South Carolina during the study period, its adherence to program fidelity standards, the dosage received by treatment group participants and the exposure of control group members to NFP services.
- **BAA with South Carolina Department of Health and Human Services, for individually identified health data.** These data will be used to measure the effects of NFP on maternal and child health outcomes.
- **BAA with South Carolina Revenue and Fiscal Affairs Office, for individually identified health data.** These data will be used to measure the effects of NFP on maternal and child health outcomes.
- **DUA with South Carolina Revenue and Fiscal Affairs Office, for Public Use Encounter Data.** These data include encounter level Inpatient, Outpatient Surgery, Emergency Department, and Imaging records from 2010-2014. Analysis will focus on women with at least one childbirth (from diagnosis and procedure codes) whose primary payer is Medicaid and children whose primary payer is Medicaid, but records for other individuals will be used to conduct comparison analyses. These data will be used to design the analysis plan for this study.

- **DUA with South Carolina Revenue and Fiscal Affairs Office, for Encounter Data with Restricted Elements.** Records (any inpatient, outpatient surgery, emergency department discharge records or imaging records) will be matched to a master list of study subjects using First Name, Middle Name, Last Name, Social Security Number, and Date of Birth. These data will be used to measure the effects of NFP on child injury.
- **DUA with South Carolina Department of Health and Environmental Control, for historical birth certificate data.** Historical Vital Statistics data will aid the understanding of the study population. Our research team will use the data to conduct power calculations for various outcomes, to explore the predictive strength of potential control variables, and to understand determinants of maternal and child health in the Medicaid population. These data will be used to design the analysis plan for this study.
- **DUA with South Carolina Department of Health and Environmental Control, for matched birth certificate data, including child's name and date of birth.** These data will be used to measure the impact of NFP on pre-term birth and birth spacing for study participants.
- **DUA with The University of South Carolina, for matched data on MIECHV-funded home visiting programs.** These data will be used to assess the proportion of study participants who receive other home-visiting services funded by the Maternal, Infant, and Early Childhood Home Visiting (MIECHV) program.
- **DUA with First Steps, for matched data on study participants' participation in home-visiting programs affiliated with First Steps.** These data will be used to assess the proportion of study participants who receive other home-visiting services.
- **DUA with South Carolina State Law Enforcement Division, for matched data on arrests, custody, offense, and judicial outcomes.** Records will be matched to a master list of study subjects using First Name, Middle Name, Last Name, Social Security Number, and Date of Birth. These data will be used to measure the effects of NFP on criminal behavior.
- **DUA with South Carolina State Law Enforcement Division, for historical and current de-identified data for the entire state of South Carolina on arrests, custody, offense, and judicial outcomes.** These data will be used to improve our understanding of the study population. It will be used to conduct power calculations for various outcomes, to explore the predictive strength of potential control variables, and to understand determinants of maternal and child mental health.
- **DUA with South Carolina Department of Mental Health, for matched data on mental health diagnoses and services.** Records will be matched to a master list of study subjects using First Name, Middle Name, Last Name, Social Security Number, and Date of Birth. These data will be used to measure the effects of NFP on mental health.
- **DUA with South Carolina Department of Mental Health, for historical and current de-identified data on mental health diagnoses and services.** These data will be used to improve our understanding of the study population. It will be used to conduct power calculations for various outcomes, to explore the predictive strength of potential control variables, and to understand determinants of maternal and child health.
- **DUA with South Carolina Department of Education, for matched data on test scores, enrollment, attendance, and disciplinary action.** Records will be matched to a master

list of study subjects using First Name, Middle Name, Last Name, Social Security Number, and Date of Birth. These data will be used to measure the effects of NFP on education.

- **DUA with South Carolina Department of Education, for historical and current de-identified data on test scores, enrollment, attendance, and disciplinary action.** These data will be used to improve our understanding of the study population. It will be used to conduct power calculation for various outcomes, to explore the predictive strength of potential control variables, and to understand determinants of maternal and child health.
- **DUA with South Carolina Department of Corrections, for matched data on incarceration.** Records will be matched to a master list of study subjects using First Name, Middle Name, Last Name, Social Security Number, and Date of Birth. These data will be used to measure the effects of NFP on incarceration.
- **DUA with South Carolina Department of Corrections, for historical and current de-identified data on incarceration.** These data will be used to improve our understanding of the study population. It will be used to conduct power calculation for various outcomes, to explore the predictive strength of potential control variables, and to understand determinants of maternal and child health.
- **DUA with South Carolina Department of Social Services, for matched data on the use of social services and human services.** Records will be matched to a master list of study subjects using First Name, Middle Name, Last Name, Social Security Number, Date of Birth, and Medicaid number. These data will be used to measure the effects of NFP on use of social services and human services.
- **DUA with South Carolina Department of Social Services, for historical and current de-identified data on the use of social services and human services.** These data will be used to improve our understanding of the study population. It will be used to conduct power calculation for various outcomes, to explore the predictive strength of potential control variables, and to understand determinants of maternal and child health.

4.8. Is there a data and safety monitoring plan (required for greater than minimal risk studies)?
☒No ☐Yes: If yes; describe the plan

4.9. Are there any anticipated circumstances under which participants will be withdrawn from the research without their consent?
☒No ☐Yes: If yes; describe the circumstances as well any associated procedures to ensure orderly termination

5. Data/Statistical Analyses Plan

5.1. Briefly describe the plan for data analysis (including the statistical method if applicable)

To answer the research questions, we will use an instrumental variable (IV) approach and administrative data to estimate the local average treatment effect of NFP on intervention group members who actually participate in NFP relative to the services consumed by the control group. This estimated effect of NFP is of policy interest because it represents the impact of NFP on those moms who are likely to participate in NFP were the program to expand and offer additional program slots through a lottery. The source of non-compliance that it explicitly

captures is that some mothers randomized into the intervention group may never receive NFP services (i.e. the “enrollment rate” is less than 1). According to the enrollment protocol, no mothers in the control group should be enrolled in NFP services. To the extent that some sample members in the control group receive services from similar home visiting programs that may also affect outcomes, this model estimates the effect of NFP relative to the mix of other home-visiting programs that the control group receives, rather than relative to no home-visiting service at all.

The impact estimate is the regression-adjusted difference between the average outcomes of treatment group participants and control group participants. Impact estimates with p-values less than 0.05 (two-tailed test) will be considered statistically significant and provide evidence that there are likely true differences between the groups as a result of NFP.

In addition to these impact estimates, we will also report the percentage of treatment group participants who reported living in a low-income zip code (LIZC) at the time of study enrollment. (LIZC is defined as a zip code in which fifteen percent (15%) or more of households have incomes below the federal poverty guidelines according to 2012 Census Bureau Data.) Before reporting this percentage, we will use Google Map’s Application Programming Interface (API) to confirm that the participant’s self-reported zip code corresponds with their self-reported street address. To do this, we will send a file of unformatted, raw street addresses, cities, and states to Google’s API to return formatted addresses, including zip code. We will then compare these formatted zip codes to the zip codes reported by each study participant. The file we send to Google Map’s API will include a greater number of ‘fake’ (i.e. non-study participant) addresses than actual addresses, to maintain study participants’ confidentiality.

We will also analyze how the main impact estimates vary within our sample. In particular some mothers and their children may be at higher risk for adverse outcomes such as preterm birth or childhood injury. We will define “risk” based on poverty rate in the census tract in which a mother is enrolled. In order to perform this analysis, we will match participants’ self-reported addresses to census tracts in two ways. First, we will send a de-identified file that will only include a randomly generated ID number and the following address information of unformatted, raw street addresses, cities, states, and zip codes to the United States Census Bureau’s Geocoder tool as well as to the geocoding service, Geocodio. We will match addresses to census tracts using both the Census Bureau Geocoder tool as well as Geocodio in order to be able to cross validate the accuracy of the census tract mapping. Mapping to the wrong census tract or failing to map to a census tract will reduce the accuracy of our analysis. The randomly generated ID number will allow for linking census tract information back to our data. Second we will check perform a last check of the accuracy of our first method of linking to census tracts by retrieving the latitude and longitude of participants’ addresses via the Google Maps API in the same procedure described above. We will then use these latitude and longitude coordinates to link addresses to census tracts using cartographic boundary files provided by the Census Bureau. Once we have matched participants’ addresses to census tracts, we will investigate how our main impacts vary by the census tract rate of poverty. The files we send to The Census Bureau’s Geocoder tool, to Geocodio, and to Google Map’s API will include a greater number of ‘fake’ (i.e. non-study participant) addresses than actual addresses, to maintain study participants’ confidentiality.

5.2. Is there a sample size/power calculation?

☐**No** ☒**Yes: If yes; describe the calculation and the scientific rationale, and, if applicable, by site and key characteristics such as participant demographics**

NFP plans to serve 4,000 families in South Carolina between January 2016 and January 2020. Our power analysis suggests that with 4,000 families served in the treatment group, we will need a 2:1 T:C ratio (or 2,000 control group families) to achieve 80% or higher chance of detecting statistically significant effects on two out of the three primary outcomes (pre-term birth, birth spacing, and childhood injury), if the true effect size of the outcomes equals the expected effect size provided by NFP. These expected effect sizes are based on analysis of previous NFP trials and baseline knowledge on the current population of South Carolina's Medicaid-eligible first-time mothers and the assumptions summarized below.

As of May, 2015, the expected effective sizes, in percentage difference between the treatment group and the control groups, is -20% for the measure of pre-term birth, -18% for the measure of birth spacing, and -13% for the measure of child injury. In addition, we need 95% or more of women randomized into the treatment group to receive NFP services, and at most 5% of the control group that receives a home visiting program that has as much impact as NFP. Furthermore, our power calculation is based on the assumption that the distribution of the outcomes variables have the following mean (standard deviation) combinations in the absence of NFP intervention: 0.12 (0.32) for the binary measure for pre-term birth, 0.14 (0.34) for the binary measure of birth spacing, and 0.18 (0.30) for the measure of child injury.

With this set of assumptions, we need to enroll at least 6,000 women into the evaluation with a 2:1 treatment to control ratio to achieve near 80% or higher power. With these numbers we will have roughly 70% power to detect a statistically significant effect on the birth-spacing measure if the true effect size is -18%. We will have a nearly 100% chance to detect a statistical significant effect on the child injury measure if the true effect size is -13%. We will not have sufficient statistical power to detect the expected effect (-20%) on the pre-term birth measure; the minimum detectable effect size with 80% power for the pre-term birth measure is -30%.

6. Recruitment Methods

6.1. Does the study involve the recruitment of participants?

☐**No: If no, skip to <u>7.1</u>**

☒**Yes: If yes; indicate how, when, where, and by whom participants will be recruited**

Recruitment of participants into the study will be conducted by NFP program staff, including Outreach Specialists, Nurse Supervisors, and Nurse Home Visitors at the implementing agencies. Outreach Specialists are study-specific staff who will conduct outreach with community stakeholders, build awareness of the program, assess client eligibility, conduct the informed consent process, administer the baseline survey, and randomize participants throughout the study enrollment period. Nurse Supervisors & Nurse Home Visitors are program staff that implement NFP and will also complete these study activities as they build their caseload.

NFP has existed in South Carolina for 7 years, and has existing relationships with many service providers in the community. Women can be referred to the NFP program through a variety of channels including, but not limited to: health care providers, WIC clinics, schools, pregnancy testing clinics, other home visiting programs, NFP clients or graduates, and Medicaid. Some

clients are also “self-referred.” NFP aims to partner with these referral sources, as well as with community-based organizations and other agencies serving high-risk pregnant clients, to identify and engage women who may be eligible for the program. Methods for recruiting participants broadly fall into one of four categories:

a) Information about a potential participant is submitted to NFP implementing agencies through the existing referral process, or she reaches out to NFP. The NFP Outreach Specialist will verbally assess whether the eligibility criteria are met and then schedule an in-person meeting for obtaining informed consent and completing study enrollment.
b) A woman meets with an Outreach Specialist or Nurse Home Visitor who is stationed at a referral site (e.g. at a high volume prenatal care or WIC site), and can immediately consent to enroll in the study, if eligible.
c) A potential client submits her information to a centralized website or phone number dedicated to the trial. Outreach staff will then contact the woman by phone to determine eligibility, and schedules an in-person meeting to obtain informed consent and complete study enrollment.
d) South Carolina Department of Health & Human Services (SCDHHS) will provide a list of women who are newly enrolled in Medicaid and pregnant to NFP on a regular basis. NFP will send one letter to each mom newly enrolled in Medicaid letting her know that she may be eligible to participate in a no-cost, confidential study for a chance to receive a home nurse. The letter will not disclose that the study is for first-time moms, in case the letter is intercepted by someone other than the mom. Outreach staff will then contact these potential clients over the phone to describe the study, determine whether these clients are interested in the program, and schedule an in-person meeting. During the in-person meeting, a trained nurse will make the final eligibility determination and conduct study enrollment.

Throughout the enrollment period, three full-time Outreach Specialists will spend 3- 4 days per week at high-volume referral sites engaging clients and conducting study enrollment, as described in b) above. Nurse Home Visitors (NHVs) will also spend time at referral sites enrolling clients, while they build their caseloads.

6.1.1 Recruitment of nurses for nurse survey

Recruitment of nurses will be conducted by the research team, as well as nurse supervisors and their staff.

6.2. Are there any materials that will be used to recruit participants, e.g., emails, posters, and scripts?

☐**No** ☒**Yes: If yes; provide a list of the materials (also include copies with the application)**
A community outreach campaign will be implemented in South Carolina to raise awareness of NFP services. Strategies will include advertisements in social media outlets and local media outlets, such as community newspapers. Outreach posters and flyers will also be placed throughout targeted neighborhoods in strategic venues (e.g., laundromats, bodegas, markets, etc.). Radio and television advertisements (using the same script) will also be utilized to raise awareness of NFP services. These advertisements will allow interested applicants to find out more about the study through a centralized website and phone number dedicated to the trial, which will be managed by NFP Outreach Specialists. An Enrollment Script will be used by NFP

program staff to recruit participants for the program, explain the study and determine eligibility.

For those prospective study participants who miss their scheduled study enrollment visit, the NFP program staff will call the mom to reschedule and send a letter in the mail, as follow-up. (See Survey Reschedule Letter).

6.2.1. Strategies will include sending emails to Nurse Supervisors with suggested email templates to send to their current and former staff to complete the survey. The research team may also send emails to recruit current and former staff.

7. Available Resources

7.1. Describe the feasibility of recruiting the required number of participants within the recruitment period

NFP currently restricts outreach due to limited slots available for new clients. Once the study starts, NFP is confident that the combination of existing referral sources, new outreach strategies, active outreach at referral sites, and outreach to recent Medicaid enrollees will enable the project to recruit the required number of eligible participants per year (n=1,000). NFP currently serves about 800 first-time mothers per year in the same communities that will participate in the study. The study will necessitate that NFP scale up by 25%, which NFP is committed to doing by allocating staff (leadership and project personnel) and marketing resources to increase referrals.

Community organizations that have existing relationships with target families will introduce clients to NFP, acting as a trusted intermediary. Referral networks will also be created through engaging managed care organizations (MCOs), hospitals, Federally Qualified Health Centers, and large physician groups to ensure a robust referral pipeline. Furthermore, agencies outside of the healthcare arena at the local and state level have become referral sources for NFP programs in South Carolina. For example, in Anderson, SC, the Office of the Solicitor (District Attorney) is such a partner, as is the Office of First Steps to School Readiness, whose board of directors consists of top-level decision-makers within state departments and agencies (e.g. Department of Health & Human Services, Department of Social Services, etc.).

7.2. Describe how the Principal Investigator will ensure that a sufficient amount of time will be devoted to conducting and completing the research.

The Principal Investigator is committed to dedicating a sufficient amount of time to conduct and complete the research. She has a demonstrated track-record of conducting and completing large-scale research studies, particularly those that have a long time horizon. The team is actively pursuing additional funding to cover a substantial share of the PI's time.

7.3. Are there research staff members, in addition to the Principal Investigator?

☐ **No: If no, skip to 7.5**

☒ **Yes: If yes; outline training plans to ensure that research staff members are adequately informed about the protocol and study-related duties**

All research and program staff who will have direct interaction with participants and/or access to identifiable information will review Harvard's Human Research Protection Program Plan as part of their initial orientation and will complete (and regularly renew) Harvard's online training curriculum available through the CITI program. In addition, all program staff who are

conducting study enrollment will receive a minimum of eight hours of training by evaluation staff on the protection of human subjects and the foundations of the experimental design. Specifically, program staff who are responsible for enrolling eligible applicants in the study will be trained in obtaining informed consent, administering the baseline survey, explaining random assignment, protecting participants' privacy and confidentiality, and reporting adverse events. We will train the program staff using IRB-approved protocols, scripts, consent forms and survey instruments.

7.4. Describe the minimum qualifications for each research role (e.g., RN, social worker) their experience in conducting research, and their knowledge of local study sites and culture

Outreach Specialists will have at least a high school diploma plus a substantial amount of college coursework. Outreach Specialists will receive training in research, ethics and study protocols. These individuals will have a strong understanding of the local landscape and culture, customs and norms, including how to communicate effectively with formal and informal leaders of the communities being served through the project. The Outreach Specialists already on staff have existing relationships in local communities, and can activate those relationships for the benefit of the project. They also possess the skills to engage new partners and cultivate new relationships. Additionally, many Outreach Specialists have backgrounds in community development and community organizing.

Nurse Supervisors will be registered nurses (RNs) who manage the Nurse Home Visitors. At each agency, they will play a role in outreach and recruitment, managing referrals, and, in some cases, enrollment of subjects. Supervisors will have a deep knowledge of the agency they work for and the community served. Ten such supervisors already have experience managing NFP at their agency. One new supervisor will be hired for this project. This role requires a minimum BSN and preferred master's preparation in nursing or a related field.

Nurse Home Visitors will also be RNs with a Bachelor of Science Degree in Nursing who have a very strong knowledge of the study sites. The majority of the nurses already on staff have prior experience implementing the NFP program at the study sites, and several of them have been implementing the program since 2008 when NFP began implementation in South Carolina. Seventeen new nurses will also be hired for this project. These individuals will receive training in research, ethics and study protocols and may have past experience conducting research. A majority of the nurses are residents of the communities being served, and have a background in serving those communities prior to coming to NFP.

Nurse Supervisors and Nurse Home Visitors are trained to screen for various abuses (including substance abuse and domestic violence) and to make referrals to local support services. They are also mandated reporters in the state of South Carolina. As mandated reporters, they must report to the appropriate authorities when they have reason to believe, in their professional capacity, that a child has been abused or neglected.

7.5. Briefly describe how the research facilities and equipment at the research site(s) support the protocol's aims, e.g., private rooms available for interviews, etc.

NFP program staff who are conducting study enrollment at the NFP implementing agencies will be provided private rooms to conduct interviews, and secure tablets with encrypted survey forms to collect baseline data and randomize participants.

For clients who are approached at referral sites, NFP staff may initially engage clients in a public space (e.g. the waiting room at a health clinic or a table at a WIC clinic). Study staff will secure private space for obtaining informed consent and conducting interviews. In some instances, the study consent process and baseline interview may be completed in a follow-up meeting at an agreed upon location, such as the woman's home or another neutral, private location.

NFP staff will be provided with a toll-free number that they can call to reach the core research team to ask questions or report adverse events. This toll-free number will be monitored during standard business hours. NFP staff will also be provided with an email address (nfpstudy@povertyactionlab.org) that is dedicated to this study and monitored on a regular basis.

7.6. Are there provisions for medical and/or psychological support resources (e.g., in the event of incidental findings, research-related stress)?

☐**No** ☒**Yes: If yes; describe the provisions and their availability**

There is limited risk of research-related stress. However, NFP program staff are registered nurses trained to provide resources/referrals to help pregnant women deal with stress.

7.6.1 Nurse survey: Are there provisions for medical and/or psychological support resources (e.g., in the event of incidental findings, research-related stress)? Yes, for nurses who are currently employed by NFP.

There is limited risk of research-related stress. However, if current NFP nurses experience stress as a result of the survey, their organizations already have systems in place for nurses to bring any thoughts, questions, or concerns that may come about by the research. These resources and support systems include, but are not limited to, case conferences, reflective supervision, team meeting education modules, and employee assistant programs which are available at many sites. We provide information about resources and support systems in the consent form and encourage nurses to reach out to their nurse supervisors and colleagues if they need information on how to access these services. We also inform nurses that they can contact the research team directly if they do not know who to contact, or do not feel comfortable contacting someone at their site directly.

These resources and supports are not necessarily available to former NFP nurses and are not included in the consent form for former NFP nurses.

2. Vulnerable Populations

2.1. Are there any potentially vulnerable populations (e.g., children, pregnant women, human fetuses, neonates, prisoners, elderly, economically disadvantaged, employees or students of the investigator or sponsor, undocumented, terminally ill, cognitively impaired or mentally ill, etc.)?

☐**No: If no, skip to 3.1**

☒**Yes: If yes; identify all vulnerable populations**

Pregnant women, neonates, children

2.2. Describe safeguards to protect their rights and welfare

This research study involves no more than minimal risk to pregnant women and their children. The research is designed to evaluate a service program that is designed specifically to provide additional resources to these populations, which is why enrollment focuses on them. Information on children will be obtained from administrative data and only with their mother's written informed consent.

Nurse Supervisors and Nurse Home Visitors are trained to screen for various abuses (including substance abuse and domestic violence) and to make referrals to local support services. They are also mandated reporters in the state of South Carolina. As mandated reporters, they must report to the appropriate authorities when they have reason to believe, in their professional capacity, that a child has been abused or neglected.

If the mandated reporter has reason to believe that a child is being abused or neglected by a parent, guardian or person responsible for a child's welfare, reports must be made to the Department of Social Services (DSS) office or law enforcement agency in the county in which the child resides.

If the mandated reporter has reason to believe that a child is being abused or neglected by someone other than a parent, guardian or person responsible for a child's welfare, reports must be made to a law enforcement agency in the county in which the child resides.

The nurses will comply with state laws that require them to report if they have reason to believe a child is being abused or neglected.

3. Consent Process

3.1. Will consent to participate be obtained?

☐ **No: If no, skip to <u>3.5</u>**

☒ **Yes: If yes; describe the setting, role of individuals involved, timeframe(s), and steps to minimize coercion/undue influence during the consent process (at the time of initial consent and throughout the study)**

NFP program staff will be trained by the study team about how to implement the informed consent process. To minimize coercion/undue influence, program staff will allow the participant as much time as she needs to read through the consent form and will also review the consent form with her verbally (estimated time: 10-15 minutes). The program staff will emphasize that participation in the study is voluntary, and that participants may refuse to answer questions or may choose to stop participating in the study at any time. Program staff will be trained to check for participant's comprehension of what it means to be in the study (particularly their understanding of the random assignment process) and will encourage study participants to ask questions now and in the future, if they have them.

Based on the local population in South Carolina, the majority of participants will speak English as their primary language. The consent materials will also be translated into Spanish (see Translation Attestation Form attached). Occasionally, participants may speak another language besides English or Spanish, but based on existing enrollment patterns we expect this to be less than 0.5% of cases. In those rare cases, NFP program staff will utilize an interpreter service through the health care center where they work to facilitate the informed consent process.

Due to COVID-19, informed consent will be obtained virtually over the phone. Nurses are trained in motivational interviewing and will continue to check for participant's comprehension of what it means to be in the study, the understanding of the randomization process, and encourage questions. Written informed consent will be waived during this period and up until in person study visits are possible.

3.2. Are there any special populations?

☐No ☒Yes: If yes; describe the process to obtain consent, permission or assent

NFP currently targets its services to vulnerable young mothers, including pregnant minors (under 18 years old), who have the highest need of support during their pregnancy and first years as a parent. The use of Medicaid funds to cover NFP for the expansion population in South Carolina is predicated on an evaluation of its effects using this randomized controlled design. The study is the only pathway through which anyone who meets the inclusion criteria in South Carolina, including pregnant minors, can access NFP.

South Carolina law does not require parental consent for minors to seek healthcare services, including reproductive healthcare services. In accordance with this law, and to protect the well-being of the young mothers who may be endangered if they are required but not able to obtain parental permission, it is necessary to allow an avenue for pregnant minors to provide their own consent to participate in the study. If pregnant minors were not allowed to provide their own consent, and were thus excluded from study enrollment if they could not practicably obtain parental consent, this would deny them access to Medicaid-funded NFP services – which would be inconsistent both with the goals of providing additional resources to this vulnerable population and with the laws and regulations in South Carolina. (Currently, the median age of pregnant women who receive NFP services in South Carolina is 19, meaning that a substantial share of those served are minors.[iii])

NFP staff are trained to communicate and provide care to this special population of pregnant minors, and will be implementing the informed consent process with them. During the consent process, NFP staff will take special care to make sure that each pregnant minor understands that her participation in the research study is voluntary and that she can quit at any time without penalty.

NFP staff will invite each pregnant minor to provide an electronic signature if she wishes to participate in the study. The pregnant minor's electronic signature will provide a permanent record of 1) the information conveyed to the mother about the study, 2) the fact that the consent process occurred, and 3) the mother's willingness to participate. By default, NFP staff will not provide pregnant minors (under 18) with a copy of the unsigned consent form, in case her family does not yet know she is pregnant. Instead, NFP staff will provide pregnant minors with the Principal Investigator's email address (mmcconne@hsph.harvard.edu) and/or the IRB's toll-free number (1-866-606-0573) in case she later has questions, concerns or complaints about this research study.

However, if a participant under 18 years old actively asks for a copy of the unsigned consent form, or if a guardian who is present at the time of study enrollment actively asks to keep a

[iii] http://www.nursefamilypartnership.org/assets/PDF/Communities/State-profiles/SC_State_Profile.aspx

copy of the unsigned consent form, a copy of the consent form will be provided. In these situations, it is implied that the minor is willing to share the information about her pregnancy with her family. During the consent process, the nurse will remind the minor that she is free to discuss her decision to participate in the research with her family, friends, or doctors, but that she does not need her guardian's permission to participate in the study.

NFP nurses will comply with state laws that require them to report if they have reason to believe a child is being abused or neglected. During the informed consent process, NFP staff will make the participant aware that they are mandated reporters who are required to report to the appropriate authorities if they have reason to believe a child is being abused or neglected. They will explain to the participant that it is their legal and ethical responsibility to report situations of child abuse, child neglect, or any life-threatening situation to appropriate authorities.

3.3. Will consent of the participants be documented in writing?
☒Yes ☐No: If no; describe the rationale for requesting a waiver or alteration of documentation of consent (and/or parental permission)
We will obtain an electronic signature from subjects who consent to participate. Due to COVID-19 we will waive written consent and only obtain verbal consent until in person study visits are possible.

3.4. Will participants be provided with a copy of their signed consent form or information sheet (when a consent form is not signed)?
☐Yes ☒No: If no; explain any extenuating circumstances that make it impossible or inappropriate to meet this requirement, i.e., doing so may place participants at increased risk, if inadvertently disclosed
Participants 18 years or older will be provided a copy of the unsigned consent form/information sheet to take home that is identical to the one she signed, but does not include her signature. This will be done to protect the participant's privacy in the case where she inadvertently loses or misplaces the information sheet (because the version she has will not contain her name).

Participants under 18 years old will not be provided a copy of the unsigned consent form by default, to protect their privacy, safety and well-being. Providing information sheets to pregnant minors may put them at increased risk, particularly in cases where her family does not yet know she is pregnant. Instead, NFP staff will provide pregnant minors with the Principal Investigator's email address (mmcconne@hsph.harvard.edu) and/or the IRB's toll-free number (1-866-606-0573) in case she later has questions, concerns or complaints about this research study. However, if a participant under 18 years old actively asks for a copy of the unsigned consent form, or if a guardian who is present at the time of study enrollment actively asks to keep a copy of the unsigned consent form, a copy of the consent form will be provided. In these situations, it is implied that the minor is willing to share the information about her pregnancy with her family. During the consent process, the nurse will remind the minor that she is free to discuss her decision to participate in the research with her family, friends, or doctors, but that she does not need her guardian's permission to participate in the study.

Due to COVID-19, a copy of the unsigned consent form//information sheet will be mailed to clients.

3.5. Is a waiver or alteration of consent (and/or parental permission) being requested?
☐No ☒Yes: If yes; describe the rationale for the request. If the alteration is because of deception or incomplete disclosure, explain whether and how participants will be debriefed (include any debriefing materials with the application)

NFP currently targets its services to vulnerable young mothers, including pregnant minors (under 18 years old), who have the highest need of support during their pregnancy and first years as a parent. The use of Medicaid funds to cover NFP for the expansion population in South Carolina is predicated on an evaluation of its effects using this randomized controlled design. The study is the only pathway through which anyone who meets the inclusion criteria in South Carolina, including pregnant minors, can access NFP.

South Carolina law does not require parental consent for minors to seek healthcare services, including reproductive healthcare services. In accordance with this law, and to protect the well-being of the young mothers who may be endangered if they are required but not able to obtain parental permission, it is necessary to allow an avenue for pregnant minors to provide their own consent to participate in the study. If pregnant minors were not allowed to provide their own consent, and were thus excluded from study enrollment if they could not practicably obtain parental consent, this would deny them access to Medicaid-funded NFP services – which would be inconsistent both with the goals of providing additional resources to this vulnerable population and with the laws and regulations in South Carolina. (Currently, the median age of pregnant women who receive NFP services in South Carolina is 19, meaning that a substantial share of those served are minors.[iv])

NFP staff are trained to communicate and provide care to this special population of pregnant minors, and will be implementing the informed consent process with them. During the consent process, NFP staff will take special care to make sure that each pregnant minor understands that her participation in the research study is voluntary and that she can quit at any time without penalty.

NFP staff will invite each pregnant minor to provide an electronic signature if she wishes to participate in the study. The pregnant minor's electronic signature will provide a permanent record of 1) the information conveyed to the mother about the study, 2) the fact that the consent process occurred, and 3) the mother's willingness to participate. By default, NFP staff will not provide pregnant minors (under 18) with a copy of the unsigned consent form, in case her family does not yet know she is pregnant. Instead, NFP staff will provide pregnant minors with the Principal Investigator's email address (mmcconne@hsph.harvard.edu) and/or the IRB's toll-free number (1-866-606-0573) in case she later has questions, concerns or complaints about this research study.

However, if a participant under 18 years old actively asks for a copy of the unsigned consent form, or if a guardian who is present at the time of study enrollment actively asks to keep a copy of the unsigned consent form, a copy of the consent form will be provided. In these

[iv] http://www.nursefamilypartnership.org/assets/PDF/Communities/State-profiles/SC_State_Profile.aspx

situations, it is implied that the minor is willing to share the information about her pregnancy with her family. During the consent process, the nurse will remind the minor that she is free to discuss her decision to participate in the research with her family, friends, or doctors, but that she does not need her guardian's permission to participate in the study.

4. Risks

4.1. Are there any <u>reasonably foreseeable</u> risks, discomforts, and inconveniences to participants and/or groups/communities?

☐No ☒Yes: If yes; indicate probability, magnitude, and duration of each (note that risks may be physical, psychological, social, legal, and/or economic)

Taking part in this research involves minimal risk. There is always a small risk that the mother or children's Protected Health Information (PHI) will be seen by someone who is not on the study team. However, the study team will follow strict rules to protect the mother and children's privacy and confidentiality. The study team will adhere to Harvard's Level 4 data security requirements to protect participants' PHI.

There is one exception to privacy/confidentiality that we will make participants aware of during the informed consent process: NFP staff are mandated reporters and will comply with state laws that require them to report to authorities if they have reason to believe a child is being abused or neglected. It is their legal responsibility to report situations of child abuse, child neglect, or any life-threatening situation to appropriate authorities.

4.2. Identify whether any of the information collected, if it were to be disclosed outside of the research, could <u>reasonably</u> place the participant at risk of criminal or civil liability or be damaging to the participant's financial standing, employability or reputation.

We are not collecting any sensitive information (e.g. illegal drug use) in the baseline survey such that if it were to be disclosed outside of the research team, the participant could reasonably be placed at risk. For the analysis of the outcome measures, we are using administrative databases already in possession of the state, so there is also minimal risk that if this information were disclosed, it could reasonably be damaging to the participant's financial standing, employability, or reputation. If PHI were disclosed, the participant could experience emotional or financial distress. However, the study team will follow strict data security requirements to protect this information from being released, as discussed below.

There is one exception to privacy/confidentiality that we will make participants aware of during the informed consent process: NFP staff are mandated reporters and will comply with state laws that require them to report to authorities if they have reason to believe a child is being abused or neglected. It is their legal responsibility to report situations of child abuse, child neglect, or any life-threatening situation to appropriate authorities.

4.3. Outline provisions in place to minimize risk

All study team members will access to confidential information will both acknowledge a confidentiality agreement and be appropriately trained.

Identifying information will always be kept separate from the datasets that will be used for analysis. The datasets used for analysis will be stripped of personal identifiers such as name,

address, record number, and replaced with a unique, scrambled study identifier. Indirect identification may be possible, but no attempts will be made to identify individual study participants, and the study team will adhere to strict data security protocols to protect participants' confidentiality.

Confidential information will never be sent via email except in encrypted files. Designated study staff will use encrypted files and/or a secure File Transfer Portal (FTP) to transfer personal identifiers to the data agencies for selecting administrative records. After the data agency staff-person selects the administrative records for the study participants, he will strip off any personal identifiers, keep the study ID, and send back a limited dataset to the study team through the secure FTP.
For cases in which the study team needs to go "onsite" to conduct a match with administrative data, study staff will follow the procedures below which have been approved by the HSPH Information Security Specialist, Ingrid Skoog:

1. On the Level 4 Server, study staff will create a "finder file" that includes the study participants' unique study ID and personal identifiers. This file will be saved as an encrypted .7z file.
2. This "finder file" will be transferred securely via VPN to an encrypted, external hard-drive.
3. Study staff will bring the encrypted hard-drive to the partner's site, transfer the partners' administrative database (including identifiers) to the external hard-drive, and then connect the hard-drive to a laptop that (1) doesn't connect to the internet (2) is full disk encrypted and (3) gets reimaged/wiped after each use.
4. Study staff will conduct the match on the laptop, using the personal identifiers.
5. After the study staff has selected the administrative records for study participants, she will create three de-identified files to bring back to Cambridge on the encrypted, external hard-drive:
    a. A cross-walk that includes only the unique study ID and the ID from the administrative data
    b. A file that includes only the administrative outcomes data and the ID from the administrative data. (This file will include no personal identifiers.)
    c. The code used to generate these two files. (The code will include no personal identifiers).
6. All other files, besides the three de-identified files mentioned above, will be deleted from both the external hard-drive and the laptop while on-site.
7. Upon return to Cambridge, the three de-identified files will be securely transferred from the encrypted hard-drive to the Level 4 server via VPN, and the laptop will get re-imaged/wiped by IT, so that all of the files on the laptop are permanently deleted.

All data will be stored on an institutional stationary server that only designated study staff will have access to. A written list of those designated study staff will be disclosed to the IRB. Each study staff-person who has access to the Level 4 server will be given an individually assigned (non-shared) user account. Their access to the Level 4 data or servers will be removed if they no longer have a reason under the access policy to access the information (e.g., they change jobs or leave the university).

In addition, a qualified Server Administer will maintain the Level 4 server at Harvard where the data will be stored. The server administrator will:

- require complex passwords from individual users (no shared access);
- permit access to only those users for whom access has been established;
- inhibit password guessing attacks;
- not be allowed to retrieve user passwords;
- use a secure mechanism to update and/or change passwords;
- keep the operating system and application patches current;
- use the applicable malware detection software will be run with up-to-date signature files;
- force re-authentication to user accounts after an idle period;
- protect from improper network-based access, physical theft and loss;
- log user and administrator access to servers; and
- review logs periodically for anomalous behavior.

Limited data sets extracted from the Level 4 server will be stored on MIT Dropbox Enterprise for collaboration and analysis. These data sets will not contain any individual identifiers such as name, contact information, address, SSN, or any account number. However, they may contain individually relevant dates such as date of admissions and date of discharge. Such external collaboration and analysis may include but is not exclusive to extracting deidentified data for analysis on external web applications which is not possible from the Level 4 server. The sole purpose being to generate new variables such as growth Z-Scores standardized to National benchmarks which will enhance our analysis. Only the designated study staff will have access to the limited data sets on Dropbox. All local copies of data will be deleted upon project completion.

All electronic records containing confidential information will be properly disposed of by overwriting the information. Any publications of analysis will include only aggregated data not linked to any individual.

5. Benefits

5.1. Describe potential benefits of study participation (indicate if there is no direct benefit)

For participating in the study, participants gain the opportunity to have access to additional information and support through NFP, which may improve their and their children's outcomes.

5.2. Describe potential benefits of the research to the local community and/or society

The study will provide information to help improve services for pregnant women and parents with young children. Programs like NFP have the potential to substantially improve long-run outcomes including maternal and child health, and are of substantial interest to local and national policy-makers and stakeholders.

6. Reportable Events

6.1. Outline plans for communicating reportable events (e.g., adverse events, unanticipated problems involving risks to participants or others, breach of confidentiality)

All NFP program staff who are conducting study enrollment will be trained to report protocol violations or unanticipated problems involving risk to participants or others to the Principal

Investigator within 24 hours of the specific event. The Principal Investigator will communicate adverse events, including any potential breaches of confidentiality, within 5 business days to the IRB, who will make the final determination as to whether a particular event constitutes an unanticipated problem involving risks to participants or others.

NFP staff are mandated reporters and will comply with state laws that require them to report to authorities if they have reason to believe a child is being abused or neglected. It is their legal responsibility to report situations of child abuse, child neglect, or any life-threatening situation to appropriate authorities.

If the mandated reporter has reason to believe that a child is being abused or neglected by a parent, guardian or person responsible for a child's welfare, reports must be made to the Department of Social Services (DSS) office or law enforcement agency in the county in which the child resides.

7. **Research Related Injuries (this section must be completed for any greater than minimal risk research)**

13.1 Are there provisions for medical care and compensation for research-related injuries?

☒**No** ☐**Yes: If yes; outline these provisions (Please note that although Harvard's policy is not to provide compensation for physical injuries that result from study participation, medical treatment should be available including first aid, emergency treatment and follow-up care as needed. If the research plan deviates from this policy, provide appropriate justification.)**

8. **Participant Privacy**

8.1. Describe provisions to protect participants' privacy (their desire to control access of others to themselves, e.g., the use of a private interview room) and to minimize any sense of intrusiveness that may be caused by study questions or procedures

All study staff will be trained to protect participants' privacy. Study enrollment and surveys will be completed in private settings and will not be shared with anyone beyond the study staff. All information about individual participants will be marked with a unique code number, not with the participant's name or any other identifying information. No reports will describe participants in a way that would allow any individual participant to be identified.

There is one exception to privacy/confidentiality that we will make participants aware of during the informed consent process: NFP staff are mandated reporters and will comply with state laws that require them to report to authorities if they have reason to believe a child is being abused or neglected. It is their legal responsibility to report situations of child abuse, child neglect, or any life-threatening situation to appropriate authorities.

9. **Data Confidentiality**

9.1. Will the information that is obtained be recorded in such a manner that participants can be identified, directly or through identifiers linked to the participants?

☐**No: If no, skip to <u>10.1</u>**

☒**Yes: If yes; either state that participants will be told that their data will be public or describe provisions to maintain the confidentiality of identifiable data, e.g., use of password protections (please refer to the Harvard Research Data Security Policy Protection Memo, at http://www.security.harvard.edu/harvard-research-data-security-policy-protection-memo,**

for additional information about required data security measures) [NOTE: Harvard Research Data Security Policy does not always apply if data are not being stored at Harvard facilities. Please consult the Data Security Policy for additional information.]
Identifying information will always be kept separate from the datasets that will be used for analysis. As discussed below, the datasets used for analysis will be stripped of personal identifiers such as name, address, record number, and replaced with a unique, scrambled study identifier. Indirect identification may be possible, but no attempts will be made to identify individual study participants, and the study team will adhere to strict data security protocols, described below, to protect participants' confidentiality.

Confidential information will never be sent via email except in encrypted files. Designated study staff will use encrypted files and/or a secure File Transfer Portal (FTP) to transfer personal identifiers to the data agencies for selecting administrative records. After the data agency staff-person selects the administrative records for the study participants, he will strip off any personal identifiers, keep the study ID, and send back a limited dataset to the study team through the secure FTP.

For cases in which the study team needs to go "onsite" to conduct a match with administrative data, study staff will follow the procedures below which have been approved by the HSPH Information Security Specialist, Ingrid Skoog:

1. On the Level 4 Server, study staff will create a "finder file" that includes the study participants' unique study ID and personal identifiers. This file will be saved as an encrypted .7z file.
2. This "finder file" will be transferred securely via VPN to an encrypted, external hard-drive.
3. Study staff will bring the encrypted hard-drive to the partner's site, transfer the partners' administrative database (including identifiers) to the external hard-drive, and then connect the hard-drive to a laptop that (1) doesn't connect to the internet (2) is full disk encrypted and (3) gets reimaged/wiped after each use.
4. Study staff will conduct the match on the laptop, using the personal identifiers.
5. After the study staff has selected the administrative records for study participants, she will create three de-identified files to bring back to Cambridge on the encrypted, external hard-drive:
    a. A cross-walk that includes only the unique study ID and the ID from the administrative data
    b. A file that includes only the administrative outcomes data and the ID from the administrative data. (This file will include no personal identifiers.)
    c. The code used to generate these two files. (The code will include no personal identifiers).
6. All other files, besides the three de-identified files mentioned above, will be deleted from both the external hard-drive and the laptop while on-site.
7. Upon return to Cambridge, the three de-identified files will be securely transferred from the encrypted hard-drive to the Level 4 server via VPN, and the laptop will get re-imaged/wiped by IT, so that all of the files on the laptop are permanently deleted.

All data will be stored on an institutional stationary server that only designated study staff will have access to. A written list of those designated study staff will be disclosed to the IRB. Each study staff-person who has access to the Level 4 server will be given an individually assigned (non-shared) user account. Their access to the Level 4 data or servers will be removed if they no longer have a reason under the access policy to access the information (e.g., they change jobs or leave the university). A locking screen-saver will be in place on the server to block staff's access to idle sessions.

All electronic records containing confidential information will be properly disposed of by overwriting the information. Any publications of analysis will include only aggregated data not linked to any individual.

There is one exception to privacy/confidentiality that we will make participants aware of during the informed consent process: NFP staff are mandated reporters and will comply with state laws that require them to report to authorities if they have reason to believe a child is being abused or neglected. It is their legal responsibility to report situations of child abuse, child neglect, or any life-threatening situation to appropriate authorities.

9.2. Describe i) whether data will be transmitted, and if so how; ii) how long it will be stored; and iii) plans for the data at the end of the storage period (how will it be destroyed, or will it be returned to data provider)

At study enrollment, program staff (trained in human subjects protections) will collect personal identifiers from each participant including first name, last name, date of birth, social security number, address, Medicare Health Insurance Claim Number (HICN) (if applicable), and Medicaid Statistical Information System ID (if applicable). Each study participant will be assigned a unique study ID that does not contain any identifying information (e.g. will not contain the participant's initials or last four digits of Social Security Number). This information will be collected in an encrypted survey form, and will be transferred securely to a Level 4 Server at Harvard through a File Transfer Portal (FTP).

The personal identifiers and unique study IDs will be stored in a 'crosswalk' file, separate from all other data used for analysis. The crosswalk file will meet Harvard's Level 4 data security requirements. For example, only designated research staff will have access to the crosswalk file on a secure server at Harvard. A qualified Server Administer will maintain the server at Harvard where this cross-walk file will be stored. The server administrator will:

- require complex passwords from individual users (no shared access);
- permit access to only those users for whom access has been established;
- inhibit password guessing attacks;
- not be allowed to retrieve user passwords;
- use a secure mechanism to update and/or change passwords;
- keep the operating system and application patches current;
- use the applicable malware detection software will be run with up-to-date signature files;
- force re-authentication to user accounts after an idle period;
- protect from improper network-based access, physical theft and loss;
- log user and administrator access to servers; and
- review logs periodically for anomalous behavior

A backup copy of data collected at study enrollment will be downloaded to an encrypted external hard drive that only designated study staff will have access to. A written list of those designated staff will be disclosed to the IRB. Due to Covid-19 Professor McConnell will keep the external hard drive at her home. The hard drive will remain in her locked office at the conclusion of the pandemic.

At agreed-upon time intervals, designated research staff will use a secure File Transfer Portal (FTP) to transfer the "crosswalk" file (with study IDs and personal identifiers) to the state data agency. The data agency will use the personal identifiers in the crosswalk to match administrative records to our list of study participants. The data agency will then send back the matched data, but strip off all personal identifiers (leaving study IDs). This limited data file will be stored on the Level 4 server, as well, since it may considered individually identifiable per HIPAA regulations. The limited data file will be used for analysis and will never be matched to the crosswalk file that contains the personal identifiers. A copy of the limited data set may be kept on MIT Dropbox Enterprise for analysis and collaboration. MIT Dropbox, unless otherwise specified in a DUA.

For cases in which the study team needs to go "onsite" to conduct a match with administrative data, study staff will follow the procedures below which have been approved by the HSPH Information Security Specialist, Ingrid Skoog:

1. On the Level 4 Server, study staff will create a "finder file" that includes the study participants' unique study ID and personal identifiers. This file will be saved as an encrypted .7z file.
2. This "finder file" will be transferred securely via VPN to an encrypted, external hard-drive.
3. Study staff will bring the encrypted hard-drive to the partner's site, transfer the partners' administrative database (including identifiers) to the external hard-drive, and then connect the hard-drive to a laptop that (1) doesn't connect to the internet (2) is full disk encrypted and (3) gets reimaged/wiped after each use.
4. Study staff will conduct the match on the laptop, using the personal identifiers.
5. After the study staff has selected the administrative records for study participants, she will create three de-identified files to bring back to Cambridge on the encrypted, external hard-drive:
    a. A cross-walk that includes only the unique study ID and the ID from the administrative data
    b. A file that includes only the administrative outcomes data and the ID from the administrative data. (This file will include no personal identifiers.)
    c. The code used to generate these two files. (The code will include no personal identifiers).
6. All other files, besides the three de-identified files mentioned above, will be deleted from both the external hard-drive and the laptop while on-site.
7. Upon return to Cambridge, the three de-identified files will be securely transferred from the encrypted hard-drive to the Level 4 server via VPN, and the laptop will get re-imaged/wiped by IT, so that all of the files on the laptop are permanently deleted.

9.3. Indicate how research team members and/or other collaborators are permitted access to information about study participants

Only designated research team members will be permitted access to information about study participants. Each team member who is designated access will be required to have individual credentials and a protected password that will never be shared; the password must be different from any other password used for another Harvard or personal accounts, must be of sufficient length and complexity to reasonably protect it from being guessed by humans or computers, and must be changed immediately if there is suspicion of compromise. Confidential information will only be accessed for authorized purposes and never shared with someone who is not authorized to receive it.

9.4. If future open access, i.e., free availability and unrestricted use, of data is planned or likely, indicate how data will be released.

The creation of a public-use de-identified data set is planned for the end of the study period (subject to data use agreements). This data set will be purged of any small cell values that might permit indirect identification of participants or any proprietary information.

10. Costs and Payments

10.1. Identify any costs that participants may incur during the study, including transportation costs, childcare, or other out-of-pocket expenses.

Participants will not incur any costs for participating in the study.

10.2. Is there any payment or reimbursement that participants may receive during the study?

☐No ☒Yes: If yes; specify the amount, method and timing of disbursement. (Please refer to Harvard University Financial Policy on Human Subject Payments at http://vpr.harvard.edu/sites/vpr.harvard.edu/files/news/Human%20Subject%20Payments%20Policy%20Final_0.pdf)

Participants who enroll in the study will be provided with a $25 gift card to compensate them for their time in completing the baseline survey, regardless of whether they are randomized to the treatment group or the control group. Due to COVID-19, gift cards will be mailed to clients to compensate them for their time for both treatment and control group.

11. Multi-site Study Management

11.1. Is this a multi-site study?

☐No ☒Yes: If yes; describe plans for communication among sites regarding adverse events, interim results, protocol modifications, monitoring of data, etc.

The NFP program has a long-standing, centralized National Service Office that oversees communication with NFP implementing agencies. The study team has regular communication with the National Service Office, and will keep them abreast throughout the study period of any adverse events, interim results, or protocol modifications that should be shared with NFP implementing agencies. In consultation with the National Service Office, the study team will determine the best way to communicate these updates to the NFP implementing agencies, depending on the content and urgency of the message (e.g. via email, phone, etc.).

12. Investigational Drug/Biologic/Device

12.1. Does this study involve an Investigational Drug/Biologic/Device?

☒No: If no; skip to <u>13.1</u>

☐**Yes: If yes; identify and describe the drug/biologic/device (e.g., marketing status: Is there an IND/IDE, classification of a device as significant vs. non-significant risk)**

12.2. Describe its administration or use

12.3. Compare the research drug/biologic/device to the local standard of care

12.4. Describe plans for receiving, storage, dispensing and return (to ensure that they will be used only for participants and only by authorized investigators)

12.5. If proven beneficial, describe anticipated availability and cost to participants post-study; plans (if applicable) to make available

13. HIPAA Privacy Protections

13.1. Are HIPAA privacy protections required? Please note that only Harvard University Health Services and Harvard School of Dental Medicine are covered entities at Harvard. Harvard is otherwise not a HIPAA covered entity. If, however, data is derived from a Covered Entity (e.g. a hospital or community health center), mark 'yes' and address the items below.

☐**No: If no; skip to <u>14.1</u>**

☒**Yes: If yes; include at least one of the following:**
Describe plans for obtaining authorization to access protected health information
Provide the rationale for a waiver of authorization or limited waiver of authorization request

We used Harvard's approved template for 'Consent at HIPAA covered entities.' This template provides clear language about"

- The privacy rules of the HIPAA law, including a definition of PHI, and participants' rights under the law
- Who will be able to see the PHI for research purposes
- What types of PHI will be collected
- Where the PHI will be collected from
- How the PHI will be protected to maintain each participant's privacy and confidentiality
- How the PHI will used

The consent form and the NFP staff who will be conducting the informed consent process will make it clear to the participant that by signing the consent form, she is authorizing the study team to use her and her children's PHI. The participant will also be informed that she may cancel this authorization at any time by contacting the study team, in writing, at the address on the consent form. If she decides not to authorize the investigators to use her PHI or she cancels this authorization, she and her children will no longer be able to participate in this research study, and the use or sharing of future PHI will be stopped. However, the PHI that has already been collected may still be used.

14. Data and Specimen Banking

14.1. Does the study include Data and Specimen Banking?

☒**No: If no; skip to <u>15.1</u>**

☐**Yes: If yes; identify what will be collected and stored, and what information will be associated with the specimens**

14.2. Describe where and how long the data/specimens will be stored and whether participants' permission will be obtained to use the data/specimens in other future research projects

14.3. Identify who may access data/specimens and how

14.4. Will specimens and/or data be sent <u>to</u> research collaborators outside of Harvard?
☐**No** ☐**Yes: If yes; describe the plan**

20.5 Will specimens and/or data be received <u>from</u> collaborators outside of Harvard?
☐**No** ☐**Yes: If yes; describe the plan**

15. Sharing Study Results

15.1. Is there a plan to share study results with individual participants?
☒**No** ☐**Yes: If yes; describe the plan**

15.2. Is there a plan to disseminate aggregate results to the community where the research is conducted?
☐**No** ☒**Yes: If yes; describe the plan**

Results from the study will be disseminated to academics, the public, and the policy community. The study can provide state and federal policy-makers much needed information about the effect of expanding or implementing home visiting programs, taxpayers and voters information about the use of public resources to subsidize such programs, and participants information about the efficacy of such interventions.

16. Regulatory Compliance

16.1. Describe plan for monitoring regulatory compliance, in order to ensure proper record keeping and retention of required regulatory documents

The software (SurveyCTO) will time and date stamp the recording of informed consent, the baseline survey, and the randomization. The study team will monitor the data from this in real time to ensure that protocol is followed (including that consent, survey, and randomization were done in the proper order) and that the clients, the field offices, and the recruiters did not tamper with the randomization. Additionally, we will work with NFP administrators to ensure the overall fidelity of the implementation to the evaluation plan through random back checks, site visits, and monitoring of other data.

The PI will make sure that all investigators and study staff have up-to-date Human Research Training certificates on file. The PI will also ensure that her CV, as well as the CVs of the other co-investigators, have been updated within the past 2 years, and that they are signed and dated and centrally stored.

The study team will also keep any grant progress reports, correspondence to and from funding agencies, signed agreements/contracts, external IRB approvals, and a roster of members with documentation of HSPH's Federalwide Assurance (FWA) Number on file throughout the course of the study.

Pre-Analysis Plan: Impact of Nurse-Family Partnership on Maternal and Early Child Outcomes

Note: This is version 2 of the pre-analysis plan reflecting plans for study design as of January 20, 2021. The first unblinding is planned for January 21, 2021 when interim analyses will be prepared for Pay-for-Success payments. The previous version of this pre-analysis plan (version 1) is available on our study website.

A full study protocol describing study motivation, design, and plans for analysis has been published in McConnell et al. (2020).[1] We do not repeat those materials here. In this pre-analysis plan, we provide more detail about our planned analytical choices including analytic details for defining primary and secondary outcomes for the three domains of analysis defined in the study protocol in Addendums 1-3. This analysis plan will be updated as analytical plans for additional longitudinal outcomes are developed.

1. Methods

While other methods are outlined in the study protocol, in this pre-analysis plan we provide further details on methods related to sample construction and analytical plans.

A. Sample construction

A.1. Tracking study moms and babies

All study outcomes will be measured using administrative data sources, which require matching to state administrative records. Because we plan to analyze outcomes for both the mother and child, we define several relevant samples for analysis of outcomes for the mother-child dyad. For most analyses, we will want to consider the sample of mother-baby dyads where a live birth occurred from the pregnancy in gestation at the time of enrollment. We call this the index birth. Mother-baby dyads who experience an index birth will be the primary sample for longitudinal analyses examining the impact of the program. As the mother is the unit of randomization and we expect multiple births to be rare, we plan to aggregate outcomes pertaining to children resulting from the index birth to the mother level. To identify the index birth, we first match the mother to a birth certificate in vital records. If the date of birth on the birth certificate is within 120 days before or after the estimated delivery date as reported on the baseline survey, we will consider the birth to be related to the pregnancy that was in gestation at the time of study enrollment. Births that occur outside of this window will not be included in the sample of index births.

We may also analyze outcomes for the broader sample of mothers with at least one matched administrative record related to the pregnancy reported at the time of enrollment. We will call this the index pregnancy. We define the index pregnancy as follows: when there is a matched

index birth or fetal death in vital records[v], we define the pregnancy period as the period covering the weeks of gestation provided in the obstetric estimate on the vital record until the date of birth or fetal death. In the absence of an index birth or fetal death, we define the pregnancy period as the period covering 42 weeks prior to the expected delivery date provided on the baseline survey. We will consider a mother to be matched to the index pregnancy if she has an any matched administrative records pertaining to the index pregnancy (including antenatal care, probable pregnancy loss or birth related records from Medicaid claims or hospital discharge or evidence of a birth or fetal death from vital records). Table 1 summarizes the definitions and data sources used to identify index pregnancies and live index births.

When tracking children in the study, we will define two key periods. We anticipate that the children of most mothers who enroll in the trial will be eligible for Medicaid for their entire first year of life, as the income requirements for infants are similar to those of pregnant women. However, as summarized in Table 2, in some years of the study, income requirements became more restrictive after the first year of the child's life. Furthermore, families whose children remain income-eligible into their child's second year of life must submit an annual review form to retain coverage. Therefore, we plan to measure outcomes for children resulting from index births in the study to two critical time points: through their first 12 months of life and through their first 24 months of life.

Table 1. Sample Tracking Definitions

Sample tracking measure	Data Source(s)	Definition
Index pregnancy	Birth Certificates, fetal death records, Medicaid claims and eligibility, hospital discharge	Having matched administrative records for one or more of the outcomes listed below during the period of the index pregnancy. This period is defined as covering the weeks of gestation provided in the obstetric estimate on the vital record until the date of birth or fetal death, when a mother has a matched index birth or fetal death record. In the absence of an index birth or fetal death, we define the pregnancy period as the time covering 42 weeks prior to the expected delivery date provided on the baseline survey.

[v] Specified in McConnell et al. (2020) as a death of the fetus occurring at or after 20 weeks of gestation.

		● A live birth certificate for the index birth,<br>● A Kuklina identified live birth in Medicaid claims or hospital discharge records occurring within 120 days of the expected delivery date<br>● A probable pregnancy loss in Medicaid claims or hospital discharge occurring during the index pregnancy<br>● A fetal death record in Vital Records occurring during the index pregnancy<br>● An antenatal care visit in Medicaid claims occurring during the index pregnancy<br>● Enrollment in Medicaid for pregnant women overlapping with the index pregnancy period
Index birth	Baseline survey, birth certificates	A birth identified by a matched birth certificate in Vital Records with a date of birth within 120 days before or after the estimated delivery date reported on the baseline survey.

Table 2. South Carolina Medicaid income eligibility criteria as a percent of the Federal Poverty Line over the study period

	Jan 2016	Jan 2017	Jan 2018	Jan 2019	Jan 2020	Jan 2021
Pregnant women[2,3]	199%	199%	199%	199%	199%	194%
Parents[4,5]	67%	67%	67%	67%	67%	95%
Children 0-1[3,6]	213%	213%	213%	213%	194%	208%
Children 1-5[3,7]	213%	213%	213%	213%	143%	208%
Children 6-18[3],[8]	213%	213%	213%	213%	133%	208%

A.2. Characterizing missing data

We may be unable to track mother and child outcomes for pregnant women enrolled in our trial over the entire period spanning pregnancy and the child's first two years of life for various reasons. First, we may be unable to match the identifying information provided by women at study enrollment to administrative records because of poor data quality or because women enrolled in the trial moved out of state. Second, we may be unable to match women to their index birth because of problems linking mother and child records within South Carolina's administrative record system or because their pregnancy ended in pregnancy loss. We may be unable to observe child outcomes through 24 months if a child dies before reaching 24 months of age. For outcomes that can only be observed in Medicaid claims data, such as child preventative health outcomes and outpatient care utilization for mothers, we will not observe outcomes for mothers or children not enrolled in Medicaid. Whenever possible, we will report missing outcome data and characterize missing outcome data across each of these groups. More details are provided in Addendums 1-3 regarding plans to characterize missing outcome data related to each of the three domains of potential program impact and plans for sensitivity analyses to account for potential patterns in missing outcome data.

Because administrative records update continuously over time, we anticipate that mothers and babies classified as falling within our study sample definitions may change as administrative data is updated. For example, a mother enrolled in the trial whom we cannot initially match to any records may later match to state Medicaid records. Furthermore, administrative data may experience delayed reporting or data errors that are corrected later. We may revisit analyses several years after completion to assess the robustness of results to updated administrative records.

B. Analytical considerations

B.1. Control variables

In addition to reporting unadjusted models of program impact, we plan to report models that adjust for characteristics at baseline that may be associated with our primary academic outcomes. These include participant age, race and ethnicity, gestational age at study enrollment, relationship with the father of the child, education, employment, use of social services, housing stability, health care utilization, health behaviors and physical and mental health status all measured during the baseline survey. Specific variable definitions that we plan to include as control variables are specified below. Binary variables will be coded as “1”, “0”, or missing; for coding categorical variables, we will consider distributions across the control and treatment groups including missing values. We plan to use the same set of control variables in all analyses.

- Implementing Agency
    - Indicators for each implementing agency enrolling study participants

- Demographics:
    - Indicator for age equal to 15, 16, or 17
    - Indicator for age equal to or greater than 28
    - Race – indicators for non-Hispanic Black, non-Hispanic white, other
    - Ethnicity – indicator for Hispanic/Latina
- Gestational age at time of study enrollment
    - A continuous variable of weeks to delivery as calculated by difference in estimated due date and the survey date
- Relationship with father of the child
    - Indicator for daily interaction with father of the child
- Education
    - Indicator for high school diploma or GED with no higher education
    - Indicator for less than high school diploma
- Employment, Income, and Financial Resources
    - Indicator for whether the mother is working for pay at the time of the survey
- Social services
    - Indicator for receiving one or more social service (i.e. TANF, SNAP, SSI, unemployment benefits, and WIC)
- Housing stability
    - Indicator for moving two or more times in the previous twelve months
    - Indicator for living with parents
- Access and utilization of health care, including mental health and maternal health
    - Indicator for receiving at least one antenatal care visit before time of survey
    - Indicator for having obtained care at a hospital ER in past six months
    - Indicator for receiving mental health treatment in the previous year
- Health behavior (e.g. drinking and smoking)
    - Indicator for reporting having consumed alcohol in the three months before pregnancy
    - Indicator for reporting having smoked cigarettes in the three months before pregnancy
- Psychological state/resources (measured on a PHQ-2 scale)
    - Indicator for PHQ-2 score of 3 or higher.
- Baseline measure of self-reported health
    - Indicator for self-reported health described as fair or poor.
- Maternal perceived stress level
    - Indicator for PSS-4 score of 4 or higher.
- Self-reported pregnancy risk factors
    - Indicator for pre-pregnancy weight and height yielding BMI of normal.

- Family planning
    - Indicator for responding yes to having access to a place for family planning or birth control
    - Indicator for responding yes to wanting more children one day

B.2. Missing values of control variables

We will treat baseline responses as missing if sample members responded to questions with "Don't know" or "Refused to Answer." We will use the dummy-variable adjustment method to account for missing baseline covariates in our analysis.[9] Specifically, we will set missing covariate values to a constant value and add indicators (i.e., dummy variables) for missing values to the impact analysis model. We will also assess our results' robustness to a model specification without baseline covariates. Finally, based on the prevalence and patterns of missing covariate data, we may specify a multiple imputation model to impute missing values.

B.3. Alternate Empirical Specifications

Per McConnell et al. (2020), we will estimate intent-to-treat (ITT) effects as our primary empirical specification.[1] We may also consider secondary specifications that incorporate data on actual program participation, taking advantage of the randomization as an instrument for participation to estimate local average treatment effects and to examine average characteristics of those participating in the program. We anticipate that the primary alternative definitions of interest for program participation will be based on reaching the milestone of participating in home visiting through the period surrounding the expected delivery date, the child's first birthday and the child's second birthday. We would operationalize participation through the expected delivery date and the child's first birthday as mothers who receive a nurse visit within 14 days of the milestone. Participation through the child's second birthday would be operationalized as a visit within 1 month (31 days) of the milestone. In accordance with the NFP program model, mothers should receive a visit approximately once every two weeks in the period leading up to delivery through the first 21 months of the child's life and should receive a visit once every month in months 21-24 of the child's life.

Nurse-Family Partnership Evaluation Analysis Plan Addendum 1: Pregnancy, Birth and Maternal Health Outcomes.

Note: This version of Addendum 1 reflects plans for study design as of January 20, 2021. The first unblinding is planned for January 21, 2021 when interim outcomes will be analyzed for Pay-for-Success payments.

The goal of this analysis plan addendum is to enumerate analysis specific to the pregnancy, birth, and maternal health outcomes domain.

1. Methods

A full treatment of our empirical approach, primary study outcomes, planned subgroup analyses, and statistical power is given in sections 2.4-2.10 of McConnell et al. (2020).[1] The following sections provide additional context and details on planned analyses for the pregnancy, birth, and maternal health outcomes domain of the NFP evaluation.

1.1. Defining the Sample for Analysis

For analysis of our primary adverse birth outcome, we will consider our primary sample to be mothers who experience either a fetal death or index birth (defined in section A.1 of the pre-analysis plan). For analysis of other outcomes, our primary sample will be mothers with an index birth, as many outcomes will not be observable in the case of fetal death.

1.2. Construction of Study Outcomes

We use a combination of South Carolina vital records, Medicaid claims, and hospital discharge records to construct study outcomes. In cases where outcomes differ between records, (e.g. gestational age at delivery, birthweight) we will use estimates from South Carolina vital records.

1.2.1 Primary Outcome

Our primary outcome for this domain is an adverse birth outcome, which we define as having a preterm birth (less than 37 weeks' gestation), a newborn being small for gestational age (less than 10th percentile of US births conditional on gestational age), having low-birth weight (less than 2500 grams), or experiencing perinatal mortality (fetal death occurring at or after 20 weeks' gestation or mortality in the first 7 days of life). Data for the adverse birth outcome will come from South Carolina birth records, death records, and fetal death records.

For mothers with multiple births, we define the outcome based on having an adverse birth outcome for any child from the index pregnancy. While preterm birth and other adverse birth outcomes may be more common among multiple births, we anticipate that rates of multiple births will be equal across treatment and control arms. We may also explore alternative specifications of this outcome that include only singleton births.

1.2.1 Secondary Outcomes

We will examine several secondary infant and maternal outcomes. For infant outcomes, we will examine each adverse birth outcome individually (SGA, preterm, low birth weight, perinatal mortality). We will also examine large-for-gestational age (>90th percentile of US births conditional on gestational age), very low birth weight (<1500g), a continuous measure of birth weight, and extremely preterm (<28 weeks gestation) using birth certificate records. We will also examine neonatal morbidity, defined as assisted ventilation immediately after delivery, assisted ventilation for more than six hours, seizure, receipt of surfactant replacement therapy, or receipt of antibiotics for suspected sepsis using data from the birth certificate.[vi,10,11] **Finally, we will examine NICU admission of at least overnight, defined as a claim with a procedure code of 99468, 99469, 99477, 99478, 99479, or 99480 on the day of delivery and also on the following day.**

We will also examine several maternal health related secondary outcomes at birth, including cesarean delivery (from the birth certificate) and severe acute maternal morbidity from hospital discharge and Medicaid data (as defined by the Centers for Disease Control and Prevention).[12] We will examine maternal mortality up to one year after birth using vital records. We will examine receipt of any postpartum visit within the first 12 weeks postpartum using Medicaid claims data, defined as a claim with a diagnosis code of Z39 or a HCPCS code of 59430.

Next, we will examine the outcome of neonatal abstinence disorder and/or maternal drug/substance abuse both during the index pregnancy period and over the period spanning the index pregnancy and the first 24 months following the index birth. This outcome is defined as a composite outcome. While the outcome is only specified over the longer time-period in our protocol and trial registry, exploratory analysis of this outcome during the index pregnancy will be valuable because of the strong relationship between drug use and birth outcomes.[13] We will define the composite outcome related to substance abuse as any record in the Medicaid claims or hospital discharge data with a code listed in Table 1.1. We will also examine robustness to a definition in which we exclude the tobacco codes which we expect to make up the majority of claims we observe matching to maternal substance use.

Next, we will examine maternal experience of violence or homicide during the index pregnancy and during the first 24 months after the index birth using a combination of death records and Medicaid and hospital discharge data. This is defined as any record matching a code listed in

---

[vi] In our study protocol (McConnell et al 2020) we indicated that we planned to derive neonatal morbidity from discharge records. However, in order to make our analyses parallel with other analyses looking at neonatal morbidity (Han et al 2020) and because of concerns about the ability to translate these concepts directly into claims data (Ford et al 2007), we plan to rely instead on birth certificate data.

Table 1.2. As above, experience of violence was originally specified over 24 months, but we will explore analysis of this outcome during the index pregnancy because of the negative associations between exposure to maternal violence and birth outcomes.[14] Our current definitions of substance abuse and maternal experience of violence are limited to the codes defined here; however, we will explore incorporating broader indicators of challenges with substance abuse derived from pharmacy claims, child claims, and criminal justice records. We will update these outcome specifications prior to any analysis of comparisons across treatment and control groups for these outcomes.

We will also examine use of social services during pregnancy and during the first 24 months after delivery. During pregnancy, we will examine any receipt of WIC benefits (derived from the birth certificate) and any receipt of Supplemental Nutrition Assistance Program (SNAP) benefits (from South Carolina Department of Social Services data). During the first 24 months after delivery, we will examine total months of receiving SNAP benefits and Temporary Assistance for Needy Families (TANF) benefits, as well as a measure of benefit churn, defined as receiving SNAP or TANF benefits at any time during a given year and having experienced at least one break in participation of four months or less that started and/or ended during the year.[15]

Table 1.1. Codes for composite outcomes related to neonatal abstinence disorder and maternal drug/substance abuse

Code Description	ICD-10 Code(s)
Neonatal abstinence disorder	P96.1, P04*
Opioids	F11.*
Tobacco	F17.2*, O99.33*, Z72.0
Alcohol	V11.3*, F10.*
Sedative	F13.*
Cocaine	F14.*
Amphetamines	F15.*
Cannabis	F12.*

Source: Jarlenski et al. 2020[16]

Table 1.2. Codes for maternal experience of violence

Code Description	ICD-10 Code(s)
Adult neglect	T74.01; T76.01
Adult physical abuse	T74.11; T76.11
Adult sexual abuse	T74.21; T76.21
Adult psychological abuse	T74.31; T76.31
Unspecified adult maltreatment	T74.91; T76.91

Husband, perpetrator of maltreatment	Y0701
Assault by unarmed brawl	Y040
Observation after rape	Z0441
Assault by other bodily force	Y048
Unspecified perpetrator of maltreatment<br>Other family member perpetrator of maltreatment	Y079<br>Y07499
Encounter for mental health services for victim of spousal or partner abuse	Z691x
Encounter for mental health services for victim or perpetrator of other abuse	Z698x
Encounter for observation following alleged adult physical abuse	Z0471

Sources: Crosswalk of ICD-9 to ICD-10 codes from Davidov et al. 2017 and Schafer et al. 2008.[17,18]

<u>Antenatal Care Utilization & Quality</u>

Analyses of outcomes related to antenatal care utilization and quality is planned as a stand-alone analysis. We will examine three groups of outcomes related to antenatal care utilization and quality: utilization of health care services during pregnancy, guideline-recommended antenatal care, and indicators related to antenatal health. We list the outcomes that fall under each area, and their associated data sources, below. We also include the CPT/HCPCS, NDC (National Drug Code) and ICD-10 codes used to identify the outcomes that use Medicaid claims or hospital discharge data in Table 1.3.

The primary sample for this analysis will be mothers with an index birth. We will use vital records, hospital discharge, and Medicaid claims data for the analyses. Mothers who do not match to one of these data sources will not be included in the sample for the corresponding outcomes (e.g., mothers who do not match to Medicaid claims will not be included in the analyses of guideline-recommended antenatal care).

<u>Utilization of health care services during pregnancy</u>

- Adequacy of Prenatal Care Utilization Index (APNCU) [vital records data]
- Number of emergency department visits during pregnancy [all-payer hospital discharge data]
    - To better understand the effect of NFP on emergency department utilization, we may also examine treatment vs. control differences in emergency department use by trimester of the emergency department visit and by the enrollee's overall adequacy of prenatal care (APNCU).
- Number of ultrasounds during pregnancy [Medicaid claims data]

- Consultation with maternal fetal medicine specialist [all-payer hospital discharge data]
- Dental visit during pregnancy [Medicaid claims data]

Guideline recommended antenatal care

- Anatomy scan (between 18-22 weeks' gestation) [Medicaid claims data]
- Gestational diabetes test (between 24-28 weeks' gestation) [Medicaid claims data]
- TDAP vaccine (between 27-36 weeks' gestation) [Medicaid claims data]
- Group B strep test (between 35-38 weeks' gestation) [Medicaid claims data]

Antenatal health

- Smoking cessation during pregnancy [vital records data]
    - We will consider an enrollee to have ceased smoking during pregnancy if their vital record indicates that they smoked pre-pregnancy and that they did not smoke during pregnancy.
- Recommended gestational weight gain [vital records data]
    - Gestational weight gain will be classified as "recommended" if it is within the guidelines published by the National Academy of Medicine (formerly the Institute of Medicine).[19]

The guideline-recommended antenatal care services should be received at the appropriate gestational age during the pregnancy. The gestational age at the time of the test will be calculated as the date of the test minus the approximate date of last menstrual period (in days). We will approximate the enrollee's date of last menstrual period using the child's date of birth and obstetrician's estimate of gestational age at delivery from the vital records. If these fields are missing, we will define the approximate date of last menstrual period as the beginning date of the index pregnancy (defined above).

Each of these outcomes can only be measured among pregnancies that reach the gestational age at which the care is recommended. The main analyses will only include pregnancies that last at least as long as the gestational age recommended for each outcome. However, it is possible that NFP has a direct effect on gestational age, which would mean that the sample of mothers observed could differ across treatment and control arms. If that is the case (i.e., if gestational age at birth between treatment and control group is statistically significantly different), then we will conduct a bounding exercise as a robustness check for the guideline-concordant care outcomes.[20] Using Group B Strep as an example, the main analysis will compute the average treatment effect (ATE) among people whose pregnancies last at least 35 weeks. If gestational age is statistically significantly different between control and treatment arms, we will estimate two bounds on this ATE: (1) a recalculated ATE assuming *all* people who did not reach 35 weeks'

gestation would have received the test, and (2) a recalculated ATE assuming *none* of the people who did not reach 35 weeks' gestation would have received the test.

Table 1.3. Codes for analyses of antenatal care utilization and quality

	CPT/HCPCS	NDC	ICD-10
Dental visit	D1110, D0120, D0140, D0150, D0160, D0170, D0191, D1206, D1208, D02*, D0330, D0340, D0350, D2391, D2392, D2393 D1351, D71*, D7210, D7220, D7230, D7240, D7241, D7250		
Ultrasound	76801, 76805, 76811, 76813, 76815, 76816, 76817, 76818, 76819, 76810, 76812		
Anatomy scan ultrasound (18-22 weeks)	76805, 76811, 76815, 76816, 76817, 76810, 76812		
Gestational diabetes test (24-28 weeks)	82950, 82951, 82947		
TDAP vaccine (27-36 weeks)	90696, 90697, 90698, 90700, 90701, 90714, 90715, 90471, 90472, 90460, 90461	49281040010, 49281040015, 49281040020, 58160084211, 58160084252	
Group B streptococcus test (35-38 weeks)	87150, 3294F, 87802, 87653, 87801, 87081, 87084, 87070, 87077, 87147		Z36.85

Maternal utilization of mental health services

Analyses of outcomes related to maternal utilization of mental health services is also planned as a stand-alone analysis. NFP guidelines require nurses to screen for depression and anxiety at pre-specified intervals throughout the program, and to follow their agencies' protocols for referral and care coordination for women who screen positive. While directly measuring the prevalence of depression and anxiety in our study sample would help us assess the impact of NFP on mental

health, our study design relies exclusively on administrative data sources for outcome measurement. Therefore, our study design will seek to analyze how NFP changed the utilization of mental health services during the perinatal period.

We include all women with an index birth in our analytic sample, regardless of whether they had a prior mental health diagnosis before pregnancy. Because few women in our study are enrolled in Medicaid prior to pregnancy, we cannot observe prior diagnoses or identify treatment initiation. However, we expect rates of prior mental health diagnoses before pregnancy to be equal across treatment and control arms. Because the outpatient mental health treatment outcomes rely exclusively on Medicaid claims, we will restrict the analytic sample to those who retain full Medicaid coverage through 60 days postpartum. We will not restrict the sample for inpatient mental health outcomes which rely on utilization that will be observable in all-payer hospital discharge data.

While the medical definitions for depression and anxiety are distinct, we examine them together because they are comorbid conditions[21,22] and NFP screens for them simultaneously. We define outpatient mental health treatment as a composite outcome of either a diagnosis for depression/anxiety/stress reaction or a filled prescription for antidepressants/anxiolytics or an outpatient psychotherapy visit during pregnancy or the first 60 days postpartum. We list the ICD-10 diagnosis codes, therapeutic class codes, and Current Procedure Terminology (CPT) codes used to define these outcomes in Tables 1.4, 1.5, and 1.6. Consistent with prior research[23], we consider all outpatient psychotherapy visits, including individual, family or group therapy to be mental health visits.

Table 1.4. Diagnosis codes for depression/anxiety/stress reaction

Code description	ICD-10 Code(s)
Depression	F53; O906; F99; O9934*; F32*; F33*
Anxiety	F41.*
Stress-reaction	F43.*

Table 1.5. Therapeutic class codes for filled antidepressants or anxiolytics

Code description	Therapeutic class codes
Antidepressants	281604
Anxiolytics	2824*

Table 1.6. Outpatient psychotherapy codes

Code description	CPT codes
Psychotherapy (individual, family or group)	90804-90815; 90832-90834; 90836- 90840; 90845-90847; 90849; 90853; 90857; 90862; 90875; 90876

We will also look at these outpatient measures individually (i.e., diagnosis, filled prescription, and psychotherapy) and in combination to enhance our understanding of treatment patterns. For example, we may be interested in the proportion of mothers who receive a diagnosis but do not fill a prescription or receive psychotherapy as this could signal structural or individual-level barriers to treatment.[23] We may also be interested in the proportion of women who receive medication only, as psychotherapy is often recommended as a first-line treatment for mild or moderate depression and in combination with medication when the depression is severe.[24] We may also explore the timing of outpatient mental services (measured in days). NFP could lead to earlier treatment of perinatal depression/anxiety through regular screening, coordinating care, providing a warm hand-off, or by reducing the stigma surrounding mental health and treatment.

In our clinical trials registry, we defined a measure of mental health treatment follow-up care as: a second antidepressant prescription or outpatient mental health visit within 120 days of the initial treatment ("acute phase"). However, differential Medicaid coverage rates between the treatment arms after 60 days postpartum may preclude us from measuring the treatment effect for this outcome reliably. For example, if treatment group members are more likely to be enrolled in Medicaid after 60 days postpartum than control group members, observed differences in mental health treatment patterns between the treatment and control groups could be due to group differences in the composition of who remains on Medicaid. Plans for assessing whether attrition from Medicaid affects the comparability of the treatment and control groups are described in Section 1.5, Endogeneity of data sources and sensitivity analysis.

For inpatient mental health outcomes, we will use hospital discharge data to measure mental health related visits on both the extensive and intensive margins. Specifically, we will create an indicator for any mental health related emergency or inpatient visits during pregnancy or the 12 months postpartum based on all-listed diagnoses (i.e. primary or secondary) for depression/anxiety/stress reaction, excluding inpatient claims on the day of delivery. We will also create a count variable for the number of mental health related emergency or inpatient visits during pregnancy or the 12 months postpartum based on all-listed diagnoses for depression/anxiety/stress reaction, excluding inpatient claims on the day of delivery. We

examine these hospital-related outcomes because public insurance pays for six of every ten inpatient stays related to mental health,[25] and they could indicate inadequate outpatient treatment for perinatal depression and anxiety. We may also look at diagnoses for depression/anxiety/stress on the day of delivery, as prior research has found that a large proportion of women have diagnoses on this day.[23] These diagnoses may represent cases where women had subclinical or previously unidentified symptoms during pregnancy that were identified during the hospital stay.[23] Finally, we may construct an outcome that captures the proportion of women who have an emergency or inpatient stay related to mental health without any medication or outpatient psychotherapy treatment during pregnancy or the 12 months postpartum.

1.3. Timing of Study analyses

We will wait to complete this analysis until all study births have taken place and have completed administrative records which have been sent to the study team and matched to existing analytic files. We will first publish our analysis of the primary outcome (described above) together with secondary outcomes related to maternal and infant health that can shed light on understanding impact estimates for the primary outcome. Following the publication of those results, we plan to produce separate analyses exploring the impact of NFP on maternal and infant outcomes related to the period of childbirth and the postpartum period, antenatal care and mental health care utilization. At 24 months, we plan to examine women's substance abuse, experience of violence, and use of social services, as described above. We may also examine mechanisms of these outcomes, for example, via nurse referrals. If Medicaid coverage is not differential between treatment and control group mothers at 24 months postpartum and the groups remain comparable, we may also explore outpatient mental health treatment patterns for up to two years after birth since the trajectory of depression symptoms in the postpartum period is increasingly understood to vary widely.[26,27] Below we describe our plans for assessing attrition, missing outcome data and possible endogeneity arising from our data sources.

1.4. Attrition and Missing Outcome Data

For the purposes of tracking our sample, we will separately describe the share of mother-baby dyads who fall in three distinct categories where we may observe an index pregnancy, but no index birth or fetal death as follows:

1. *Probable loss of pregnancy,* which is defined as the absence of a matched birth certificate for the index birth and either (a) a matched birth certificate for a subsequent pregnancy where the birth record indicates the mother has had 0 previous live births or (b) the presence of a matched Medicaid claim or hospital discharge record containing a diagnosis or procedure code indicating a probable pregnancy loss during the index

pregnancy. Below are the validated ICD-10 diagnosis codes that we use to identify probable pregnancy losses (Table 1.7).

Table 1.7. Probable pregnancy loss diagnosis codes

ICD-10-CM Code	Code Description
O02.0-O03.9	Spontaneous abortion
O00	Ectopic pregnancy
O01	Molar pregnancy

2. *Mother-baby linking error* is defined as the absence of a matched birth certificate for the index birth despite a matched record for the index birth in either Medicaid claims or hospital discharge records. The matched record in Medicaid claims or hospital discharge data must have an admission date within 120 days of the estimated due date reported on the baseline survey and an ICD-10 diagnosis code indicating a live birth according to ICD-9 claims identified by Kuklina et al. (2008) and widely used in claims-based research on birth outcomes.[28]
3. *Unmatched pregnancy outcome* which is defined as the absence of an administrative record for a birth or pregnancy loss episode for the index pregnancy in vital records, Medicaid claims, and hospital discharge data. Some of these "unmatched" cases may eventually become probable pregnancy loss if mothers later match to a birth certificate reporting a first-time birth.

We will report rates of missingness of outcome data for each category listed across the full sample and by treatment arm in order to characterize sample attrition and identify differential attrition across treatment arms in each of these categories.

1.5. Endogeneity of Data Sources and Sensitivity Analysis

Among the mothers in our study who experience a fetal death or live index birth, a small percentage will have an error linking the mother's records to their child's vital records. Our primary sample of mothers will focus on those with fetal deaths or index births observed in vital records, because these records contain more complete data on birth outcomes. We will also perform a robustness check that utilizes the sample of mothers with a fetal death or index birth observed in either vital records, Medicaid claims or hospital discharge records. In cases where a vital record exists for the index pregnancy, we will calculate the adverse birth outcome in the manner described in section 1.1.

1.5.1 Calculating Adverse Birth Outcomes Without Vital Records Data

In this section, we define the calculation of the adverse birth outcome in instances where there is a matched record for the index birth or fetal death in hospital discharge record or Medicaid claims, but not in vital records.

We identify births in hospital discharge records and Medicaid claims using the ICD-10 diagnosis code definition of live birth in Kuklina et al. (2008) and listed in Table 1.8.[7] We will use the ICD-10 diagnosis code Z3A to calculate gestational age. In cases where an ICD-10 code for gestational age is absent from the hospital or Medicaid claim record, we will calculate gestational age at birth using the admission date on the record and the expected due date as given in the baseline survey. The preterm birth component of the adverse birth outcome will be coded as 1 if the gestational age on the health record is less than 37 weeks, or, if the record contains a diagnosis code P07.30-P07.39. The low-birth weight component will be coded as 1 if the record contains a diagnosis code P07.0-P07.18. The SGA component will be coded as 1 if the record contains a diagnosis code of P051. Fetal deaths will be identified with the codes in Table 1.9 when accompanied by an ICD-10 diagnosis code Z3A for gestational age of at least 20 weeks.

Table 1.8. Codes to Identify Live Births

ICD-10 Code(s)	Code Description	Exclusion Code
O80, O82	Encounter for delivery	No
Z37	Outcome of delivery	No
10D00Z0, 10D00Z1, 10D00Z2	Extraction of products of conception	No
10D07Z3, 10D07Z4, 10D07Z5, 10D07Z6, 10D07Z7, 10D07Z8	Forceps/Vacuum/Breach	No
0W8NXZZ	Episiotomy	No
10A07ZZ, 10A00ZZ, 10A03ZZ, 10A04ZZ, 10A07ZX, 10A07ZW, 10A07Z6, 10A08ZZ	Abortion of products of conception	Yes
O019	Hydatidiform mole, unspecified	Yes
O048	Shock following (induced) termination of pregnancy	Yes
O02	Missed abortion	Yes
O00	Tubal pregnancy without intrauterine pregnancy	Yes
O03	complete or unspecified spontaneous abortion without complication	Yes
O08	Complications following ectopic and molar pregnancy	Yes

Table 1.9. Fetal Death Codes

ICD-10 Code(s)	Code Description	Exclusion Code
Z37.1	Single stillbirth	No
Z37.4	Twins, both stillborn	No
Z37.7	Other multiple births, all stillborn	No
P95	Stillbirth on child claims (occasionally appears on Mother claims)	No
O36.4	Maternal care for intrauterine death	No

1.6. Subgroups

Our primary sub-group is described in the study protocol. Briefly, this subgroup comprises women who have an indicator of poor mental health, are under 19 years of age, or have not completed high school/received a General Education Development (GED) certificate by the time of enrollment.

In addition to the planned subgroup described above, we may also consider other potential subgroups of particular importance for outcomes related to pregnancy, childbirth, and maternal health. In particular, we may examine the program's impact on the outcomes of Black mothers who are disproportionately at risk for adverse pregnancy and birth outcomes compared to white mothers[29,30] and who receive postpartum mental health care at disproportionately lower rates.[31] We are also interested in focusing on births where the period of pregnancy and birth occurred prior to the start of the COVID19 pandemic (defined here as March 23, 2020). We expect that the pandemic altered NFP program implementation, clinical practices and utilization of care surrounding pregnancy, as well as families' mental health.[32]

1.7. Accounting for Multiple Hypothesis Testing

For our measures of guideline-recommended antenatal care, we will report a summary index of the outcomes alongside the individual outcomes to be more parsimonious in the number of hypotheses we are testing. The summary index of the four guideline-recommended care outcomes will equal the proportion of the services that the enrollee received. For example, the index will be equal to 0.5 if they received two out of the four services, and equal to 1 if they received all four services.

Nurse-Family Partnership Evaluation Analysis Plan Addendum 2: Child Health Outcomes

Note: This version of Addendum 2 reflects plans for study design as of January 20, 2021. The first unblinding is planned for January 21, 2021 when interim analyses will be performed for Pay-for-Success payments.

The goal of this analysis plan addendum is to enumerate analysis specific to the child health outcomes.

2. Methods

A full treatment of our empirical approach, primary study outcomes, planned subgroup analyses, and statistical power is given in sections 2.4-2.10 of McConnell et al. (2020).[1] The following sections provide additional context and details of planned analyses for child health-related outcomes.

2.1. Defining the Sample for Analysis

Our sample for analysis of our primary child-health related outcome will be restricted to mother-baby dyads with an identified index birth as defined in section A.1 of the pre-analysis plan. All analysis will take place at the mother level, where randomization occurs. We discuss how outcomes will be operationalized in the case of multiple births below. We will not restrict the sample based on matching to Medicaid data as the outcomes include utilization that will be observable in all-payer hospital discharge data. For outcomes that rely primarily on Medicaid data (i.e. outcomes related to preventive child health utilization), we may restrict the sample to mother-baby dyads where children retain either partial or continuous Medicaid coverage through the child's first or second year of life. We anticipate that the children of most mothers who enroll in the trial will be eligible for Medicaid for their entire first year of life but we anticipate that more children may drop off of Medicaid during their second year of life, whether because of the need to document eligibility or because of changes in eligibility in the second year of life during some periods of the study (see Table 2). We discuss the potential differences in Medicaid enrollment between control and treatment arms and planned sensitivity analyses and alternative specifications in section 2.4.

2.2. Construction of Study Outcomes

For our primary study outcome, we will assess the likelihood of experiencing injury, abuse, or neglect during early childhood. This outcome will be defined as a composite measure indicating a health care encounter or mortality associated with International Classification of Diseases (ICD) codes indicating either a major child injury or suspicion of abuse or neglect. We will identify major injury as any medical claim or mortality case that includes an ICD code associated with injury excluding superficial injuries, injuries related to medical care, and injuries stemming from allergic reactions. ICD codes indicating suspected abuse or neglect are derived from validated

methods described in Schitzer et al. 2011 and Hooft et al. 2013.[33,34] Data on early childhood injury outcomes and suspected abuse or neglect will come from South Carolina all-payer hospital discharge records, Medicaid inpatient and outpatient claims and mortality records. Secondary outcomes related to injuries, suspected abuse or neglect and mortality will decompose the primary outcomes into their composite parts. In the case of multiples, we will consider a mother to experience the outcome if any of her children do and we will average the number of events in cases where the outcome is continuous (i.e. the number of injuries).

2.2.1 Secondary Outcomes

We specify the diagnosis and procedure codes used to measure the utilization of preventative care for children under 2 in Table 2.1. We will construct these outcomes using Medicaid claims data. We define receiving the share of recommended well-child visits as a binary indicator equal to one if a child has received at least the number of well-child visits in the first 2 years of life as recommended by the American Academy of Pediatrics.[35] In the case of multiples we will consider binary outcomes to have occurred if all children from the index birth meet the criteria.

Table 2.1. Preventative Care Utilization Measures

Outcome	Outcome Definition	Code(s)
Well-child visits	Binary indicator equal to 1 if the child has received 9 well-child visits by 24 months	ICD-10: Z00.1; CPT: 99391, 99392
Lead screening	Received at least one lead screening by 24 months	ICD-10: Z13.88; CPT: 83655
Developmental screening	Received at least one developmental screening by 9 months	ICD-10: Z13.41, Z13.42; CPT: 96110
Dental visit	Received at least one dental visit by 12 months	NA
Fluoride treatments	Binary indicator equal to 1 if the child has received at least one fluoride treatment by 24 months[vii]	ICD-10: Z293; CPT: 99188

[vii] While our protocol paper specified this outcome as receiving at least four fluoride treatments, there are inconsistent guidelines regarding the recommended number of visits. Therefore, we have modified the definition of this outcome to be a binary variable indicating whether any fluoride treatments were received. See https://www.uspreventiveservicestaskforce.org/uspstf/recommendation/dental-caries-in-children-from-birth-through-age-5-years-screening for details on fluoride varnish recommendations.

2.3. Timing of study analyses

We will complete analysis of the primary outcome and related secondary outcomes once all study births have occurred, and at least 24 months have elapsed following the study birth. We will add additional lags to account for the time it takes for administrative records to be complete, sent to the study team, and matched to existing analytic files. If we make additional changes to the analysis plan before starting the analysis, we will indicate those changes with a new version number and date. We plan to publish analyses of the primary outcome and related secondary outcomes first. A separate manuscript is planned to explore outcomes related to preventative health care utilization for children. We also plan to conduct follow-up analyses of similar outcomes as children age, including beyond the time when families would participate in NFP. Analytical plans for follow-up longitudinal analyses will be added as they are developed.

2.4. Attrition and Missing Outcome Data

We will report on the following categories of mother-baby dyads for whom we may not be able to see outcomes related to child health, reporting on the sample of children tracked to 12 months and 24 months separately:

a. *Mortality prior to 12 or 24 months of age:* We will use vital records to identify children born into the study who experience mortality prior to the age of 12 months.
b. *Unmatched to Medicaid Eligibility through 12 or 24 months of age:* We will consider children who never match to Medicaid eligibility in any of their first 12 months of life or 13-24 months of life respectively.

We will report rates of missingness for each category listed here for the whole sample and by treatment arm in order to characterize missing outcome data and identify differential rates of missing outcome data across treatment arms in each of these categories.

2.5. Endogeneity of Data Sources and Sensitivity Analysis

As discussed above, because child preventative health outcomes will be observed exclusively in Medicaid claims data, we will not observe these outcomes among children who are not enrolled in Medicaid. Because NFP may affect maintenance of Medicaid coverage through the child's first two years of life, child enrollment rates in Medicaid may differ between control and treatment groups.

We will conduct several analyses to determine whether rates of enrollment in Medicaid differ between treated and control groups. First, we will compare enrollment through the first year of life and the first two years of life across treatment and control groups. Even if we do not observe differences in Medicaid enrollment rates between the two groups, the type of children retaining coverage may be influenced by participation in NFP. Therefore, we will also test whether there

are differences in the characteristics of participants between control and treatment groups over the two time periods (12 months and 24 months). We will use a joint F-test to compare characteristics measured at baseline across these two groups using the list of baseline covariates specified in section B.1.

When conducting study analyses, we will divide outcomes between those that we can observe within the first year of life and those measured over the first two years of life. **As specified in our study protocol in Section 2.12,** [1] **we will report our primary outcome excluding data that comes from Medicaid to assess the robustness of our results to this potential source of endogeneity.** If we observe significant differences in Medicaid enrollment across treatment and control groups in the second year of life, we may focus more on outcomes that can be measured within the first year of life.

2.6. Subgroups

Our primary sub-group is described in the study protocol. Briefly, this subgroup is composed of women who report mental health challenges, are under 19 years of age, or have not completed high school/received a GED certificate by the time of enrollment.

In addition to the planned subgroup described above, we may also consider other potential sub-groups of particular importance for outcomes related to child health. In particular, we are interested in focusing on child outcomes where the first year of the child's life was completed prior to the start of the COVID-19 pandemic (defined here as March 23, 2020). We expect that the pandemic altered the way that the NFP program was implemented. In addition, it is likely that the pandemic substantially affected clinical practices and utilization of pediatric care.

2.7. Accounting for Multiple Hypothesis Testing

We have planned for the analysis of several outcomes related to utilization of preventative care for children. To account for multiple related secondary outcomes in the domain of preventative care we will construct an index of preventative care-seeking, similar to the approach proposed by Kling, Liebman and Katz 2007.[36] We will report the index in addition to other outcomes.

Nurse-Family Partnership Evaluation Analysis Plan Addendum 3: Alter Maternal Life Course

The goal of this analysis plan addendum is to enumerate analysis specific to the outcome domain

Note: This version of Addendum 3 reflects plans for study design as of January 15, 2021. The first unblinding is planned for January 18th, 2021 when outcomes will be analyzed for Pay-for-Success payments.

focused on birth spacing and family planning utilization.

3. Methods

A full treatment of our empirical approach, primary study outcomes, planned subgroup analyses, and statistical power is given in sections 2.4-2.10 of McConnell et al. (2020).[1] The following sections provide additional context and details on auxiliary analysis for the outcomes related to altering the maternal life course including birth spacing and family planning utilization.

3.1. Defining the Sample for Analysis

The sample will be restricted to women with a fetal death or index birth as defined in section A1 of the pre-analysis plan.

3.2. Construction of Study Outcomes

We outline the construction of study outcomes across two domains: outcomes related to birth spacing and outcomes related to the utilization of contraceptive methods.

3.2.1. Construction of birth spacing outcomes

Data will be obtained from vital statistics birth records. In the case of multiples, birth spacing will be measured as the time between the date of birth of the last child from the index pregnancy to the date of birth of the first child from the subsequent pregnancy. There may be cases where the subsequent birth is implausibly close to the index birth. Births that occur less than or equal to 90 days from the index birth will be assumed to be multiple gestation based on guidelines provided by the National Center for Health Statistics. Subsequent births that occur between 90 days and 21 weeks (147 days) after the index birth will be considered outliers and will be dropped from the analysis. We define birth outcomes of interest in Table 3.1.

Table 3.1. Birth spacing outcomes

Birth spacing outcomes	Follow-up
Inter-birth interval of < 21 months (primary outcome)	21 months
Inter-birth interval of < 24 months	24 months
Inter-birth interval of < 15 months	15 months
Inter-birth interval (continuous)	60 months

3.2.2. Construction of contraception outcomes

Table 3.2 summarizes outcomes related to the utilization of contraceptive methods. The primary data source used for the contraception outcomes will be Medicaid claims data. Medicaid claims capture contraceptive provision among Medicaid enrollees that takes place in the hospital before discharge (inpatient), during outpatient hospital visits, and during ambulatory clinical visits. We will rely primarily on Medicaid claims data because most postpartum contraceptive provision occurs after hospital discharge. However, we will also supplement Medicaid discharge data with hospital discharge data to capture any inpatient contraceptive provision that may be missing from Medicaid claims. Any family planning related counseling or service will include any diagnosis code, procedure code, CPT code, or HCPCS codes for contraceptive counseling, the intrauterine device, implant, injectable, contraceptive pill, patch, ring or diaphragm (See Table 3.3 below) and National Drug Codes for the contraceptive pill, patch, and ring identified by the Office of Population Affairs Contraceptive Care Measures. Receipt of a moderately effective method of contraception will include all of the above methods but will not include contraceptive counseling without provision of a contraceptive method. Immediate postpartum contraception will include diagnosis, procedure, CPT or HCPCS code for an intrauterine device or contraceptive implant.

Contraceptive counseling and/or contraceptive receipt within six weeks of hospital discharge will meet the criteria for the 6-week contraceptive outcomes. Contraceptive counseling and/or receipt within 12 months of the date of hospital discharge will meet the criteria for the 12-month contraceptive outcomes.

Table 3.2. Contraceptive outcomes

Variables	Follow-up
Any family planning related counseling or service	6 weeks
Received a highly or moderately effective method of contraception[viii]	6 weeks
Immediate postpartum long-acting reversible contraception	6 weeks
Any family planning related counseling or service	12 months
Received a highly or moderately effective method of contraception[16]	12 months
Postpartum intrauterine device insertion	12 months
Time to first family planning counseling or service (months from pregnancy)	24 months
Time to first utilization of highly effective contraceptive methods (months from discharge)	24 months

---

[viii] CDC defines highly effective contraception to include implant, Immediate Post-Partum-Long-Acting Reversible Contraception, Long-Acting Reversible Contraception, or sterilization and moderately effective contraception to include path, ring, diaphragm, injectables and contraceptive pills.

In Table 3.3 we define codes used to identify specific contraceptive methods that will be used in analyses. Codes will be identified in both Medicaid claims and hospital discharge records.

Table 3.3. Codes used to identify contraception

Contraceptive method	Diagnosis codes	Procedure	CPT	HCPCS
Female sterilization	Z30.2	0U574ZZ, 0U578ZZ, 0UL74CZ, 0UL74DZ, 0UL74ZZ, 0UL78DZ, 0UL78ZZ	58565, 58600, 58605, 58611, 58615, 58670, 58671	A4264
Intrauterine device	Z30.430, Z30.014	0UH97HZ, 0UH98HZ, 0UHC7HZ, 0UHC8HZ, 0UH90HZ	58300	J7300, J7301, J7302, S4989, Q0090, S4981, J7297, J7298
Contraceptive implant	Z30.017, Z30.46	0JHD0HZ, 0JHD3HZ, 0JHF0HZ, 0JHF3HZ	11981	J7306, J7307
Contraceptive injectable	Z30.013, Z30.42	--	--	J1050
Contraceptive pills, patch, ring	Z30.011, Z30.41, Z30.016, Z30.45, Z30.015, Z30.44	--	--	S4993, J7304, J7303
Diaphragm	--	--	57170	A4266, A4261

3.3. Timing of Study analyses

We will complete this analysis once all study births have occurred, and at least 24 months have elapsed following the last study birth. We will add additional lags to account for the time it takes for administrative records to be complete, sent to the study team, and matched to existing analytic files. Based on the estimated delivery date collected in the baseline survey, the latest expected study delivery date is November 7, 2020. As we define births within a 120-day window of the estimated gestational age as a study index birth, the latest possible date when a study index birth could occur is March 7, 2020. We will allow for twenty-four months of follow-up from the time of the final possible study index birth (March 7, 2022), six additional months for the study outcomes to be fully incorporated into South Carolina administrative data (September 7, 2023), and an additional three-month buffer for the administrative data to be matched with the study dataset (December 7, 2023). Therefore, we anticipate that analyses of these outcomes will begin in December 2023.

This study includes four birth spacing outcomes. The first three are birth interval indicators defined as having no subsequent live birth observed within 21 (primary outcome), 15 and 24 months (secondary outcomes) of the index birth. The fourth is a continuous outcome defined as the number of months before any subsequent birth within 60 months of the index birth. The analysis for this outcome will include only women who went on to have another birth within 60 months of their index birth. The first three of the birth spacing outcomes described in Table 3.1 will be reported in the main manuscript for the maternal life course objective. The continuous birth interval outcome will be included in a later manuscript which will include longer-term maternal outcomes.

3.4. Attrition and Missing Outcome Data

As described in section 1.4 some mother-babies will be matched to a childbirth-related Medicaid claim or discharge record but unmatched to a birth certificate record or fetal death vital record. We will conduct a sensitivity analysis that will include these study participants, in addition to the main sample following procedures defined in section 1.6.1. For mother-babies without a birth certificate or fetal death record, we will use hospital discharge data as the date of birth or fetal death.

3.5. Endogeneity of Outcome Data and Sensitivity Analyses

Because outcomes related to contraceptive uptake will rely primarily on Medicaid claims data, we cannot observe contraception use in outpatient settings among women who are not enrolled in Medicaid. As a non-expansion state, all women with Medicaid pregnancy coverage in South Carolina, except undocumented and recent immigrants, retain Medicaid coverage for at least 60 days following childbirth. Women who qualify for other payment categories (e.g. foster-care, disability), may retain coverage past 60 days. Because NFP may affect maintenance of Medicaid coverage past 60 days postpartum, including maintenance of enrollment in Medicaid Family Planning coverage, we may not be able to observe treatment and control group mothers in the Medicaid claims data at equal rates.

To determine whether continuous Medicaid coverage differs between treatment groups, we will first compare continuous Medicaid coverage of any type (coverage through the low-income families, qualified disabled workers, pregnancy and family planning eligibility pathways), and family planning Medicaid coverage specifically, between treatment and control groups at the three contraception outcome time points (6 weeks, 12 and 24 months). Continuous coverage will be measured using Medicaid enrollment data and will be defined as enrollment during all months, starting with the month of childbirth through 6 weeks, 12 and 24 months postpartum. It is also possible that while average Medicaid enrollment will not differ between the two groups, the type of women retaining coverage will be affected by NFP. Therefore, using a joint F-test, we

will also test whether there are differences in the characteristics of participants between control and treatment groups enrolled in Medicaid during the three-outcome time points (60 days, 12 months, 24 months).

When conducting the study analysis, we will divide outcomes between those that we observe within 60 days after childbirth (when nearly all women retain Medicaid), and those measured after 60 days postpartum. Our main analysis will include the full sample of women regardless of postpartum Medicaid coverage after 60 days, but we will also examine the outcomes in the subgroup of women who retained coverage for 24 months postpartum. If we find evidence that NFP affected average Medicaid enrollment or the type of woman enrolling in Medicaid, we will place more emphasis on the 6-week contraception outcomes when Medicaid coverage loss is minimal and more likely to be balanced between groups. Alternatively, if we find little evidence of differential coverage loss at 12 and 24 months, we will consider contraception outcomes at all three time points.

3.6. Subgroups

Our primary sub-group is described in McConnell et al. (2020).[1] Briefly, this subgroup comprises women who have an indicator of poor mental health, are under 19 years of age, or have not completed high school/received a General Education Development certificate by the time of enrollment.

In addition to the planned subgroup described above, we may also consider several other potential subgroups. We are interested in focusing more narrowly on groups who are most likely to benefit from increased access to family planning including teens, because pregnancies in this subgroup are more likely to be unintended than among adult women, and women who reported at baseline that they did not want to have another birth for at least two years. We are also interested in focusing on births that took place at least 24 months before the start of the COVID-19 pandemic (defined here as March 23, 2020). We expect that the pandemic altered the way that the NFP program was implemented. In addition, it is likely that the pandemic affected the availability of contraceptive services, fertility preferences, and employment; all factors on the causal pathway between NFP and birth outcomes.

[1] McConnell MA, Zhou RA, Martin MW, et al. Protocol for a randomized controlled trial evaluating the impact of the Nurse-Family Partnership's home visiting program in South Carolina on maternal and child health outcomes. *Trials*. 2020;21(1):997. doi:10.1186/s13063-020-04916-9

[2] Medicaid and CHIP Income Eligibility Limits for Pregnant Women, 2003-2020. KFF. Published April 1, 2020. Accessed January 7, 2021. https://www.kff.org/medicaid/state-indicator/medicaid-and-chip-income-eligibility-limits-for-pregnant-women/

[3] Medicaid, Children's Health Insurance Program, & Basic Health Program Eligibility Levels | Medicaid. Accessed January 7, 2021. https://www.medicaid.gov/medicaid/national-medicaid-chip-program-information/medicaid-childrens-health-insurance-program-basic-health-program-eligibility-levels/index.html#footnote1

[4] Medicaid Income Eligibility Limits for Parents, 2002-2020. KFF. Published April 1, 2020. Accessed January 15, 2021. https://www.kff.org/medicaid/state-indicator/medicaid-income-eligibility-limits-for-parents/

[5] Medicaid, Children's Health Insurance Program, & Basic Health Program Eligibility Levels | Medicaid. Accessed January 7, 2021. https://www.medicaid.gov/medicaid/national-medicaid-chip-program-information/medicaid-childrens-health-insurance-program-basic-health-program-eligibility-levels/index.html#footnote1

[6] Medicaid Income Eligibility Limits for Infants Ages 0 – 1, 2000-2020. KFF. Published April 1, 2020. Accessed January 7, 2021. https://www.kff.org/medicaid/state-indicator/medicaid-income-eligibility-limits-for-infants-ages-0-1/

[7] Medicaid Income Eligibility Limits for Children Ages 1 – 5, 2000-2020. KFF. Published April 1, 2020. Accessed January 7, 2021. https://www.kff.org/medicaid/state-indicator/medicaid-income-eligibility-limits-for-children-ages-1-5/

[8] Medicaid Income Eligibility Limits for Children Ages 6 – 18, 2000-2020. KFF. Published April 1, 2020. Accessed January 7, 2021. https://www.kff.org/medicaid/state-indicator/medicaid-income-eligibility-limits-for-children-ages-6-18/

[9] Puma MJ, Olsen RB, Bell SH, Price C. *What to Do When Data Are Missing in Group Randomized Controlled Trials. NCEE 2009-0049*. National Center for Education Evaluation and Regional Assistance; 2009. Accessed January 8, 2021. https://eric.ed.gov/?id=ED511781

[10] Han D, Khadka A, McConnell M, Cohen J. Association of Unexpected Newborn Deaths With Changes in Obstetric and Neonatal Process of Care. JAMA Netw Open. 2020;3(12):e2024589. doi:10.1001/jamanetworkopen.2020.24589

[11] Ford JB, Roberts CL, Algert CS, et al. Using hospital discharge data for determining neonatal morbidity and mortality: a validation study. BMC Health Serv Res. 2007;7(1):188. doi:10.1186/1472-6963-7-188

[12] How Does CDC Identify Severe Maternal Morbidity? | CDC. Published December 26, 2019. Accessed January 15, 2021. https://www.cdc.gov/reproductivehealth/maternalinfanthealth/smm/severe-morbidity-ICD.htm

[13] Bailey, B. A., McCook, J. G., Hodge, A., & McGrady, L. (2012). Infant birth outcomes among substance using women: why quitting smoking during pregnancy is just as important as quitting illicit drug use. Maternal and child health journal, 16(2), 414-422.

[14] Shah, P. S., & Shah, J. (2010). Maternal exposure to domestic violence and pregnancy and birth outcomes: a systematic review and meta-analyses. Journal of women's health, 19(11), 2017-2031.

[15] Mills, G., & Urban Institute. (2014). Understanding the rates, causes, and costs of churning in the supplemental nutrition assistance program (SNAP). United States Department of Agriculture, Food and Nutrition Service, Office of Policy Support.

[16] Jarlenski, M., Krans, E. E., Chen, Q., Rothenberger, S. D., Cartus, A., Zivin, K., & Bodnar, L. M. (2020). Substance use disorders and risk of severe maternal morbidity in the United States. Drug and alcohol dependence, 216, 108236.

[17] Davidov, D. M., Davis, S. M., Zhu, M., Afifi, T. O., Kimber, M., Goldstein, A. L., ... & Stocks, C. (2017). Intimate partner violence-related hospitalizations in Appalachia and the non-Appalachian United States. PLoS one, 12(9), e0184222.

[18] Schafer, S. D., Drach, L. L., Hedberg, K., & Kohn, M. A. (2008). Using diagnostic codes to screen for intimate partner violence in Oregon emergency departments and hospitals. Public Health Reports, 123(5), 628-635.

[19] Siega-Riz AM, Bodnar LM, Stotland NE, Stang J. The Current Understanding of Gestational Weight Gain Among Women with Obesity and the Need for Future Research. NAM Perspectives. Published online January 6, 2020. doi:10.31478/202001a

[20] Horovitz, J.L. & C. F. Manski (2000). "Nonparametric Analysis of Randomized Experiments with Missing Covariate and Outcome Data." Journal of the American Statistical Association, 95, 77-84.

[21] Fairbrother N, Young AH, Janssen P, Antony MM, Tucker E. Depression and anxiety during the perinatal period. BMC Psychiatry 2015; 15: 206.

[22] Kessler RC, Berglund P, Demler O, Jin R, Koretz D, Merikangas KR, et al. The epidemiology of major depressive disorder: Results from the national comorbidity survey replication (NCS-R). JAMA. 2003;289(23):3095.

[23] Sherman LJ, Ali MM. Diagnosis of Postpartum Depression and Timing and Types of Treatment Received Differ for Women with Private and Medicaid Coverage. Women's Health Issues 2018; 28: 524–9.

[24] Stewart, D. E., & Vigod, S. (2016). Postpartum depression. New England Journal of Medicine, 375(22), 2177–2186

[25] Saba DK, Levit KR, Elixhauser A. Hospital Stays Related to Mental Health, 2006. 2008; : 13.

[26] McCall-Hosenfeld JS, Phiri K, Schaefer E, Zhu J, Kjerulff K. Trajectories of Depressive Symptoms Throughout the Peri- and Postpartum Period: Results from the First Baby Study. J Womens Health 2002 2016; 25: 1112–21.

[27] Putnick DL, Sundaram R, Bell EM, et al. Trajectories of Maternal Postpartum Depressive Symptoms. Pediatrics 2020; published online Oct 1. DOI:10.1542/peds.2020-0857.

[28] Kuklina EV, Whiteman MK, Hillis SD, et al. An enhanced method for identifying obstetric deliveries: implications for estimating maternal morbidity. *Matern Child Health J*. 2008;12(4):469-477. doi:10.1007/s10995-007-0256-6

[29] Leonard SA, Main EK, Scott KA, Profit J, Carmichael SL. Racial and Ethnic Disparities in Severe Maternal Morbidity Prevalence and Trends. *Ann Epidemiol*. Published online February 28, 2019. doi:10.1016/j.annepidem.2019.02.007

[30] Howell EA, Brown H, Brumley J, et al. Reduction of Peripartum Racial and Ethnic Disparities: A Conceptual Framework and Maternal Safety Consensus Bundle. *Obstet Gynecol*. 2018;131(5):770-782. doi:10.1097/AOG.0000000000002475

[31] Kozhimannil KB, Trinacty CM, Busch AB, Huskamp HA, Adams AS. Racial and ethnic disparities in postpartum depression care among low-income women. Psychiatr Serv Wash DC 2011; 62: 619–25.

[32] Davenport MH, Meyer S, Meah VL, Strynadka MC, Khurana R. Moms Are Not OK: COVID-19 and Maternal Mental Health. Front Glob Womens Health 2020; 1. DOI:10.3389/fgwh.2020.00001.

[33] Schnitzer PG, Slusher PL, Kruse RL, Tarleton MM. Identification of ICD codes suggestive of child maltreatment. *Child Abuse Negl*. 2011;35(1):3-17. doi:10.1016/j.chiabu.2010.06.008

[34] Hooft A, Ronda J, Schaeffer P, Asnes AG, Leventhal JM. Identification of Physical Abuse Cases in Hospitalized Children: Accuracy of International Classification of Diseases Codes. *J Pediatr*. 2013;162(1):80-85. doi:10.1016/j.jpeds.2012.06.037

[35] Turner K. Well-Child Visits for Infants and Young Children. Am Fam Physician. 2018;98(6):347-353.

[36] Kling J, Liebman J, Katz L. Experimental Analysis of Neighborhood Effects. *Econometrica*. 2007;75(1):83-119.
