# Supplementary material for: Home Visits and the Use of Routine and Emergency Postpartum Care Among Low-Income People: A Secondary Analysis of a Randomized Clinical Trial
Source: JAMA Netw Open. 2024 Dec 23;7(12):e2451605. doi: 10.1001/jamanetworkopen.2024.51605 (PMC11667346; doi:10.1001/jamanetworkopen.2024.51605)
Supplement: Supplement 2. — eFigure 1. Participant Recruitment Flowchart eTable 1. Sample and Variable Algorithms eTable 2. Subgroup Definitions eTable 3. Match Rates Between Treatment And Control eTable 4. Percentage of Missing Values on Self-Reported Baseline Characteristics eTable 5. Program Participation Stratified by Whether Postpartum Period Was Affected by COVID-19 eTable 6. Participation in Medicaid Home Visits at 12 Weeks Post Partum eTable 7. Subgroup Treatment Effect Estimates eTable 8. Multiple Imputation Results eTable 9. Logistic Regression Results: Full Sample eTable 10. Logistic Regression Results: Subgroups [file jamanetwopen-e2451605-s002.pdf]

Supplemental Online Content

Rokicki S, Oviedo D, Perreault N, et al. Home visits and the use of routine and emergency postpartum care among low-income people: a secondary analysis of a randomized clinical trial. *JAMA Netw Open*. 2024;7(12):e2451605. doi:10.1001/jamanetworkopen.2024.51605

eFigure 1. Participant Recruitment Flowchart

eTable 1. Sample and Variable Algorithms

eTable 2. Subgroup Definitions

eTable 3. Match Rates Between Treatment And Control

eTable 4. Percentage of Missing Values on Self-Reported Baseline Characteristics

eTable 5. Program Participation Stratified by Whether Postpartum Period Was Affected by COVID-19

eTable 6. Participation in Medicaid Home Visits at 12 Weeks Postpartum

eTable 7. Subgroup Treatment Effect Estimates

eTable 8. Multiple Imputation Results

eTable 9. Logistic Regression Results: Full Sample

eTable 10. Logistic Regression Results: Subgroups

This supplemental material has been provided by the authors to give readers additional information about their work.

© 2024 Rokicki S et al. *JAMA Network Open.*

eFigure 1. Participant Recruitment Flowchart

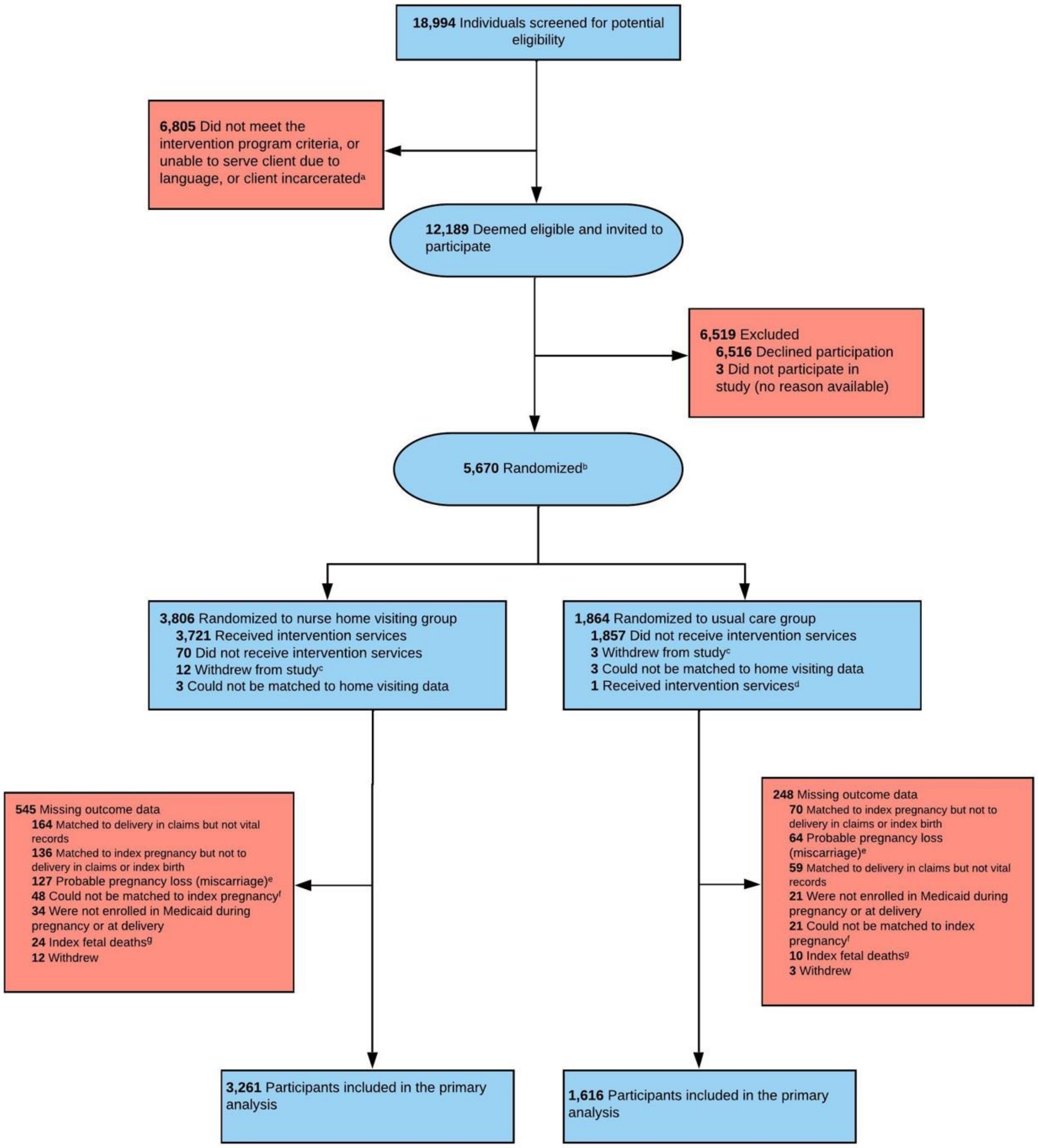

[a]The reasons participants were deemed ineligible were not systematically documented and so cannot be reported.

[b]Randomized in a 2:1 ratio to receive program services (intervention) or control, without stratification.

[c]Participation in the program was not recorded for participants who withdrew from the study.

[d]Participant may have received intervention services. Participant matched to a record in the Maternal, Infant, and Early Childhood Home Visiting program, indicating participation in the intervention program, but did not match to internal program records.

© 2024 Rokicki S et al. *JAMA Network Open.*

[e]Probable pregnancy loss was defined as having a diagnosis code for spontaneous abortion, ectopic pregnancy, or molar pregnancy in hospital discharge records or Medicaid claims within the expected delivery date and 42 weeks prior.

[f]Index pregnancy was defined as having at least 1 of the following: an index birth or fetal death, an indication of delivery in hospital discharge records or Medicaid claims within 120 days of the expected delivery date, a probable pregnancy loss in hospital discharge records or Medicaid claims, an antenatal care visit in Medicaid claims, or enrollment in Medicaid within the expected delivery date and 42 weeks prior.

[g]Index births were defined as matched births that appear in South Carolina vital records birth certificates within 120 days of the expected delivery date of the participant reported on the baseline survey.

© 2024 Rokicki S et al. *JAMA Network Open.*

eTable 1. Sample and variable algorithms

Algorithm	Definition
Sample selection	Participants with an "index" live birth in matched vital records within 120 days of the expected delivery date reported on the baseline survey and who were enrolled in Medicaid either during pregnancy or at delivery
Outcome study period	We include all claims that took place after the delivery discharge date and the first 12 weeks postpartum (for the 12 week outcomes) and between delivery discharge date and 1 year postpartum (for 1 year outcomes).
ED emergent and nonemergent visits	Using the NYU ED patch algorithm, each ICD discharge code is assigned a probability of belonging to the following categories: emergent ED care needed not preventable/avoidable; emergent ED care needed preventable/avoidable; emergent, primary care treatable; non-emergent.<br>Separate categories classify visits for injury, mental health, alcohol, and drug-related diagnoses, and unclassified. These were not used in our analyses.<br><br>We took the sum of these assigned probabilities to create two composite categories: emergent and non-emergent. The emergent category is the sum of the probabilities of the "emergent ED care needed not preventable/avoidable" and "emergent ED care needed preventable/avoidable" categories. The non-emergent category is the sum of the probabilities of the "emergent, primary care treatable" and "non-emergent" categories.<br><br>Since there are individuals with multiple observations per ED visit with differing ICD codes, we keep the maximum probability assigned to each category for that visit.<br><br>If any of the visits are assigned a >50% of being emergent, we call that visit emergent, if not, and it is assigned a >50% probability of being non-emergent, we call it non-emergent.<br><br>We then aggregate to the individual-level, taking the total number of emergent and non-emergent visits. Last, we create a binary variable indicating whether a mom had any emergent or any non-emergent visit as identified by the patch algorithm.

© 2024 Rokicki S et al. *JAMA Network Open.*

eTable 2. Subgroup definitions

Subgroup	Definition
Cesarean	Cesarean section - binary variable indicating cesarean section (final route)<br>Source: First check vital records (VR). If missing, any claim from discharge data. If missing, any claim from Medicaid data.<br><br>ICD codes: 10D00Z0, 10D00Z1, 10D00Z2<br>HCPCS codes: 59510, 59514, 59515, 59618, 59620, 59622
Chronic	• Asthma: J45<br>• Hypertension: I10, I11, I12, I13, I15, I674, N262<br>• Mood or anxiety disorders: F30, F31, F32, F33, F34, F39, F40, F41, F42, F43, F44, F45, F48<br>• Diabetes (type 1, 2 or other): E10, E11, E13, Z9641, Z4681, T85614A, T85624A, T85633A, T85694A<br>Source: claims
Complications	Complex pregnancy or delivery (source and ICD codes):<br>• Placenta previa (claims): O440, O441, O730<br>• preterm labor (VR + claims): O60<br>• gestational diabetes (VR + claims): O9981, O244<br>• gestational hypertension (VR + claims): O12, O13, O16<br>• preeclampsia (claims): O14, O15<br>• multiple gestation (VR + claims): Z372, Z373, Z374, Z375, Z376, Z377, O30<br>• hemorrhage (claims): D6959, O9903, O9913, O72, O721, O722, O723, O730, O731, I976<br>• infection (claims): O239, O8689, O864, T8579XA, T827XXA<br>• laceration (claims): O702, O703, O709, O717, O704, O7182, O719, O713, O714, O901<br>• operative injury (claims): O754, T81509A, T81519A, T81529A, T81539A, T80919A,<br>• uterine rupture (claims): O710, O711
Pre-covid	Mom experienced the period of delivery date-12 weeks postpartum before March 23, 2020.
Participants facing vulnerabilities	Socially vulnerable mothers are those with education less than high school diploma or GED, <19 years old, or mental health flag (PHQ-2 score >=2 or receipt of mental health treatment in previous year).
Non-Hispanic Black	Self-reported race and ethnicity as non-Hispanic Black

© 2024 Rokicki S et al. *JAMA Network Open.*

eTable 3. Match rates between treatment and control

	Usual care group	Nurse Home Visiting Group	Percentage point difference (SE)	p-value[a]
Index birth indicator - mother has a baby with matched VR within 120 days of EDD	1637/1861 (88.0%)	3295/3794 (86.8%)	1.1 (0.9)	0.24
Matched to VR index birth & Medicaid eligibility file	1621/1861 (87.1%)	3277/3794 (86.4%)	0.7 (1)	0.45
Matched to VR index birth & had Medicaid during pregnancy or at delivery [Analytical sample]	1616/1861 (86.8%)	3261/3794 (86%)	0.9 (1)	0.36
Matched to VR index birth & had Medicaid continuously for 60 days postpartum	1530/1861 (82.2%)	3095/3794 (81.6%)	0.6 (1.1)	0.56
Matched to VR index birth & had Medicaid continuously for 1 year postpartum	901/1861 (48.4%)	1810/3794 (47.7%)	0.7 (1.4)	0.62
Matched to VR index birth & had Medicaid during pregnancy or delivery & had at least one day affected by pandemic in the 12 weeks postpartum	307/1616 (19.0%)	597/3261 (18.31%)	0.7 (1.2)	0.56

Notes: Sample is all N=5655 enrolled mothers (n=1861 in control, n=3794 in treatment). VR=vital records. [a]P-values for the t-test were calculated using a two-tailed test. A significance level of α = 0.05 was used to determine statistical significance.

© 2024 Rokicki S et al. *JAMA Network Open.*

eTable 4. Percentage of Missing values on Self-reported Baseline Characteristics

Characteristics	Total (N=4877)	Nurse Home Visiting Group (N = 3261)	Usual Care Group (N = 1616)
Race/ethnicity	299/4877 (6.1%)	187/3261 (5.7%)	112/1616 (6.9%)
Reported interacting with the father of the child daily	21/4877 (0.4%)	14/3261 (0.4%)	7/1616 (0.4%)
Highest education level	20/4877 (0.4%)	14/3261 (0.4%)	6/1616 (0.4%)
Working for pay	2/4877 (0.0%)	1/3261 (0.0%)	1/1616 (0.1%)
Receiving one or more social service programs	101/4877 (2.1%)	64/3261 (2.0%)	37/1616 (2.3%)
Housing insecurity	9/4877 (0.2%)	5/3261 (0.2%)	4/1616 (0.2%)
Currently lives with parents	4/4877 (0.1%)	2/3261 (0.1%)	2/1616 (0.1%)
Received at least one antenatal care visit before time of survey (%)	57/4877 (1.2%)	37/3261 (1.1%)	20/1616 (1.2%)
Used hospital emergency room in past six months	3/4877 (0.1%)	0/3261 (0.0%)	3/1616 (0.2%)
Received mental health treatment in last year	8/4877 (0.2%)	3/3261 (0.1%)	5/1616 (0.3%)
Reported drinking alcohol in the three months before pregnancy	28/4877 (0.6%)	16/3261 (0.5%)	12/1616 (0.7%)
Reported smoking cigarettes in the three months before pregnancy	69/4877 (1.4%)	49/3261 (1.5%)	20/1616 (1.2%)
Depressive symptoms (PHQ-2 ≥ 3)	31/4877 (0.6%)	25/3261 (0.8%)	6/1616 (0.4%)
High stress (Perceived Stress Scale-4 ≥ 4)	66/4877 (1.4%)	44/3261 (1.3%)	22/1616 (1.4%)
Health self-reported as fair/poor	23/4877 (0.5%)	17/3261 (0.5%)	6/1616 (0.4%)
Body Mass Index (BMI)	159/4877 (3.3%)	97/3261 (3.0%)	62/1616 (3.8%)
Has access to a place for family planning or birth control	10/4877 (0.2%)	6/3261 (0.2%)	4/1616 (0.2%)
Reported a desire for more children in the future	1/4877 (0.0%)	0/3261 (0.0%)	1/1616 (0.1%)

© 2024 Rokicki S et al. *JAMA Network Open.*

eTable 5. Program participation stratified by whether postpartum period was affected by COVID-19

	12 weeks postpartum			1 year postpartum		
	Covid Unaffected	Covid Affected	Full Sample	Covid Unaffected	Covid Affected	Full Sample
**Extent of participation**						
At least one completed home visit	2031/2664 (76.2%)	449/597 (75.2%)	2480/3261 (76.1%)	1549/2034 (76.2%)	948/1227 (77.3%)	2497/3261 (76.6%)
At least one completed in-person home visit	2022/2664 (75.9%)	165/597 (27.6%)	2187/3261 (67.1%)	1547/2034 (76.1%)	695/1227 (56.6%)	2242/3261 (68.8%)
At least one completed telehealth home visit	471/2664 (17.7%)	435/597 (72.9%)	906/3261 (27.8%)	563/2034 (27.7%)	879/1227 (71.6%)	1442/3261 (44.2%)
At least one Medicaid home visit	192/2664 (7.2%)	10/597 (1.7%)	202/3261 (6.2%)	145/2034 (7.1%)	60/1227 (4.9%)	205/3261 (6.3%)
Number of completed home visits	4.5 (3.4)	4.7 (4.1)	4.6 (3.5)	12.3 (10.2)	13.9 (12.1)	12.9 (11)
Number of completed in-person home visits	4.2 (3.1)	0.9 (1.8)	3.6 (3.2)	11.5 (9.5)	4.8 (6)	9 (9)
Number of completed telehealth home visits	0.3 (1)	3.9 (3.7)	1 (2.3)	0.8 (2.1)	9.2 (9.3)	4 (7.2)
Home visit duration[a]	66.6 (30.6, N = 2031)	41.3 (18.8, N = 449)	62 (30.4, N = 2480)	65.2 (28, N = 1549)	46.3 (17.8, N = 948)	58 (26.3, N = 2497)
In-person home visit duration[a]	69.8 (32.1, N = 2022)	61.9 (30.4, N = 165)	69.2 (32.1, N = 2187)	67.9 (28.1, N = 1547)	63.9 (19.6, N = 694)	66.7 (25.8, N = 2241)
Telehealth home visit duration[a]	23.1 (13, N = 471)	35.1 (16.4, N = 435)	28.9 (15.9, N = 906)	22.3 (13.1, N = 563)	34.2 (14.2, N = 879)	29.6 (14.9, N = 1442)
**Length of NFP participation**	**Covid Unaffected**	**Covid Affected**	**Full Sample**			
Home visits continued to within 14 days of 12th week postpartum	1805/2664 (67.8%)	417/597 (69.8%)	2222/3261 (68.1%)			
Home visits continued to within 14 days of 1st year postpartum	1015/2034 (49.9%)	697/1227 (56.8%)	1712/3261 (52.5%)			

Notes: Sample is mothers with an index birth and Medicaid coverage any time during pregnancy and birth COVID-19 unaffected defined as those who experienced pregnancy-12 weeks postpartum before March 23, 2020. Visit-level data are aggregated at the individual participant level prior to analysis.
[a]Program metrics on duration of visit are reported for participants with at least one home visit in the specified postpartum period.

© 2024 Rokicki S et al. *JAMA Network Open.*

eTable 6. Participation in Medicaid home visits at 12 weeks postpartum

	Nurse Home Visiting Group	Usual Care Group
At least one Medicaid home visit [N (%)]	202/3261 (6.2%)	182/1616 (11.3%)

© 2024 Rokicki S et al. *JAMA Network Open.*

eTable 7. Subgroup treatment effect estimates

	Nurse Home Visiting Group	Usual Care Group	Unadjusted Between-Group Difference (95% CI)	Adjusted Between-Group Difference (95% CI)
**Panel A. Cesarean subgroup (n=1,531)**				
Routine postpartum follow-up	648/1011 (64.1%)	329/520 (63.3%)	0.8 (-4.3, 5.9)	0.5 (-4.6, 5.6)
At least one outpatient visit not PP check	29/1011 (2.9%)	9/520 (1.7%)	1.1 (-0.5, 2.8)	1.3 (-0.4, 3)
At least one emergency department visit without admission	222/1011 (22%)	125/520 (24%)	-2.1 (-6.5, 2.4)	-1.7 (-6.1, 2.8)
At least one hospitalization	52/1011 (5.1%)	25/520 (4.8%)	0.3 (-2, 2.7)	0.2 (-2.2, 2.5)
ED visit (emergent)	29/1011 (2.9%)	19/520 (3.7%)	-0.8 (-2.6, 1.1)	-1.1 (-2.9, 0.8)
ED visit (non-emergent, primary care treatable)	110/1011 (10.9%)	69/520 (13.3%)	-2.4 (-5.8, 1)	-1.8 (-5.2, 1.6)
**Panel B. Chronic disease subgroup (n=2,022)**				
Routine postpartum follow-up	917/1357 (67.6%)	451/665 (67.8%)	-0.2 (-4.6, 4.1)	-0.2 (-4.6, 4.2)
At least one outpatient visit not PP check	45/1357 (3.3%)	23/665 (3.5%)	-0.1 (-1.8, 1.5)	0 (-1.7, 1.8)
At least one emergency department visit without admission	329/1357 (24.2%)	160/665 (24.1%)	0.2 (-3.8, 4.2)	-0.5 (-4.5, 3.5)
At least one hospitalization	69/1357 (5.1%)	23/665 (3.5%)	1.6 (-0.3, 3.6)*	1.2 (-0.8, 3.2)
ED visit (emergent)	49/1357 (3.6%)	21/665 (3.2%)	0.5 (-1.2, 2.2)	0.3 (-1.5, 2)
ED visit (non-emergent, primary care treatable)	175/1357 (12.9%)	92/665 (13.8%)	-0.9 (-4.1, 2.2)	-1.2 (-4.4, 1.9)
**Panel C. Complications subgroup (n=3,019)**				
Routine postpartum follow-up	1287/2016 (63.8%)	639/1003 (63.7%)	0.1 (-3.5, 3.8)	0.4 (-3.2, 4.1)
At least one outpatient visit not PP check	58/2016 (2.9%)	25/1003 (2.5%)	0.4 (-0.9, 1.6)	0.5 (-0.7, 1.8)
At least one emergency department visit without admission	408/2016 (20.2%)	211/1003 (21%)	-0.8 (-3.9, 2.3)	-0.9 (-3.9, 2.1)
At least one hospitalization	94/2016 (4.7%)	32/1003 (3.2%)	1.5 (0, 3)*	1.4 (-0.1, 3)*
ED visit (emergent)	75/2016 (3.7%)	30/1003 (3%)	0.7 (-0.7, 2.1)	0.6 (-0.8, 2)
ED visit (non-emergent, primary care treatable)	212/2016 (10.5%)	122/1003 (12.2%)	-1.6 (-4, 0.7)	-1.5 (-3.9, 0.8)
**Panel D. Pre-COVID subgroup (n=3,973)**				
Routine postpartum follow-up	1708/2664 (64.1%)	842/1309 (64.3%)	-0.2 (-3.4, 3)	0.2 (-3, 3.4)
At least one outpatient visit not PP check	75/2664 (2.8%)	34/1309 (2.6%)	0.2 (-0.9, 1.3)	0.4 (-0.7, 1.5)
At least one emergency department visit without admission	516/2664 (19.4%)	288/1309 (22%)	-2.6 (-5.3, 0)*	-2.9 (-5.5, -0.2)**
At least one hospitalization	100/2664 (3.8%)	44/1309 (3.4%)	0.4 (-0.8, 1.6)	0.3 (-0.9, 1.6)
ED visit (emergent)	74/2664 (2.8%)	37/1309 (2.8%)	0 (-1.1, 1)	-0.2 (-1.2, 0.9)
ED visit (non-emergent, primary care treatable)	278/2664 (10.4%)	164/1309 (12.5%)	-2.1 (-4.2, 0)**	-2 (-4.1, 0.1)*

© 2024 Rokicki S et al. *JAMA Network Open.*

**Panel E. Vulnerable subgroup (n=2,269)**				
Routine postpartum follow-up	977/1541 (63.4%)	455/728 (62.5%)	0.9 (-3.4, 5.2)	0.3 (-3.9, 4.6)
At least one outpatient visit not PP check	49/1541 (3.2%)	21/728 (2.9%)	0.3 (-1.2, 1.8)	0.3 (-1.2, 1.8)
At least one emergency department visit without admission	315/1541 (20.4%)	161/728 (22.1%)	-1.7 (-5.3, 1.9)	-2.2 (-5.8, 1.4)
At least one hospitalization	64/1541 (4.2%)	23/728 (3.2%)	1 (-0.7, 2.7)	0.8 (-0.9, 2.5)
ED visit (emergent)	50/1541 (3.2%)	20/728 (2.7%)	0.5 (-1, 2)	0.3 (-1.2, 1.8)
ED visit (non-emergent, primary care treatable)	176/1541 (11.4%)	85/728 (11.7%)	-0.3 (-3.1, 2.6)	-0.3 (-3.1, 2.5)
**Panel F. Non-Hispanic Black subgroup (n=2,535)**				
Routine postpartum follow-up	1108/1697 (65.3%)	551/838 (65.8%)	-0.5 (-4.4, 3.5)	-0.5 (-4.4, 3.5)
At least one outpatient visit not PP check	52/1697 (3.1%)	26/838 (3.1%)	0 (-1.5, 1.4)	0 (-1.5, 1.4)
At least one emergency department visit without admission	344/1697 (20.3%)	184/838 (22%)	-1.7 (-5, 1.7)	-2.2 (-5.5, 1.2)
At least one hospitalization	62/1697 (3.7%)	26/838 (3.1%)	0.6 (-1, 2.1)	0.5 (-1, 2.1)
ED visit (emergent)	48/1697 (2.8%)	21/838 (2.5%)	0.3 (-1, 1.7)	0.2 (-1.1, 1.6)
ED visit (non-emergent, primary care treatable)	202/1697 (11.9%)	114/838 (13.6%)	-1.7 (-4.4, 1)	-1.9 (-4.6, 0.8)

© 2024 Rokicki S et al. *JAMA Network Open.*

eTable 8. Multiple imputation results

	Nurse Home Visiting Group	Usual Care Group	Unadjusted Between-Group Difference (95% CI)	Adjusted Between-Group Difference (95% CI)
**12-week outcomes**				
Routine postpartum follow-up	2079/3261 (63.8%)	1038/1616 (64.2%)	-0.5 (-3.3, 2.4)	-0.2 (-3.3, 2.9)
At least one outpatient visit not PP check	94/3261 (2.9%)	42/1616 (2.6%)	0.3 (-0.7, 1.3)	0.6 (-0.5, 1.6)
At least one emergency department visit without admission	601/3261 (18.4%)	333/1616 (20.6%)	-2.2 (-4.5, 0.2)*	-2.7 (-5.2, -0.1)**
At least one hospitalization	120/3261 (3.7%)	50/1616 (3.1%)	0.6 (-0.5, 1.7)	0.8 (-0.4, 2)
ED visit (emergent)[1]	92/3261 (2.8%)	44/1616 (2.7%)	0.1 (-0.9, 1.1)	0.1 (-1, 1.2)
ED visit (non-emergent, primary care treatable)[2]	317/3261 (9.7%)	186/1616 (11.5%)	-1.8 (-3.6, 0)*	-1.6 (-3.5, 0.4)
**1-year outcomes**				
At least one outpatient visit not PP check	353/3261 (10.8%)	164/1616 (10.1%)	0.7 (-1.2, 2.5)	0.7 (-1.3, 2.7)
At least one emergency department visit without admission	1578/3261 (48.4%)	799/1616 (49.4%)	-1.1 (-4, 1.9)	-1.5 (-4.6, 1.6)
At least one hospitalization	243/3261 (7.5%)	107/1616 (6.6%)	0.8 (-0.7, 2.4)	1.1 (-0.5, 2.8)
ED visit (emergent)[1]	289/3261 (8.9%)	143/1616 (8.8%)	0 (-1.7, 1.7)	0 (-1.9, 1.9)

eTable 9. Logistic regression results – full sample

	Nurse Home Visiting Group	Usual Care Group	Unadjusted Odds Ratios (95% CI)	Adjusted Odds Ratios (95% CI)
**12 week outcomes**				
Routine postpartum follow-up	1708/2664 (64.1%)	842/1309 (64.3%)	0.99 (0.86, 1.14)	1.01 (0.88, 1.16)
At least one outpatient visit not PP check	75/2664 (2.8%)	34/1309 (2.6%)	1.09 (0.72, 1.64)	1.16 (0.76, 1.75)
At least one emergency department visit without admission	516/2664 (19.4%)	288/1309 (22%)	0.85 (0.72, 1)*	0.83 (0.7, 0.98)**
At least one hospitalization	100/2664 (3.8%)	44/1309 (3.4%)	1.12 (0.78, 1.61)	1.11 (0.77, 1.6)
ED visit (emergent)[1]	74/2664 (2.8%)	37/1309 (2.8%)	0.98 (0.66, 1.47)	0.94 (0.62, 1.41)
ED visit (non-emergent, primary care treatable)[2]	278/2664 (10.4%)	164/1309 (12.5%)	0.81 (0.66, 1)**	0.82 (0.66, 1.01)*
**1 year outcomes**				
At least one outpatient visit not PP check	252/2664 (9.5%)	127/1309 (9.7%)	0.97 (0.78, 1.22)	1.03 (0.82, 1.29)
At least one emergency department visit without admission	1321/2664 (49.6%)	656/1309 (50.1%)	0.98 (0.86, 1.12)	0.98 (0.85, 1.13)
At least one hospitalization	207/2664 (7.8%)	88/1309 (6.7%)	1.17 (0.9, 1.51)	1.17 (0.9, 1.52)
ED visit (emergent)[1]	234/2664 (8.8%)	117/1309 (8.9%)	0.98 (0.78, 1.24)	0.96 (0.76, 1.22)
ED visit (non-emergent, primary care treatable)[2]	978/2664 (36.7%)	484/1309 (37%)	0.99 (0.86, 1.13)	0.99 (0.86, 1.15)

© 2024 Rokicki S et al. *JAMA Network Open.*

eTable 10. Logistic regression results – subgroups

	Nurse Home Visiting Group	Usual Care Group	Unadjusted Between-Group Difference (95% CI)	Adjusted Between-Group Difference (95% CI)
**Panel A. Cesarean subgroup (n=1,531)**				
Routine postpartum follow-up	648/1011 (64.1%)	329/520 (63.3%)	1.04 (0.83, 1.29)	1.02 (0.81, 1.29)
At least one outpatient visit not PP check	29/1011 (2.9%)	9/520 (1.7%)	1.7 (0.8, 3.6)	1.8 (0.8, 4)
At least one emergency department visit without admission	222/1011 (22%)	125/520 (24%)	0.9 (0.7, 1.1)	0.9 (0.7, 1.2)
At least one hospitalization	52/1011 (5.1%)	25/520 (4.8%)	1.1 (0.7, 1.8)	1.1 (0.6, 1.8)
ED visit (emergent)	29/1011 (2.9%)	19/520 (3.7%)	0.8 (0.4, 1.4)	0.7 (0.4, 1.3)
ED visit (non-emergent, primary care treatable)	110/1011 (10.9%)	69/520 (13.3%)	0.8 (0.6, 1.1)	0.8 (0.6, 1.2)
**Panel B. Chronic disease subgroup (n=2,022)**				
Routine postpartum follow-up	917/1357 (67.6%)	451/665 (67.8%)	0.99 (0.81, 1.21)	0.99 (0.81, 1.21)
At least one outpatient visit not PP check	45/1357 (3.3%)	23/665 (3.5%)	0.96 (0.57, 1.6)	1.03 (0.61, 1.75)
At least one emergency department visit without admission	329/1357 (24.2%)	160/665 (24.1%)	1.01 (0.81, 1.25)	0.97 (0.78, 1.22)
At least one hospitalization	69/1357 (5.1%)	23/665 (3.5%)	1.5 (0.92, 2.42)	1.34 (0.82, 2.2)
ED visit (emergent)	49/1357 (3.6%)	21/665 (3.2%)	1.15 (0.68, 1.93)	1.1 (0.64, 1.9)
ED visit (non-emergent, primary care treatable)	175/1357 (12.9%)	92/665 (13.8%)	0.92 (0.7, 1.21)	0.9 (0.68, 1.19)
**Panel C. Complications subgroup (n=3,019)**				
Routine postpartum follow-up	1287/2016 (63.8%)	639/1003 (63.7%)	1.01 (0.86, 1.18)	1.02 (0.86, 1.2)
At least one outpatient visit not PP check	58/2016 (2.9%)	25/1003 (2.5%)	1.16 (0.72, 1.86)	1.22 (0.75, 1.97)
At least one emergency department visit without admission	408/2016 (20.2%)	211/1003 (21%)	0.95 (0.79, 1.15)	0.94 (0.78, 1.14)
At least one hospitalization	94/2016 (4.7%)	32/1003 (3.2%)	1.48 (0.99, 2.23)*	1.46 (0.96, 2.21)*
ED visit (emergent)	75/2016 (3.7%)	30/1003 (3%)	1.25 (0.81, 1.93)	1.22 (0.78, 1.89)
ED visit (non-emergent, primary care treatable)	212/2016 (10.5%)	122/1003 (12.2%)	0.85 (0.67, 1.08)	0.86 (0.67, 1.09)
**Panel D. Pre-COVID subgroup (n=3,973)**				
Routine postpartum follow-up	1708/2664 (64.1%)	842/1309 (64.3%)	0.99 (0.86, 1.14)	1.01 (0.88, 1.16)
At least one outpatient visit not PP check	75/2664 (2.8%)	34/1309 (2.6%)	1.09 (0.72, 1.64)	1.16 (0.76, 1.75)
At least one emergency department visit without admission	516/2664 (19.4%)	288/1309 (22%)	0.85 (0.72, 1)*	0.83 (0.7, 0.98)**
At least one hospitalization	100/2664 (3.8%)	44/1309 (3.4%)	1.12 (0.78, 1.61)	1.11 (0.77, 1.6)
ED visit (emergent)	74/2664 (2.8%)	37/1309 (2.8%)	0.98 (0.66, 1.47)	0.94 (0.62, 1.41)
ED visit (non-emergent, primary care treatable)	278/2664 (10.4%)	164/1309 (12.5%)	0.81 (0.66, 1)**	0.82 (0.66, 1.01)*

© 2024 Rokicki S et al. *JAMA Network Open.*

**Panel E. Vulnerable subgroup (n=2,269)**				
Routine postpartum follow-up	977/1541 (63.4%)	455/728 (62.5%)	1.04 (0.87, 1.25)	1.02 (0.84, 1.23)
At least one outpatient visit not PP check	49/1541 (3.2%)	21/728 (2.9%)	1.11 (0.66, 1.86)	1.12 (0.66, 1.91)
At least one emergency department visit without admission	315/1541 (20.4%)	161/728 (22.1%)	0.9 (0.73, 1.12)	0.87 (0.69, 1.08)
At least one hospitalization	64/1541 (4.2%)	23/728 (3.2%)	1.33 (0.82, 2.16)	1.28 (0.78, 2.12)
ED visit (emergent)	50/1541 (3.2%)	20/728 (2.7%)	1.19 (0.7, 2.01)	1.08 (0.63, 1.85)
ED visit (non-emergent, primary care treatable)	176/1541 (11.4%)	85/728 (11.7%)	0.98 (0.74, 1.28)	0.98 (0.73, 1.3)
**Panel F. Non-Hispanic Black subgroup (n=2,535)**				
Routine postpartum follow-up	1108/1697 (65.3%)	551/838 (65.8%)	0.98 (0.82, 1.17)	0.98 (0.82, 1.17)
At least one outpatient visit not PP check	52/1697 (3.1%)	26/838 (3.1%)	0.99 (0.61, 1.59)	1.01 (0.62, 1.64)
At least one emergency department visit without admission	344/1697 (20.3%)	184/838 (22%)	0.9 (0.74, 1.11)	0.88 (0.71, 1.08)
At least one hospitalization	62/1697 (3.7%)	26/838 (3.1%)	1.18 (0.74, 1.89)	1.14 (0.71, 1.83)
ED visit (emergent)	48/1697 (2.8%)	21/838 (2.5%)	1.13 (0.67, 1.9)	1.06 (0.62, 1.81)
ED visit (non-emergent, primary care treatable)	202/1697 (11.9%)	114/838 (13.6%)	0.86 (0.67, 1.1)	0.85 (0.66, 1.09)

© 2024 Rokicki S et al. *JAMA Network Open.*
