# Supplementary material for: Home Visits and the Use of Routine and Emergency Postpartum Care Among Low-Income People: A Secondary Analysis of a Randomized Clinical Trial
Source: JAMA Netw Open. 2024 Dec 23;7(12):e2451605. doi: 10.1001/jamanetworkopen.2024.51605 (PMC11667346; doi:10.1001/jamanetworkopen.2024.51605)
Supplement: Supplement 3. — Data Sharing Statement [file jamanetwopen-e2451605-s003.pdf]

Data Sharing Statement

Rokicki. Home Visits and the Use of Routine and Emergency Postpartum Care Among Low-Income People. *JAMA Netw Open*. Published December 23, 2024.
doi:10.1001/jamanetworkopen.2024.51605

Data
Additional Information: AEA RCT Registry:AEARCTR-0001039
Clinicaltrials.gov:NCT03360539
Data available: No

Additional Information
Explanation for why data not available: Because of our data use agreements and consent processes, we are not able to publicly share trial data.
